# Stability of working memory in continuous attractor networks under the control of short-term plasticity

**DOI:** 10.1371/journal.pcbi.1006928

**Published:** 2019-04-19

**Authors:** Alexander Seeholzer, Moritz Deger, Wulfram Gerstner

**Affiliations:** 1 School of Computer and Communication Sciences and School of Life Sciences, Brain Mind Institute, École Polytechnique Fédérale de Lausanne, Lausanne, Switzerland; 2 Institute for Zoology, Faculty of Mathematics and Natural Sciences, University of Cologne, Cologne, Germany; Hebrew University, ISRAEL

## Abstract

Continuous attractor models of working-memory store continuous-valued information in continuous state-spaces, but are sensitive to noise processes that degrade memory retention. Short-term synaptic plasticity of recurrent synapses has previously been shown to affect continuous attractor systems: short-term facilitation can stabilize memory retention, while short-term depression possibly increases continuous attractor volatility. Here, we present a comprehensive description of the combined effect of both short-term facilitation and depression on noise-induced memory degradation in one-dimensional continuous attractor models. Our theoretical description, applicable to rate models as well as spiking networks close to a stationary state, accurately describes the slow dynamics of stored memory positions as a combination of two processes: (i) diffusion due to variability caused by spikes; and (ii) drift due to random connectivity and neuronal heterogeneity. We find that facilitation decreases both diffusion and directed drifts, while short-term depression tends to increase both. Using mutual information, we evaluate the combined impact of short-term facilitation and depression on the ability of networks to retain stable working memory. Finally, our theory predicts the sensitivity of continuous working memory to distractor inputs and provides conditions for stability of memory.

## Author summary

The ability to transiently memorize positions in the visual field is crucial for behavior. Models and experiments have shown that such memories can be maintained in networks of cortical neurons with a continuum of possible activity states, that reflects the continuum of positions in the environment. However, the accuracy of positions stored in such networks will degrade over time due to the noisiness of neuronal signaling and imperfections of the biological substrate. Previous work in simplified models has shown that synaptic short-term plasticity could stabilize this degradation by dynamically up- or down-regulating the strength of synaptic connections, thereby “pinning down” memorized positions. Here, we present a general theory that accurately predicts the extent of this “pinning down” by short-term plasticity in a broad class of biologically plausible network models, thereby untangling the interplay of varying biological sources of noise with short-term plasticity. Importantly, our work provides a novel theoretical link from the microscopic substrate of working memory—neurons and synaptic connections—to observable behavioral correlates, for example the susceptibility to distracting stimuli.

## Introduction

Information about past environmental stimuli can be stored and retrieved seconds later from working memory [1, 2]. Strikingly, this transient storage is achieved for timescales of seconds with neurons and synapse transmission operating mostly on time scales of tens of milliseconds and shorter [3]. An influential hypothesis of neuroscience is that working memory emerges from recurrently connected cortical neuronal networks: memories are retained by self-generating cortical activity through positive feedback [4–7], thereby bridging the time scales from milliseconds (neuronal dynamics) to seconds (behavior).

Sensory stimuli are often embedded in a physical continuum: for example, positions of objects in the visual field are continuous, as are frequencies of auditory stimuli, or the position of somatosensory stimuli on the body. Ideally, the organization of cortical working memory circuits should reflect the continuous nature of sensory information [3]. A class of cortical working memory models able to store continuously structured information is that of *continuous attractors*, characterized by a continuum of meta-stable states, which can be used to retain memories over delay periods much longer than those of the single network constituents [8]. Continuous attractors were proposed as theoretical models for cortical working memory [9–11], path integration [12–14], and other cortical functions [15–17] (see e.g. [3, 18–21] for recent reviews), well before experimental evidence was found in cortical networks [22] and the limbic system [18, 23]. The one-dimensional ring-attractor in the fly responsible for self-orientation [24, 25] is a particularly intriguing example.

Continuous attractor models have been successfully employed in the context of visuospatial working memory to explain behavioral performance [26–29], to predict the effects of neuromodulation [30, 31], or the implications of cognitive impairment [32, 33]. However, in networks with heterogeneities, the continuum of memory states quickly breaks down, since noise and heterogeneities break, transiently or permanently, the crucial symmetry necessary for continuous attractors [10, 11, 13, 16, 34–40]. For example, the stochasticity of neuronal spiking (“fast noise”) leads to transient asymmetries that randomly displace encoded memories along the continuum of states [10, 11, 35, 37, 39, 40], leading, averaged over many trials, to *diffusion* of encoded information. More drastically, introducing fixed asymmetries (“frozen noise”) due to network heterogeneities causes a *directed drift* of memories and a collapse of the continuum of attractive states to a set of discrete states. Examples of heterogeneities in biological scenarios include the sparsity of recurrent connections [13, 36], or randomness in neuronal parameters [36] and values of recurrent weights [16, 34, 38]. Since both (fast) noise and heterogeneities are expected in cortical settings, the feasibility of continuous attractors as computational systems of the brain has been called into question [3, 6, 41].

The question then arises, whether short-term plasticity of recurrent synaptic connections can rescue the feasibility of continuous attractor models. In particular, short-term depression has a strong effects on the directed drift of attractor states in rate models [42, 43], but no strong conclusions were drawn in a spiking network implementation [44]. Short-term facilitation, on the other hand, increases the retention time of memories in continuous attractor networks with noise-free [38] and, as shown parallel to this study, noisy [45] rate neurons. In simulations of continuous attractors implemented with spiking neurons for a fixed set of parameters, facilitation was reported to cause slow drift [46, 47] and a reduced amount of diffusion [47]. However, despite the large number of existing studies, several fundamental questions remain unanswered. What are the quantitative effects of short-term facilitation in more complex neuronal models and across facilitation parameters? How does short-term depression influence the strength of diffusion and drift, and how does it interplay with facilitation? Do phenomena reported in rate networks persist in spiking networks? Finally, can a single theory be used to predict all of the effects observed in simulations?

Here, we present a comprehensive description of the effects of short-term facilitation and depression on noise-induced displacement of one-dimensional continuous attractor models. Extending earlier theories for diffusion [39, 40, 45] and drift [38], we derive predictions of the amount of diffusion and drift in ring-attractor models of randomly firing neurons with short-term plasticity, providing, for the first time, a general description of bump displacement in the presence of both short-term facilitation and depression. Our theory is formulated as a rate model with noise, but since the gain-function of the rate model can be chosen to match that of integrate-and-fire models, our theory is also a good approximation for a large class of heterogeneous networks of integrate-and-fire models as long as the network as a whole is close to a stationary state. The theoretical predictions of the noisy rate model are validated against simulations of ring-attractor networks realized with spiking integrate-and-fire neurons. In both theory and simulation, we find that facilitation and depression play antagonistic roles: facilitation tends to *decrease both diffusion and drift* while depression *increases both*. We show that these combined effects can still yield reduced diffusion and drift, which increases the retention time of memories. Importantly, since our theory is, to a large degree, independent of the microscopic network configurations, it can be related to experimentally observable quantities. In particular, our theory predicts the sensitivity of networks with short-term plasticity to distractor stimuli.

## Results

**Fig 1:**
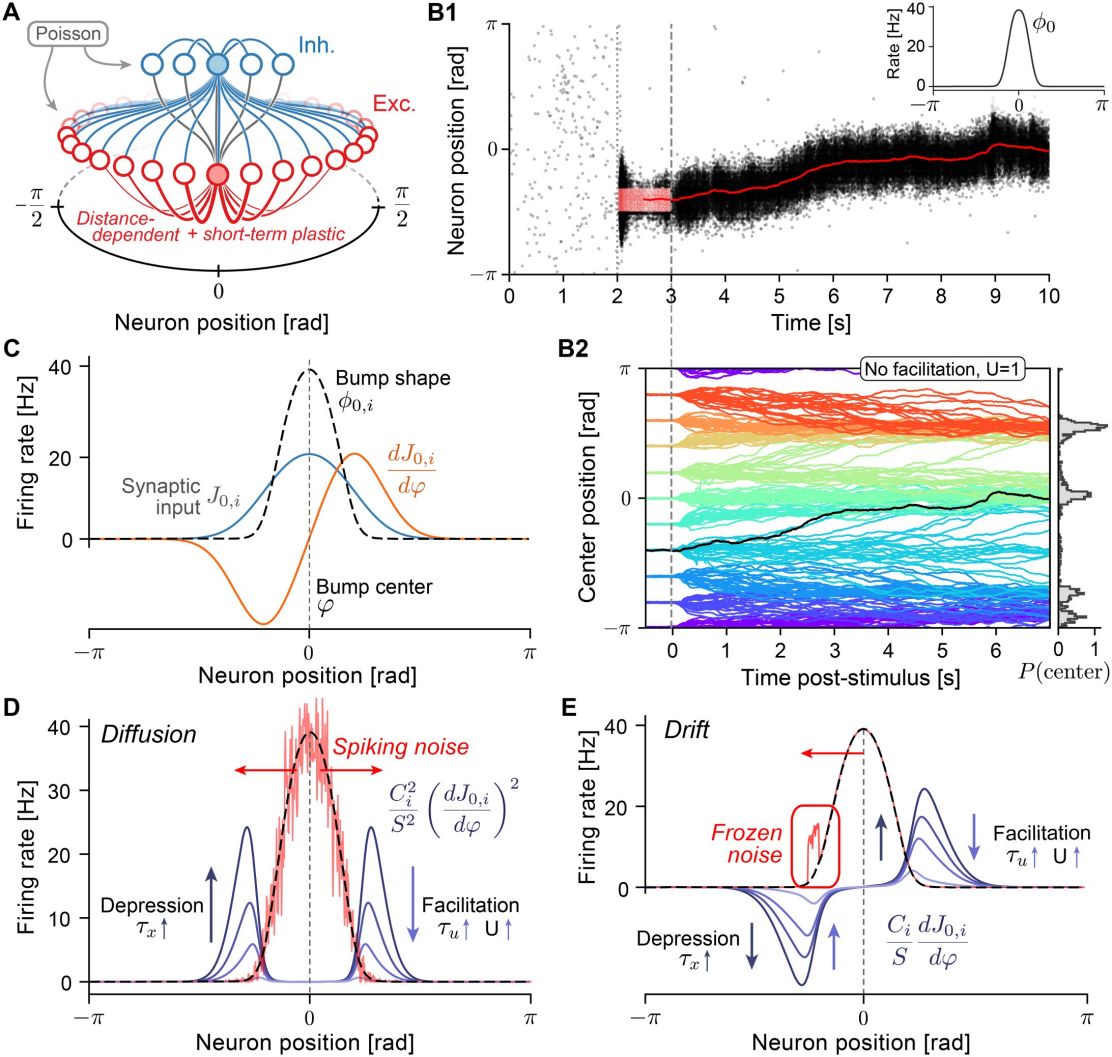
Drift and diffusion in ring-attractor models with short-term plasticity. **A** Excitatory (E) neurons (red circles) are distributed on a ring with coordinates in [−*π*, *π*]. Excitatory-to-excitatory (E-E) connections (red lines) are distance-dependent, symmetric, and subject to short-term plasticity (facilitation and depression, see Eq (3)). Inhibitory (I) neurons (blue circles) project to all E and I neurons (blue lines) and receive connection from all E neurons (gray lines). Only outgoing connections from shaded neurons are displayed. In simulations with integrate-and-fire neurons, each neuron also receives noisy excitatory spike input generated by independent homogeneous Poisson processes. **B1** Example simulation: E neurons fire asynchronously and irregularly at low rates until (dotted line) a subgroup of E neurons is stimulated (external cue), causing them to spike at elevated rates (red dots, input was centered at 0, starting at *t* = 2*s* for 1*s*). During and after (dashed line) the stimulus, a bump state of elevated activity forms and sustains itself after the external cue is turned off. The spatial center of the population activity is estimated from the momentary firing rates (red line, plotted from *t* = 2.5*s* onward). Inset: Activity profile in the bump state, centered at 0. **B2** Center positions of 20 repeated spiking simulations for 10 different initial cue positions each for a network with short-term depression (*U* = 1, *τ*_*x*_ = 150*ms*). Random E-E connections (with connection probability *p* = 0.5) lead to directed drift in addition to diffusion. Right: Normalized histogram (200 bins) of final positions at time *t* = 13.5. **C** Illustration of quantities used in theoretic calculations. Neurons in the bump fire at rates *ϕ*_0,*i*_ (dashed black line, compare to B1, inset) due to the steady-state synaptic input *J*_0,*i*_ (blue line). Movement of the bump center causes a change of the synaptic input $\frac{dJ_{0,i}}{d\varphi }$ (orange line). **D** Diffusion along the attractor manifold is calculated (see Eq (5)) as a weighted sum of the neuronal firing rates in the bump state (dashed black line). Spiking noise (red line) is illustrated as a random deviation from the mean rate with variance proportional to the rate. The symmetric weighting factors (blue lines show $\frac{C_{i}^{2}}{S^{2}}(\frac{dJ_{0,i}}{d\varphi }{)}^{2}$ for varying *U*) are non-zero at the flanks of the firing rate profile. Stronger short-term depression and weaker facilitation increase the magnitude of weighting factors. **E** Deterministic drift is calculated as a weighted sum (see Eq (7)) of systematic deviations of firing rates from the bump state (frozen noise): a large positive firing rate deviation in the left flank (red line) will cause movement of the center position to the left (red arrow) because the weighting factors (blue lines show $\frac{C_{i}}{S}\frac{dJ_{0,i}}{d\varphi }$ for varying *U*) are asymmetric.

We investigated, in theory and simulations, the effects of short-term synaptic plasticity (STP) on the dynamics of ring-attractor models consisting of *N* excitatory neurons with distance-dependent and symmetric excitation, and global (uniform) inhibition provided by a population of inhibitory neurons (Fig 1A). For simplicity, we describe neurons in terms of firing rates, but our theory can be mapped to more complex neurons with spiking dynamics. An excitatory neuron *i* with 0 ≤ *i* < *N* is assigned an angular position $\theta_{i}=\frac{2\pi }{N}i-\pi \in [-\pi ,\pi )$, where we identify the bounds of the interval to form a ring topology (Fig 1A). The firing rate *ϕ*_*i*_ (in units of Hz) for each excitatory neuron *i* (0 ≤ *i* < *N* − 1) is given as a function of the neuronal input:

$$\begin{array}{c} \phi_{i}\left(t\right)=F\left(J_{i}\left(t\right)+J_{\text{inh}}\right). \end{array}$$ (1)

Here, the input-output relation *F* relates the dimensionless excitatory *J*_*i*_ and inhibitory *J*_inh_ inputs of neuron *i* to its firing rate. This represents a rate-based simplification of the possibly complex underlying neuronal dynamics [48]. We assume that the excitatory input *J*_*i*_(*t*) to neuron *i* at time *t* is given by a sum over all presynaptic neurons

$$\begin{array}{c} J_{i}\left(t\right)=\sum_{j=0}^{N-1}w_{ij}s_{j}\left(t\right), \end{array}$$ (2)

where *w*_*ij*_*s*_*j*_(*t*) describes the total activation of synaptic input from the presynaptic neuron *j* onto neurons *i*. The maximal strength *w*_*ij*_ of recurrent excitatory-to-excitatory connections is chosen to be local in the angular arrangement of neurons, such that connections are strongest to nearby excitatory neurons (Fig 1A, red lines). The momentary input depends also on the synaptic activation variables *s*_*j*_, to be defined below. Finally, connections to and from inhibitory neurons are assumed to be uniform and global (all-to-all) (Fig 1A, blue lines), thereby providing non-selective inhibitory input *J*_inh_ to excitatory neurons.

As a model of STP, we assume that excitatory-to-excitatory connections are subject to short-term facilitation and depression, which we implemented using a widely adopted model of short-term synaptic plasticity [49]. The *outgoing* *synaptic activations* *s*_*j*_ of neuron *j* are modeled by the following system of ordinary differential equations:

$$\begin{array}{ccc} {\dot{s}}_{j} & = & -\frac{s_{j}}{\tau_{s}}+u_{j}x_{j}\phi_{j},\\ {\dot{u}}_{j} & = & -\frac{u_{j}-U}{\tau_{u}}+U\left(1-u_{j}\right)\phi_{j},\\ {\dot{x}}_{j} & = & -\frac{x_{j}-1}{\tau_{x}}-u_{j}x_{j}\phi_{j}. \end{array}$$ (3)

The synaptic time scale *τ*_*s*_ governs the decay of the synaptic activations. The timescale of recovery *τ*_*x*_ is the main parameter of depression. While the recovery from facilitation is controlled by the timescale *τ*_*u*_, the parameter 0 < *U* ≤ 1 controls the baseline strength of unfacilitated synapses as well as the timescale of their strengthening. For fixed *τ*_*u*_, we consider smaller values of *U* to lead to a “stronger” effect of facilitation, and take *U* = 1 as the limit of non-facilitating synapses.

As a reference implementation of this model, we simulated networks of spiking conductance-based leaky-integrate-and-fire (LIF) neurons with (spike-based) short-term plastic synaptic transmission (Fig 1B1, see *Spiking network model* in Materials and methods for details). For these networks, under the assumption that neurons fire with Poisson statistics and the network is in a stationary state, neuronal firing can be approximated by the input-output relation *F* of Eq (1) [50, 51] (see *Firing rate approximation* in Materials and methods), which allows us to map the network into the general framework of Eqs (1) and (2). In the stationary state, synaptic depression will lead to a saturation of the synaptic activation variables *s*_*j*_ at a constant value as firing rates increase. This nonlinear behavior enables spiking networks to implement bi-stable attractor dynamics with relatively low firing rates [46, 52] similar to saturating NMDA synapses [11, 47]. Since we found that without depression (for *τ*_*x*_ → 0) the bump state was not stable at low firing rates (in agreement with [52]), we always keep the depression timescale *τ*_*x*_ at positive values.

Particular care was taken to ensure that networks display nearly identical bump shapes (similar to Fig 1B1, inset; see also S1 Fig), which required the re-tuning of network parameters (recurrent conductance parameters and the width of distance-dependent connections; see *Optimization of network parameters* in Materials and methods) for each combination of the STP parameters above.

Simulations with spiking integrate-and-fire neurons generally show a bi-stability between a *non-selective* state and a *bump* state. In the non-selective state, all excitatory neurons emit action potentials asynchronously and irregularly at roughly identical and low firing rates (Fig 1B1, left of dotted line). The bump state can be evoked by stimulating excitatory neurons localized around a given position by additional external input (Fig 1B1, red dots). After the external cue is turned off, a self-sustained firing rate profile (“bump”) emerges (Fig 1B1, right of dashed line, and inset) that persists until the network state is again changed by external input. For example, a short and strong uniform excitatory input to all excitatory neurons causes a transient increase in inhibitory feedback that is strong enough to return the network to the uniform state [11].

During the bump state, fast fluctuations in the firing of single neurons transiently break the perfect symmetry of the firing rate profile and introduce small random displacements along the attractor manifold, which become apparent as a random walk of the center position. If the simulation is repeated for several trials, the bump has the same shape in each trial, but information on the center position is lost in a *diffusion*-like process. We additionally included varying levels of biologically plausible sources of heterogeneity (frozen noise) in our networks: *random connectivity* between excitatory neurons (E-E) and heterogeneity of the single neuron properties of the excitatory population [36], realized as a random distribution of leak reversal potentials. Heterogeneities makes the bump *drift* away from its initial position in a directed manner. For example, the bump position in the randomly connected (*p* = 0.5) network of Fig 1B1 shows a clear upwards drift towards center positions around 0. Repeated simulations of the same attractor network with bumps initialized at different positions provide a more detailed picture of the combined drift and diffusion dynamics: bump center trajectories systematically are biased towards a few stable fixed points (Fig 1B2) around which they are distributed for longer simulation times (histogram in Fig 1B2, *t* = 13.5*s*). The theory developed in this paper aims at analyzing the above phenomena of drift and diffusion of the bump center.

### Theory of diffusion and drift with short-term plasticity

To untangle the observed interplay between diffusion and drift and investigate the effects of short-term plasticity, we derived a theory that reduces the microscopic network dynamics to a simple one-dimensional stochastic differential equation for the bump state. The theory yields analytical expressions for diffusion coefficients and drift fields, that depend on short-term plasticity parameters, the shape of the firing rate profile of the bump, as well as the neuron model chosen to implement the attractor.

First, we *assume* that the system of Eq (3) together with the network Eqs (1) and (2) has a 1-dimensional manifold of meta-stable states, i.e. the network is a ring-attractor network as described in the introduction. This entails, that the network dynamics permit the existence of a family of solutions that can be described as a self-sustained and symmetric bump of firing rates *ϕ*_0,*i*_(*φ*) = *F*(*J*_0,*i*_(*φ*)) with corresponding inputs *J*_0,*i*_(*φ*) (for 0 ≤ *i* < *N*). Importantly, the center *φ* of the bump can be located at any arbitrary position $\varphi \in \{\frac{j}{N}2\pi -\pi |0\leq j<N\}$. For example, if *ϕ*_0,*i*_(0) is a solution with input *J*_0,*i*_(0), then $\phi_{0,i+1}\left(\frac{2\pi }{N}\right)$ is also a solution with input $J_{0,i+1}\left(\frac{2\pi }{N}\right)$. This solution is illustrated in Fig 1C for a bump centered at *φ* = 0. Second, we assume that the number *N* of excitatory neurons is large (*N* → ∞), such that we can think of the possible positions *φ* as a continuum. Third, we assume that network heterogeneities are small enough to capture their effect as a linear (first order) perturbation to the stable bump state. Our final assumption is that neuronal firing is noisy, with spike counts distributed as Poisson processes, and that we are able to replace the shot-noise of Poisson spiking by white Gaussian noise with the same mean and autocorrelation, similar to earlier work [39, 53]; see *Diffusion* in Materials and methods, and Discussion. Under these assumptions, we are able to reduce the network dynamics to a **one-dimensional Langevin equation**, describing the dynamics of the center *φ*(*t*) of the firing rate profile (see *Analysis of drift and diffusion with STP* in Materials and methods):

$$\begin{array}{c} \dot{\varphi }=\sqrt{B}\eta \left(t\right)+A\left(\varphi \right). \end{array}$$ (4)

Here, *η*(*t*) is white Gaussian noise with zero mean and correlation function 〈*η*(*t*), *η*(*t*′)〉 = *δ*(*t* − *t*′).

The first term is diffusion characterized by a ***diffusion strength** B*^1^, which describes the random displacement of bump center positions due to fluctuations in neuronal firing. For *A*(*φ*) = 0 this term causes diffusive displacement of the center *φ*(*t*) from its initial position *φ*(*t*_0_), with a mean (over realizations) squared displacement of positions 〈[*φ*(*t*) − *φ*(*t*_0_)]^2^〉 = *B* ⋅ (*t* − *t*_0_) that, during an initial phase, increases linearly with time [14, 54, 55], before saturating due to the circular domain of possible center positions [39]. Our theory shows (see *Diffusion* in Materials and methods) that the coefficient *B* can be calculated as a weighted sum over the neuronal firing rates (Fig 1D)

$$\begin{array}{c} B=\sum_{i}(\frac{C_{i}}{S}{)}^{2}(\frac{dJ_{0,i}}{d\varphi }{)}^{2}\phi_{0,i}, \end{array}$$ (5)

where $\frac{dJ_{0,i}}{d\varphi }$ is the change of the input to neuron *i* under shifts of the center position (Fig 1C, orange line), and *S* is a normalizing constant that tends to increase additionally with the synaptic time constant *τ*_*s*_.

The analytical factors *C*_*i*_ express the spatial dependence of the diffusion coefficient on the short-term plasticity parameters through

$$\begin{array}{c} C_{i}=\frac{U(1+2\tau_{u}\phi_{0,i}+U\tau_{u}^{2}\phi_{0,i}^{2})}{(1+U\phi_{0,i}(\tau_{u}+\tau_{x})+U\tau_{u}\tau_{x}\phi_{0,i}^{2}){}^{2}}. \end{array}$$ (6)

^1^In Brownian motion, the *diffusion constant* is usually defined as *D* = *B*/2. The dependence of the single summands in Eq (5) on short-term plasticity parameters is visualized in Fig 1D, where we see that: a) due to the squared spatial derivative $\frac{dJ_{0,i}}{d\varphi }$ of the bump shape and the squared factors *C*_*i*_/*S*, the important contributions to the sum arise primarily from the flanks of the bump; b) for a fixed bump shape, summands increase with stronger short-term depression (larger *τ*_*x*_) and decrease with stronger short-term facilitation (smaller *U*, larger *τ*_*u*_).

The second term in Eq (4) is the ***drift field*** *A*(*φ*), which describes deterministic drifts due to the inclusion of heterogeneities. For heterogeneity caused by variations in neuronal reversal potentials and random network connectivity, we calculate (see *Frozen noise* in Materials and methods) systematic deviations Δ*ϕ*_*i*_(*φ*) of the single neuronal firing rates from the steady-state bump shape that depend on the current position *φ* of the bump center. In *Drift* in Materials and Methods, we show that the drift field is then given by a weighted sum over the firing rate deviations:

$$\begin{array}{c} A\left(\varphi \right)=\sum_{i}\frac{C_{i}}{S}\frac{dJ_{0,i}}{d\varphi }\Delta \phi_{i}\left(\varphi \right), \end{array}$$ (7)

with weighing factors depending on the spatial derivative of the bump shape $\frac{dJ_{0,i}}{d\varphi }$ and the parameters of the synaptic dynamics through the same factors *C*_*i*_/*S*. This is illustrated in Fig 1E: in contrast to Eq (5) summands are now asymmetric with respect to the bump center, since the spatial derivative is not squared.

### Analytical considerations

To calculate the diffusion and drift terms of the last section, we assume the number of neurons *N* to be large enough to treat the center position *φ* as continuous: this allows us (similar to [39]) to derive projection vectors (see *Projection of dynamics onto the attractor manifold* in Materials and methods) that yield the dynamics of the center position. However, the actual projection yields sums over the system size N, whose scaling we made explicit (see *System size scaling* in Materials and methods). For the diffusion strength $\sqrt{B}$ (cf. Eq (5)) we find a scaling as as $1/\sqrt{N}$, in agreement with earlier work [11, 14, 36, 39, 46]. For drift fields caused by random connectivity, we find a scaling with the connectivity parameter *p* and the system size *N* to leading order as $1/(\sqrt{p}N)$, whereas drift fields due to heterogeneity of leak potentials (and other heterogeneous single-neuron parameters) will scale as $1/\sqrt{N}$, both in accordance with earlier results [16, 36, 38, 46].

In addition to reproducing the previously known scaling with the system size *N*, our theory exposes the scaling of both drift and diffusion with the parameters *τ*_*x*_, *τ*_*u*_, and *U* of short-term depression and facilitation via the analytical pre-factors *C*_*i*_/*S* appearing in Eqs (5) and (7). Our result extends the calculation of the diffusion constant [39] to synaptic dynamics with short-term plasticity: In the limiting case of no facilitation and depression (*U* → 1, *τ*_*x*_ → 0ms), the pre-factor reduces to *C*_*i*_ = 1 and the normalization factor simplifies to $S_{\text{static}}=\tau_{s}\sum_{i}(\frac{dJ_{0,i}}{d\varphi }{)}^{2}\phi_{0,i}^{\prime }$, where $\phi_{0,i}^{\prime }=\frac{d\phi_{i}}{dJ_{i}}{|}_{J_{0,i}}$ is the derivative of the firing rate of neuron *i* at its steady-state input *J*_0,*i*_. For static synapses we thereby recover the known result for diffusion [39, Eq. S18], but also add an analogous relation for the drift $A_{\text{static}}\left(\varphi \right)=(\sum_{i}\frac{dJ_{0,i}}{d\varphi }\Delta \phi_{i}\left(\varphi \right))/(\tau_{s}\sum_{i}{\frac{dJ_{0,i}}{d\varphi }}^{2}\phi_{0,i}^{\prime })$. Our approach relies on the existence of a stationary bump state (which is stable for large noise-free homogeneous networks), around which we calculate drift and diffusion as perturbations. Following earlier work [11, 50, 52], we use in our simulations with spiking integrate-and-fire neurons a slow synaptic time constant (*τ*_*s*_ = 100ms) as an approximation of recurrent (NMDA mediated) excitation. While our theory captures the effects of changing this time constant *τ*_*s*_ in the pre-factors *C*_*i*_/*S*, we did not check in simulations whether the bump state remains stable and whether our theory remains valid for very short time constants for *τ*_*s*_.

Finally, two limiting cases are worth highlighting. First, for strong facilitation (*U* → 0) we obtain pre-factors $C_{i}/S=\left(1+2\tau_{u}\phi_{0,i}\right)(\sum_{i}(\frac{dJ_{0,i}}{d\varphi }{)}^{2}\phi_{0,i}^{\prime }[\tau_{s}(1+2\tau_{u}\phi_{0,i})+\tau_{u}^{2}\phi_{0,i}]{)}^{-1}$, indicating that (i) this limit will leave residual drift and diffusion which (ii) will both be controlled by the time constants for facilitation (*τ*_*u*_) and synaptic transmission (*τ*_*s*_), with no dependence upon depression. Second, for vanishing facilitation (*U* → 1 and *τ*_*u*_ → 0) we find that the normalization factor *S* will tend to zero if the depression time constant *τ*_*x*_ is increased to a finite value *τ*_*x*,*c*_. Through the pre-factors *C*_*i*_/*S* this, in turn, yields exploding diffusion and drift terms (see S8 Fig). While this is a general feature of bump systems with short-term depression, the exact value of the critical time constant *τ*_*x*,*c*_ depends on the firing rates and neural implementation of the bump state (see section 6 in S1 Text): for the spiking network investigated here, we find a critical time constant *τ*_*x*,*c*_ = 223.9ms (see S8 Fig). In networks with both facilitation and depression, the critical *τ*_*x*,*c*_ increases as facilitation becomes stronger (see S8 Fig).

### Prediction of continuous attractor dynamics with short-term plasticity

To demonstrate the accuracy of our theory, we chose random connectivity as a first source of frozen variability. Random connectivity was realized in simulations by retaining only a random fraction 0 < *p* ≤ 1 (connection probability) of excitatory-to-excitatory (EE) connections. The uniform connections from and to inhibitory neurons are taken as all-to-all, since the effects of making these random or sparse would have only indirect effects on the dynamics of the bump center positions.

**Fig 2:**
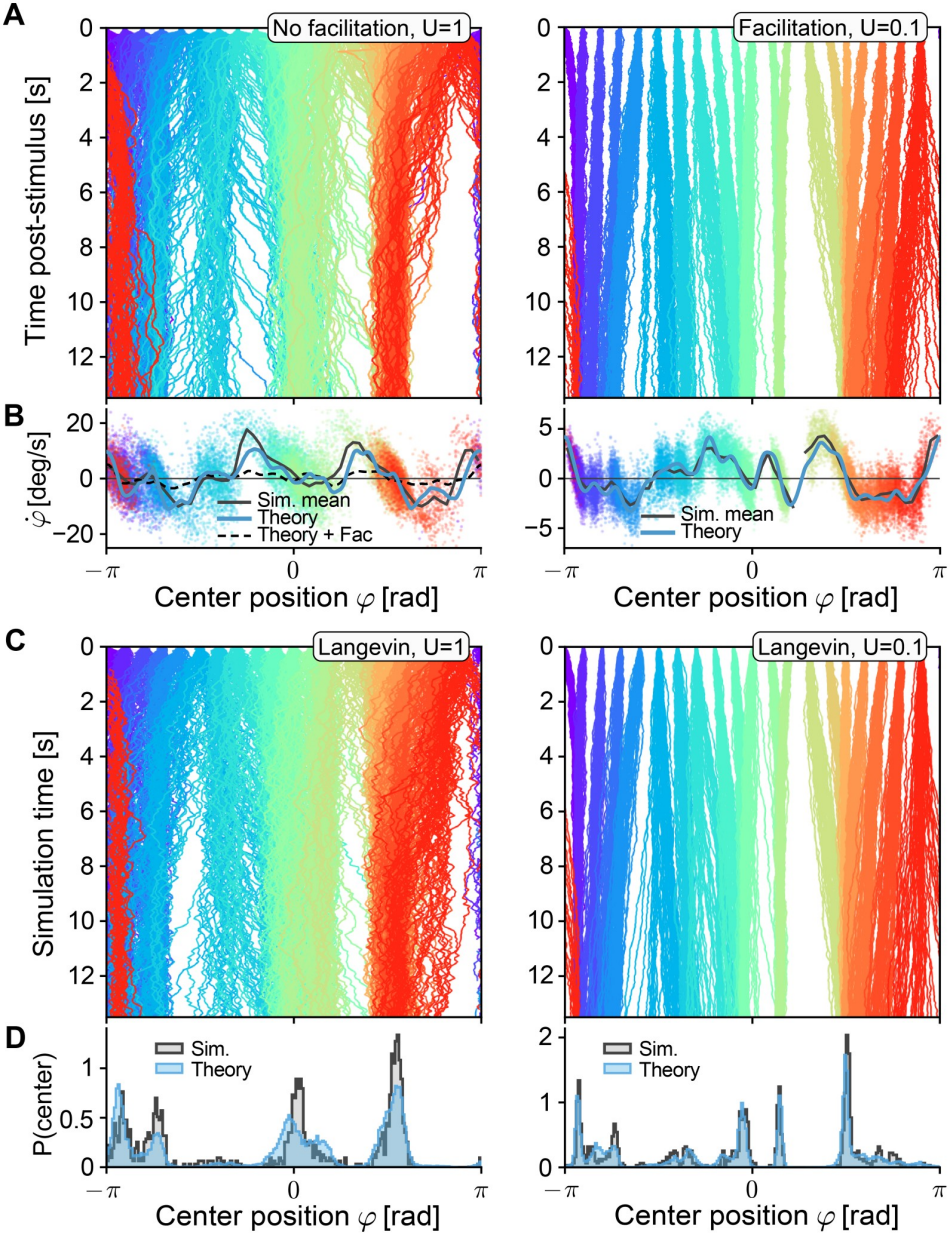
Drift field predictions for varying short-term facilitation. All networks have the same instantiation of random connectivity (*p* = 0.5), similar to Fig 1B1. **A** Centers of excitatory population activity for 50 repetitions of 13.5*s* delay activity, for 20 different positions of initial cues (cue is turned off at *t* = 0) colored by position of the cues. Left: no facilitation (*U* = 1). Right: with facilitation (*U* = 0.1). **B** Drift field as a function of the bump position. The theoretical prediction (blue line, see Eq (7)) of the drift field is compared to velocity estimations along the trajectories shown in A, colored by the line they were estimated from. The thick black line shows the binned mean of data points in 60 bins. For comparison, the predicted drift field for *U* = 0.1 is plotted (thin dashed line). Left: no facilitation (*U* = 1), for comparison the theoretical prediction for the case *U* = 0.01 is plotted as a dashed line. Right: with facilitation (*U* = 0.01). **C** Trajectories under the same conditions as in A, but obtained by forward-integrating the one-dimensional Langevin equation, Eq (4). **D** Normalized histograms of final positions at time *t* = 13.5 for data from spiking simulations (gray areas, data from A) and forward solutions of the Langevin equations (blue areas, data from C). Other STP parameters were: *τ*_*u*_ = 650*ms*, *τ*_*x*_ = 150*ms*.

Our theory accurately predicts the drift-fields *A*(*φ*) (see Eq (7)) induced by frozen variability in networks with short-term plasticity (Fig 2). Briefly, for each neuron 0 ≤ *i* < *N*, we treat each realization of frozen variability as a perturbation Δ_*i*_ around the perfectly symmetric system and use an expansion to first order of the input-output relation *F* to calculate the resulting changes in firing rates (see *Frozen noise* for details):

$$\begin{array}{c} \Delta \phi_{i}\left(\varphi \right)=\frac{dF}{d\Delta_{i}}\Delta_{i}. \end{array}$$ (8)

The resulting terms are then used in Eq (7) to predict the magnitude of the drift field *A*(*φ*) for any center position *φ*, which will, importantly, depend on STP parameters. The same approach can be used to predict drift fields induced by heterogeneous single neuron parameters [36] (see next sections) and additive noise on the E-E connection weights [16, 38].

We first simulated spiking networks with only short-term depression and without facilitation (Fig 2A, left, same network as in Fig 1B1), for one instantiation of random (*p* = 0.5) connectivity. Numerical estimates of the drift in spiking simulations (by measuring the displacement of bumps over time as a function of their position, see *Spiking simulations* in Materials and methods for details) yielded drift-fields in good agreement with the theoretical prediction (Fig 2B, left). At points where the drift field prediction crosses from positive to negative values (e.g. Fig 2B, left, $\varphi =\frac{\pi }{2}$), we expect stable fixed points of the center position dynamics in agreement with simulation results, which show trajectories converging to these points. Similarly, unstable fixed points (negative-to-positive crossings) can be seen to lead to a separation of trajectories (e.g. Fig 2A, left, $\varphi =-\frac{\pi }{2}$). In regions where the positional drifts are predicted to lie close to zero (e.g. Fig 2A, left *φ* = 0) the effects of diffusive dynamics are more pronounced. Finally, numerical integration of the full 1-dimensional Langevin equation Eq (4) with coefficients predicted by Eqs (5)–(7), produces trajectories with dynamics very similar to the full spiking network (Fig 2C, left). When comparing the center positions after 13.5*s* of delay activity between the full spiking simulation and the simple 1-dimensional Langevin system, we found very similar distributions of final positions (Fig 2D, left, compare to Fig 1B1, histogram). Our theory thus produces an accurate approximation of the dynamics of center positions in networks of spiking neurons with STP, thereby reducing the complex dynamics of the whole network to a simple equation. It should be noted that, in regions with strong drift or steep negative-to-positive crossings, the numerically estimated drift-fields deviate from the theory due to under-sampling of these regions as trajectories move quickly through them, yielding fewer data points. In *Short-term plasticity controls drift* we additionally show that the theory, as it relies on a linear expansion of the effects of heterogeneities on the neuronal firing rates, tends to generally over-predict drift-fields as heterogeneities become stronger.

Introducing strong short-term facilitation (*U* = 0.1) reduces the predicted drift fields (Fig 2B, left, dashed line), which resemble a scaled-down version of the drift-field for the unfacilitated case. We confirmed this theoretical prediction by simulations including facilitation (Fig 2A, right): the resulting drift fields show significant reduction of speeds (Fig 2B, right) while zero crossings remained similar to the unfacilitated network, similar to the results in [38]. Theoretical predictions of the drift fields with bump shapes extracted from these simulations again show an accurate prediction of the dynamics (Fig 2B, right). Thus, as before, forward integrating the simple 1-dimensional Langevin-dynamics yields trajectories (Fig 2C, right) highly similar to those of the full spiking network, with closely matching distributions of final positions (Fig 2D, right), indicative of a matching strength of diffusion. In summary, our theory predicts the effects of STP on the joint dynamics of diffusion and drift due to network heterogeneities, which we will show in detail in the next sections.

### Short-term plasticity controls diffusion

**Fig 3:**
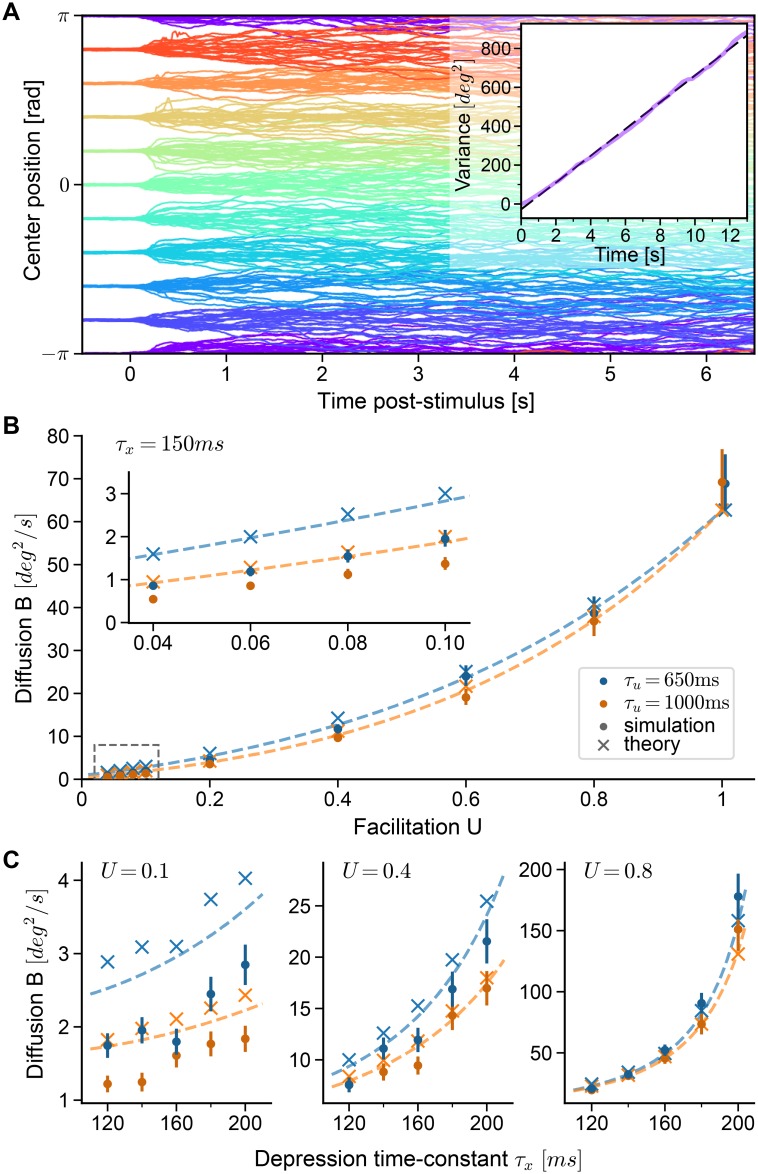
Diffusion on continuous attractors is controlled by short-term plasticity. **A** Center positions of 20 repeated simulations of the reference network (*U* = 1, *τ*_*x*_ = 150ms) for 10 different initial cue positions each. Inset: Estimated variance of deviations of center positions *φ*(*t*) from their positions *φ*(0.5) at *t* = 0.5*s* (purple) as a function of time (〈[*φ*(*t*) − *φ*(0.5)]^2^〉), together with linear fit (dashed line). The slope of the dashed line yields an estimate of *B* (Eq (5)). **B,C** Diffusion strengths estimated from simulations (dots, error bars show 95% confidence interval, estimated by bootstrapping) compared to theory. Dashed lines show theoretical prediction using firing rates measured from the reference network (*U* = 1, *τ*_*x*_ = 150*ms*), while crosses are theoretical estimates using firing rates measured for each set of STP parameters separately (crosses). **B** Diffusion strength as a function of facilitation parameter *U*. Inset shows zoom of region indicated in the dashed area in the lower left. Increasing the facilitation time constant *τ*_*u*_ = 650*ms* (blue) to *τ*_*u*_ = 1*s* (orange) affects diffusion only slightly. In panels A and B, the depression time constant is *τ*_*x*_ = 150*ms*. **C** Diffusion strength as a function of depression time constant *τ*_*x*_. Results for three different values of *U* are shown (note the change in scale). Colors indicate the two different values for the facilitation time constant also used in panel B.

To isolate the effects of STP on diffusion, we simulated networks *without frozen noise* for various STP parameters. For each combination of parameters, we simulated 1000 repetitions of 13.5*s* delay activity (after cue offset) distributed across 20 uniformly spaced initial cue positions (see Fig 3A for an example). From these simulations, the strength of diffusion was estimated by measuring the growth of variance (over repetitions) of the distance of the center position from its initial position as a function of time (see *Spiking simulations* in Materials and methods for details). For all parameters considered, this growth was well fit by a linear function (e.g. Fig 3A, inset), the slope of which we compared to the theoretical prediction obtained from the diffusion strength *B* (Eq (5)).

We find that facilitation and depression control the amount of diffusion along the attractor manifold in an antagonistic fashion (Fig 3B and 3C). First, increasing facilitation by lowering the facilitation parameter *U* from its baseline *U* = 1 (no facilitation) towards *U* = 0, while keeping the depression time constant *τ*_*x*_ = 150*ms* fixed, decreases the measured diffusion strength over an order of magnitude (Fig 3B, dots). On the other hand, increasing the facilitation time constant *τ*_*u*_ from *τ*_*u*_ = 650*ms* to *τ*_*u*_ = 1000*ms* (Fig 3B, orange and blue dots, respectively) only slightly reduces diffusion. Our theory further predicts that increasing the facilitation time constants above *τ*_*u*_ = 1*s* will not lead to large reductions in the magnitude of diffusion (see S2 Fig). Second, we find that increasing the depression time constant *τ*_*x*_ for fixed *U*, thereby slowing down recovery from depression, leads to an increase of the measured diffusion (Fig 3C). More precisely, increasing the depression time constant from *τ*_*x*_ = 120*ms* to *τ*_*x*_ = 200*ms* leads only to slight increases in diffusion for strong facilitation (*U* = 0.1), but to a much larger increase for weak facilitation (*U* = 0.8).

For a comparison of these simulations with our theory, we used two different approaches. First, we estimated the diffusion strength by using the precise shape of the stable firing rate profile extracted separately for each network with different sets of parameters. This first comparison with simulations confirms that the theory closely describes the dependence of diffusion on short-term plasticity for each parameter set (Fig 3B, crosses). The observed effects could arise directly from changes in STP parameters for a fixed bump shape, or indirectly since STP parameters also influence the shape of the bump. To separate such direct and indirect effects, we used for a second comparison a theory with fixed bump shape, i.e. the bump shape measured in a “reference network” (*U* = 1, *τ*_*x*_ = 150*ms*) and extrapolated curves by changing only STP parameters in Eq (5). This leads to very similar predictions (Fig 3B, dashed lines) and supports the following conclusions: a) the diffusion to be expected in attractor networks with similar observable quantities (mainly, the bump shape) depends only on the short-term plasticity parameters; b) the bump shapes in the family of networks we have investigated are sufficiently similar to be approximated by measurement in a single reference network. It should be noted that the theory tends to slightly over-estimate the amount of diffusion, especially for small facilitation *U* (see Fig 3B and 3C left). This may be because slower bump movement decreases the firing irregularity of flank neurons, which deviates from the Poisson firing assumption of our theory (see Discussion). However, given the simplifying assumptions needed to derive the theory, the match to the spiking network is surprisingly accurate.

### Short-term plasticity controls drift

Having established that our theory is able to predict the effect of STP on diffusion, as well as drift for a single instantiation of random connectivity, we wondered how different sources of heterogeneity (frozen noise) would influence the drift of the bump. We considered two sources of heterogeneity: First, random connectivity as introduced above, and second, heterogeneity of the leak reversal potential parameters of excitatory neurons: leak reversal potentials of excitatory neurons are given by *V*_*L*_ + Δ_*L*_, where Δ_*L*_ is normally distributed with zero mean and standard deviation *σ*_*L*_ [36]. The resulting fields can be calculated by calculating the resulting perturbations to the firing rates of neurons by Eq (8) (see *Frozen noise* in Materials and methods for details).

The theory developed so far allowed us to predict drift-fields for a given realization of frozen noise, controlled by the noise parameters *p* (for random connectivity) and *σ*_*L*_ (for heterogeneous leak reversal-potentials) (see S3 Fig for a comparison of predicted drift fields to those measured in simulations for varying STP parameters and varying strengths of frozen noises). We wondered, whether we could take the level of abstraction of our theory one step further, by predicting the magnitude of drift fields from the frozen noise parameters only, independently of a specific realization. First, the expectation of drift fields under the distributions of the frozen noises vanishes for any given position: 〈*A*(*φ*)〉_frozen_ = 0, where the expectation 〈.〉_frozen_ is taken over both noise parameters. We thus turned to the expected squared magnitude of drift fields under the distributions of these parameters (see *Squared field magnitude* in Materials and methods for the derivation):

$${\langle A^{2}\rangle }_{\text{frozen}}=\frac{1}{S^{2}}\underset{i}{\sum }C_{i}^{2}\left(\frac{{\left(\phi_{0,i}^{\prime }\right)}^{2}}{N_{E}^{2}}\left(\frac{1}{p}-1\right)\underset{j}{\sum }{\left(s_{0,j}\right)}^{2}{\left(w_{ij}^{\text{EE}}\right)}^{2}+{\left(\frac{d\phi_{0,i}}{d\Delta_{i}^{L}}\right)}^{2}\sigma_{L}^{2}\right),$$ (9)

where *s*_0,*j*_ is the steady-state synaptic activation. Here, we introduced the derivatives of the input-output relation with respect to the noise sources that appear in Eq (8): $\phi_{0,i}^{\prime }=\frac{dF}{dJ}\left(J_{0,i}\left(\varphi \right)\right)$ is the derivative with respect to the steady state synaptic input, and $\frac{d\phi_{0,i}}{d\Delta_{i}^{L}}$ is the derivative with respect to the perturbation in the leak potential. In *Squared field magnitude* in Materials and Methods, we show that Eq (9) is independent of the center position *φ*, and can be estimated from simulations as the variance of the drift field across positions, averaged over an ensemble of network instantiations.

We defined the root of the expected squared magnitude of Eq (9) as the *expected field magnitude*:

$$\begin{array}{c} \sqrt{{\left\langle A^{2}\right\rangle }_{\text{frozen}}}. \end{array}$$ (10)

This quantity predicts the magnitude of the deviations of drift-fields from zero that are expected from the parameters that control the frozen noise—in analogy to the standard deviation for random variables, it predicts the standard deviation of the fields. To compare this quantity to simulations, we varied both heterogeneity parameters. First, the connectivity parameter *p* was varied between 0.25 and 1. Second, for heterogeneities in leak reversal-potentials, we chose values for the standard deviation *σ*_*L*_ of leak-reversal potentials between 0*mV* and 1.5*mV*, which lead to a similar range of drift magnitudes as those of randomly connected networks. For each combination of heterogeneities and STP parameters (networks had either random connections or heterogeneous leaks) we then realized 18–20 networks, for which we simulated 400 repetitions of 6.5*s* of delay activity each (20 uniformly spaced positions of the initial cue). We then estimated the drift-field numerically by recording displacements of bump centers along their trajectories (as in Fig 2A and 2B) and measured the standard deviation of the resulting fields across all positions.

**Fig 4:**
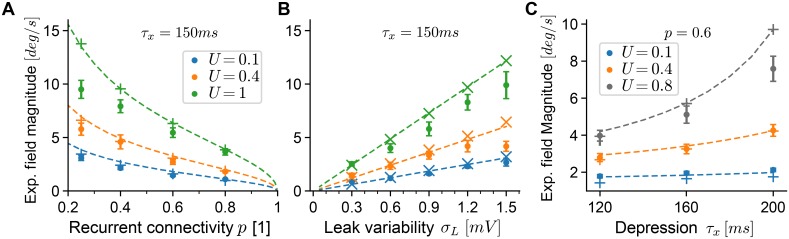
Drift field magnitude is controlled by short-term plasticity. **A** Expected magnitude of drift fields as a function of the sparsity parameter *p* of recurrent excitatory-to-excitatory connections. Dots are the standard deviation of fields estimated from 400 trajectories (see main text) of each network, averaged over 18–20 realizations for each noise parameter and facilitation setting (error bars show 95% confidence of the mean). Theoretical predictions (dashed lines) are given by Eq (10) extrapolated from the reference network (*U* = 1, *τ*_*x*_ = 150). For validation, we also estimated Eq (10) with coefficients measured from each simulated network separately (plus signs). The depression time constant was *τ*_*x*_ = 150ms. **B** Same as in panel A, with heterogeneous leak-reversal potentials as the source of frozen noise. Validation predictions are plotted as crosses. **C** Same as in panels A,B but varying the depression time constant *τ*_*x*_ for a fixed level of frozen noise (random connectivity, *p* = 0.6). In all panels, the facilitation time constant was *τ*_*u*_ = 650ms.

Similar to the analysis of diffusion above, we find that facilitation and depression elicit antagonistic control over the magnitude of drift fields. In both simulations and theory, we find (Fig 4A and 4B) that the expected field magnitude *decreases* as the effect of facilitation is *increased* from unfacilitated networks (*U* = 1) through intermediate levels of facilitation (*U* = 0.4) to strongly facilitating networks (*U* = 0.1). Our theory predicts this effect surprisingly well, which we validated twofold (as for the diffusion magnitude). First, we used Eq (10) with all parameters and coefficients estimated from each spiking simulation separately (Fig 4A and 4B, plus-signs and crosses). Second, we extrapolated the theoretical prediction by using coefficients in Eq (9) from the unfacilitated reference network only (*U* = 1, *τ*_*x*_ = 150*ms*) but changed the facilitation and heterogeneity parameters (Fig 4A and 4B, dashed lines). The largest differences between the extrapolated and full theory are seen for *U* < 1 and randomly connected networks (*p* < 1), which we found to result from the fact that bump shapes for these networks tended to be slightly reduced under random and sparse connectivity (e.g. the top firing rate is reduced to ∼ 35*Hz* for *U* = 0.1, *p* = 0.25). Generally, as noise levels increase, our theory tends to over-estimate the squared magnitude of fields, since we rely on a linear expansion of perturbations to the firing rates to calculate fields (Eq (8)). Such deviations are expected as the magnitude of firing rate perturbations increases, and could be counter-acted by including higher-order terms. Since in the theory facilitation (and depression) only scales the firing rate perturbations (Eq (7)), these deviations can also be observed across facilitation parameters. Finally, we performed a similar analysis to investigate the effect of short-term depression on drift fields. Here, we varied the depression time constant *τ*_*x*_ for randomly connected networks with *p* = 0.6, by simulating networks with combinations of short-term plasticity parameters from *U* ∈ {0.1, 0.4, 0.8} and *τ*_*x*_ ∈ {120*ms*, 160*ms*, 200*ms*} (Fig 4C). We find that an increase of the depression time constant leads to increased magnitude of drift fields, which again is well predicted by our theory.

### Short-term plasticity controls memory retention

**Fig 5:**
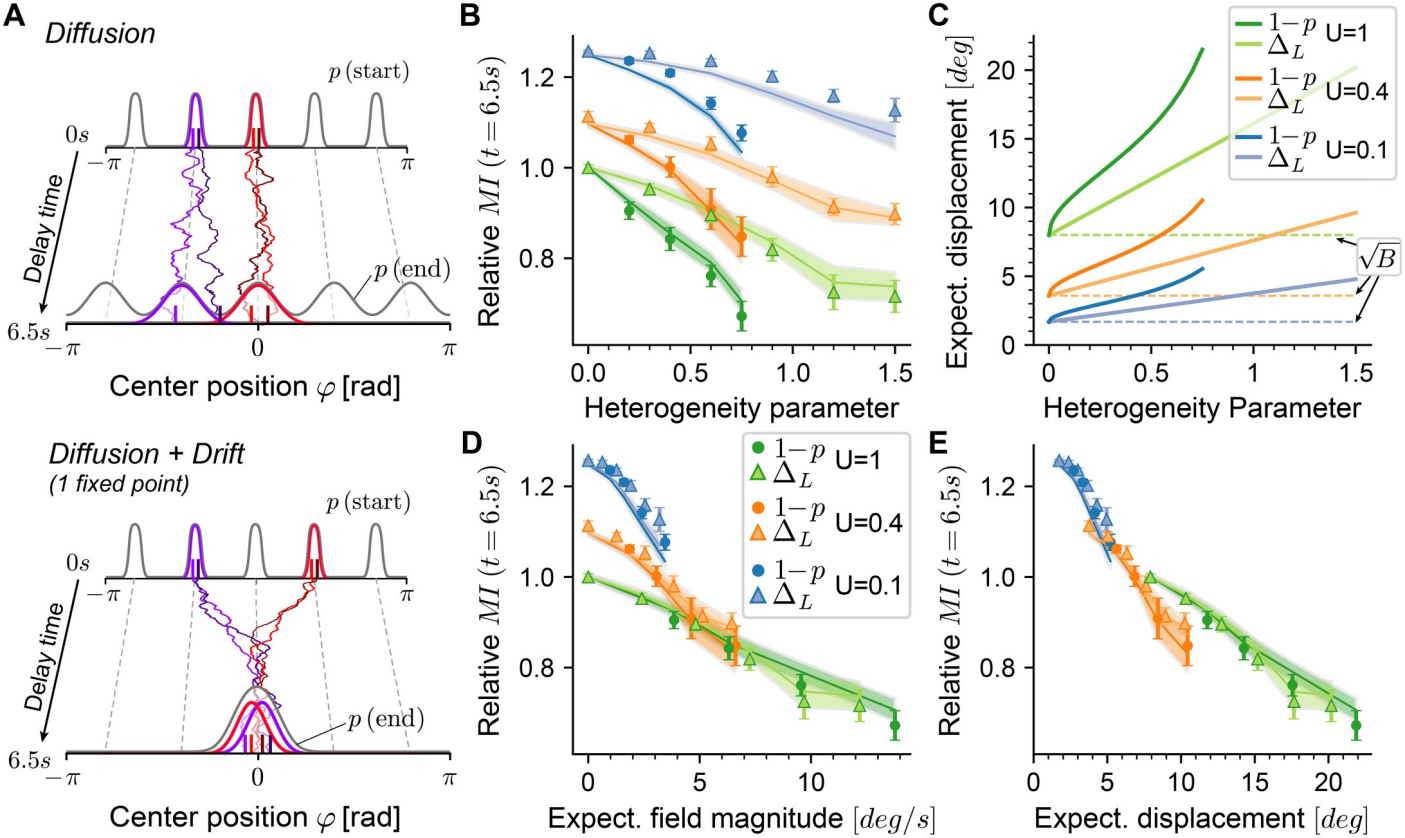
Short-term facilitation increases memory retention. **A** Illustration of the effects of diffusion (top) and additional drift (bottom) on the temporal evolution of distributions of initial positions *p*(start) towards distributions of final positions *p*(end) over 6.5*s* of delay activity. The bump is always represented by its center position *φ*. Two peaks in the distribution of initial positions *φ*(0) and their corresponding final positions *φ*(6.5) are highlighted by colors (purple, red), together with example trajectories of the center positions. Top: Diffusion symmetrically widens the initial distribution. Bottom: Strong drift towards one single fixed point of bump centers (*φ* = 0) makes the origin of trajectories indistinguishable. **B** Normalized mutual information (MI, see text for details) of distributions of initial and final bump center positions in working memory networks for different STP parameters and heterogeneity parameters(blue: strong facilitation, see legend in panel D). Dots and triangles are average MI (18–20 realizations, error bars show 95% CI) obtained from spiking network simulations. Lines show average MI calculated from Langevin dynamics for the same networks, repetitions and realizations (see text, shaded area shows 95% CI). Heterogeneity parameters are *σ*_*L*_ (triangles, in units of *mV*) and 1 − *p* (circles), where *p* is the connection probability. **C** Expected displacement |Δ*φ*|(1*s*) for the same networks as in panel B. Dashed lines indicate displacement induced by diffusion only ($\sqrt{B}$), solid lines show the total displacement (including displacement due to drift, calculated as the expected field magnitude $\sqrt{{\langle A^{2}\rangle }_{\text{frozen}}}$). **D** Same as panel B, with x-axis showing the expected field magnitude. **E** Same as panel B, with x-axis showing the expected displacement. In panels B-D, all STP parameters except *U* were kept constant at *τ*_*u*_ = 650ms, *τ*_*x*_ = 150ms.

The theory developed in previous sections shows that diffusion and drift of the bump center *φ* are controlled antagonistically by short-term depression and facilitation. In a working memory setup, we can view the attractor dynamics as a noisy communication channel [56] that maps a set of initial positions *φ*(*t* = 0*s*) (time of the cue offset in the attractor network) to associated final positions *φ*(*t* = 6.5*s*), after a memory retention delay of 6.5*s*. We used the distributions of initial and (associated) final positions to investigate the combined impact of diffusion and drift on the retention of memories (Fig 5A). Because of diffusion, distributions of positions will widen over time, which degrades the ability to distinguish different initial positions of the bump center (Fig 5A, top). Additionally, directed drift of the dynamics will contract distributions of different initial positions around the same fixed points, making them essentially indistinguishable when read out (Fig 5A, bottom).

As a numerical measure of this ability of such systems to retain memories over the delay period, we turned to mutual information (MI), which provides a measure of the amount of information contained in the readout position about the initially encoded position [57, 58]. To measure MI from simulations (see *Mutual information measure* in Materials and methods), we analyzed network simulations for varying short-term facilitation parameters (*U*) and magnitudes of frozen noises (*p* and *σ*_*L*_) (same data set as Fig 4A and 4B). We recorded the center positions encoded in the network at the time of cue-offset (*t* = 0) and after 6.5*s* of delay activity, and used binned histograms (100 bins) to calculate discrete probability distributions of initial (*t* = 0) and final positions (*t* = 6.5). For each trajectory simulated in networks of spiking integrate-and-fire neurons, we then generated a trajectory starting at the same initial position by using the Langevin equation Eq (4) that describes the drift and diffusion dynamics of center positions. The MI calculated from the resulting distributions of final positions (again at *t* = 6.5) for each network serve as the theoretical prediction for each network. As a reference, we used the spiking network without facilitation (*U* = 1, *τ*_*u*_ = 650*ms*, *τ*_*x*_ = 150*ms*) and no frozen noises (*p* = 1, *σ*_*L*_ = 0*mV*) and normalized the MI of all other networks (both for spiking simulations and theoretical predictions) with respect to the reference, yielding the measure of *relative MI* presented in Fig 5B–5E.

We found that the relative MI decreased compared to the reference network as network heterogeneities were introduced (Fig 5B, green). This was expected, since directed drift caused by heterogeneities leads to a loss of information about initial positions. There were two effects of increased short-term facilitation (by decreasing the parameter *U*). First, diffusion was reduced, which was visible in a vertical shift of the relative MI for facilitated networks (Fig 5A, orange and blue, at 0 heterogeneity). Second, the effects of frozen noise decreased with increasing facilitation, which was visible in the slopes of the MI decrease (see also S4 Fig). The MI obtained by integration of the Langevin equations (see above) matched those of the simulations well (Fig 5A, lines). From earlier results, we expected the drift-fields to be slightly over-estimated by the theory as the heterogeneity parameters increase (Fig 4), which would lead to an under-estimation of MI. We did observe this here, although for *U* = 1 the effect was slightly counter-balanced by the under-estimated level of diffusion (cf. Fig 3A, right), which we expected to increase the MI. For networks with stronger facilitation (*U* = 0.1), we systematically over-estimated diffusion (cf. Fig 3, left), and therefore under-estimated MI.

Using our theory, we were able to simplify the functional dependence between MI, short-term plasticity, and frozen noise. Combining the effects of both diffusion and drift into a single quantity for each network, we replaced the field *A*(*φ*) by our theoretical prediction $\sqrt{{\langle A^{2}\rangle }_{\text{frozen}}}$ in Eq (4) and forward integrated the differential equation for a time interval Δ*t* = 1*s*, to arrive at the *expected displacement* in 1*s*:

$$\begin{array}{c} |\Delta \varphi |\left(1s\right)=\sqrt{{\left\langle A^{2}\right\rangle }_{\text{frozen}}}\cdot 1s+\sqrt{B\cdot 1s}. \end{array}$$ (11)

This quantity describes the expected absolute value of displacement of center positions during 1*s*: it increases as a function of the frozen noise distribution parameters (Fig 5C), but even in the absence of frozen noise it is nonzero due to diffusion. Plotting the MI data in dependence of the first term only ($\sqrt{{\langle A^{2}\rangle }_{\text{frozen}}}$), shows that the MI curves collapse onto a single curve for each facilitation parameter (Fig 5D). Finally, plotting the MI data against |Δ*φ*|(1*s*) we find that all data collapse on to nearly a single curve (Fig 5E). Thus, the effects of the two sources of frozen noise (corresponding to 〈*A*^2^〉_frozen_) and diffusion (corresponding to *B*) are unified into a single quantity |Δ*φ*|(1*s*).

**Fig 6:**
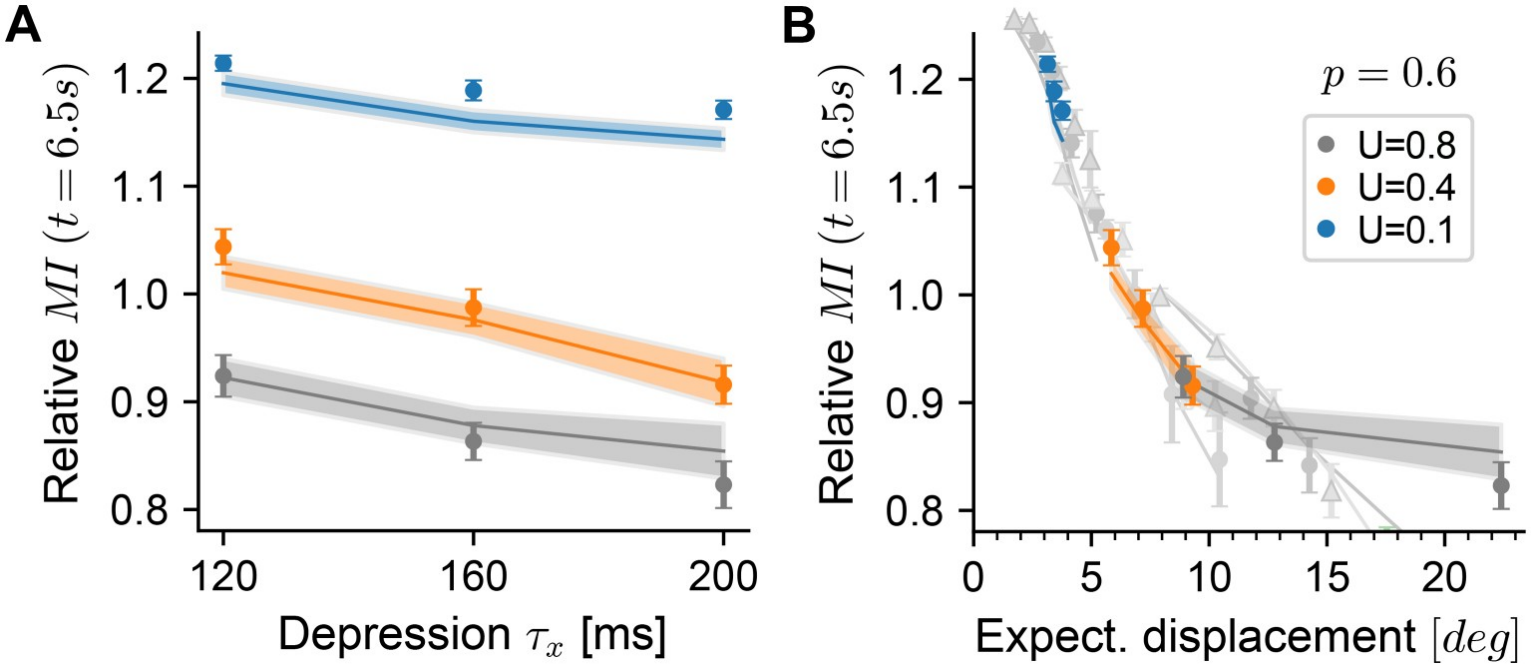
Short-term depression decreases memory retention. **A** Same as Fig 5B, for network simulations with varying *τ*_*x*_ and *U* (see legend in panel B). MI is normalized to the same value as there. **B** Same as panel A, with x-axis showing the expected displacement. Gray data points and lines are the data plotted in Fig 5E. The facilitation time constant was kept constant at *τ*_*u*_ = 650ms.

We performed the same analyses on a large set of network simulations with fixed random connectivity (*p* = 0.6) and varying STP parameters for both depression (*τ*_*x*_) and facilitation (*U*) (same data set as in Fig 4C). Increasing the short-term depression time constant *τ*_*x*_ leads to decreased relative MI with a positive offset induced through stronger facilitation (Fig 6A, blue line). Calculating the expected displacement for these network configurations collapsed the data points mostly onto the same curve as earlier (Fig 6B). For strong depression combined with weak facilitation (*τ*_*x*_ = 200*ms*, *U* = 0.8), the drop-off of the relative MI saturates earlier, indicating that for these strongly diffusive networks the effect on MI may not be sufficiently captured by its relationship to |Δ*φ*|(1*s*).

### Linking theory to experiments: Distractors and network size

The abstraction of our theory condenses the complex dynamics of bump attractors in spiking integrate-and-fire networks into a high-level description of a few macroscopic features, which in turn allows matching the theory to behavioral experiments. Here, we demonstrate how such quantitative links could be established using two different features: 1) the sensitivity of the working memory circuit to distractors, and 2) the stability of working memory expressed by the expected displacement. We stress that our model is a simplified description of biological circuits, in which several further sources of variability and also dynamical processes influencing displacement should be expected (see Discussion). Thus, at the current level of simplification, the results presented in this section should be seen as proofs of principle rather than quantitative predictions for a cortical setting.

#### Predicting the sensitivity to distractor inputs

**Fig 7:**
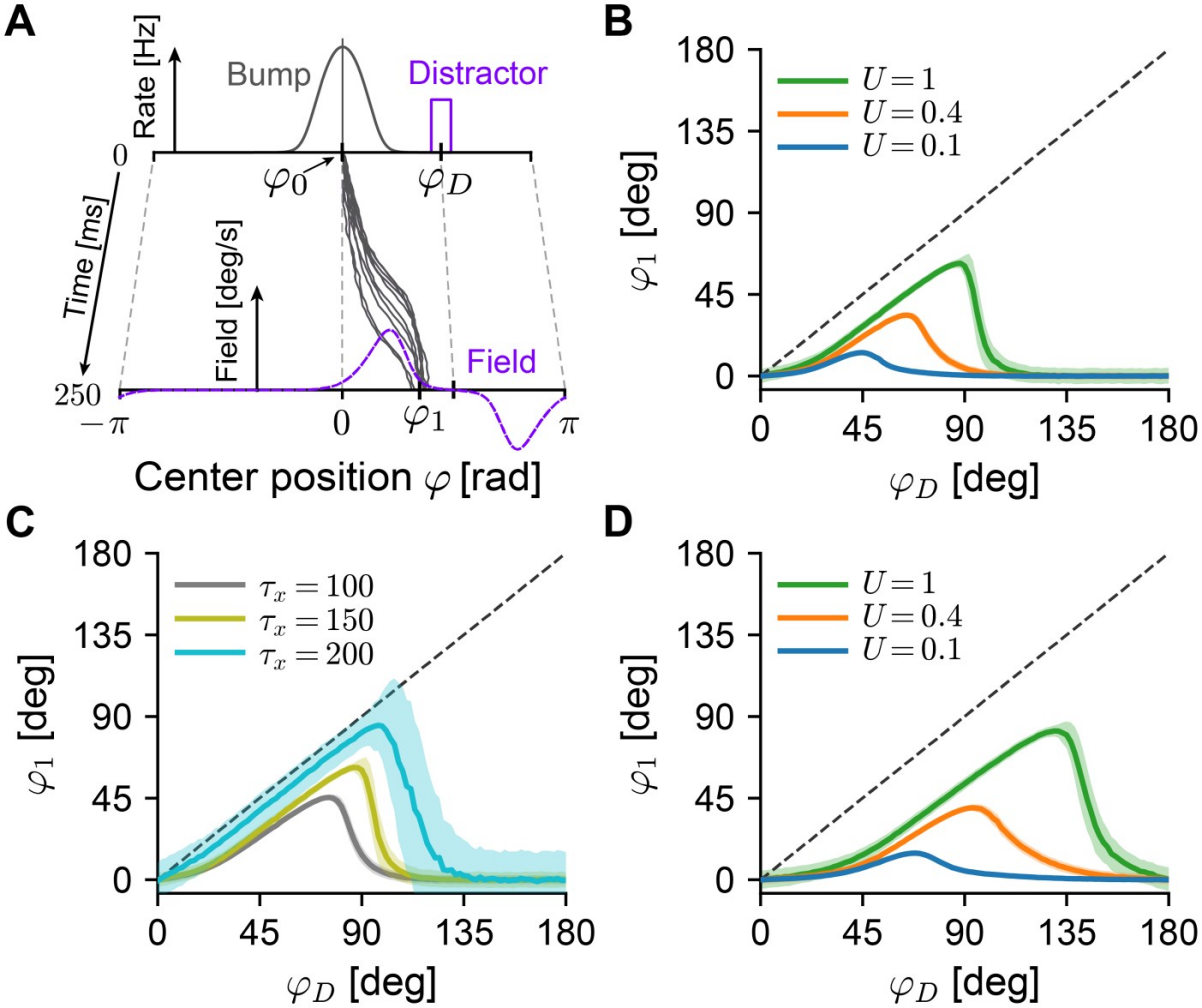
Effect of short-term plasticity on distractor inputs. **A** While a bump (“Bump”) is centered at an initial angle *φ*_0_ (chosen to be 0), additional external input causes neurons centered around the position *φ*_*D*_ to fire at elevated rates (“Distractor”). The theory predicts the shape and magnitude of the induced drift field (“Field”) and the mean bump center *φ*_1_ after 250ms of distractor input. Gray trajectories are example simulations of bump centers of the corresponding Langevin equation Eq (4). **B** Mean final positions *φ*_1_ of bump centers (1000 repetitions, shaded areas show 1 standard deviation) as a function of the distractor input location *φ*_*D*_. Increased short-term facilitation (blue: strong facilitation, *U* = 0.1; orange: intermediate facilitation, *U* = 0.4; green: no facilitation *U* = 1) leads to less displacement due to the distractor input. Other STP parameters were kept constant at *τ*_*u*_ = 650ms, *τ*_*x*_ = 150ms. **C** Same as panel B, for three different depression time constants *τ*_*x*_, while keeping *U* = 0.8, *τ*_*u*_ = 650ms fixed. **D** Same as panel B, with a broader bump half-width (*σ*_*g*_ = 0.8rad ≈ 45.8 deg). All other panels use the same bump half-width as in the rest of the study (*σ*_*g*_ = 0.5rad ≈ 28.7 deg) (see S1 Fig).

In a biological setting, drifts introduced by network heterogeneities (frozen noise) could be significantly reduced by (long-term) plasticity [36]. To measure the intrinsic stability of continuous attractor models, earlier studies [11, 47, 59] have proposed to use *distractor inputs* (Fig 7A): providing a short external input centered around a position *φ*_*D*_ to the network, the center position of an existing bump state will be biased towards the distracting input, with stronger biases appearing for closer distractors. In the context of our theory, we consider a weak distractor as an additional heterogeneity that induces drift. Therefore the time scale of bump drift caused by distractor-induced heterogeneity enables us link our theory to behavioral experiments [59].

Our theory can readily yield quantitative predictions for the distractor paradigm. To accommodate distractor inputs in the theory, we assume that they cause some units *i* to fire at elevated rates *ϕ*_0,*i*_ + Δ*ϕ*_*i*_, which will introduce a drift field according to Eq (7) (Fig 7A, purple dashed line). The resulting dynamics (Eq (4)) of diffusion and drift during the presentation of the distractor input then allow us to calculate the expected shift of center positions as a function of all network parameters, including those of short-term plasticity. Repeating this paradigm for varying positions of the distractor inputs (see *Distractor analysis* in Materials and methods for details), our theory predicts that strong facilitation will strongly decrease both the effect and radial reach of distractor inputs (Fig 7B, blue), when compared to the unfacilitated system (Fig 7B, green)—in qualitative agreement with simulation results involving a related (cell-intrinsic) stabilization mechanism [47]. Conversely, we predict that longer recovery from short-term depression tends to increase the sensitivity to distractors (Fig 7C). The total displacement caused by a distractor input is found by integrating the resulting dynamics of Eq (4) over the stimulus duration. As such, the magnitude of the displacement will increase both with the amplitude and the duration of the distractor input. Finally, our theory demonstrates that the bump shape, in particular the width of the bump, influence the radial reach of distractor inputs (Fig 7D).

#### Relating displacement to network size in working memory networks

The simple theoretical measure of expected displacement |Δ*φ*|(1*s*) introduced in the last section can be related to behavioral experiments: a value of |Δ*φ*|(1*s*) = 1.0 deg lies in the upper range of experimentally reported deviations due to diffusive and systematic errors in behavioral studies [60, 61]. What are the microscopic circuit compositions that can attain such a (high) level of working memory stability? In particular, since an increase in network size can reduce diffusion [11] and the effects of random heterogeneities [16, 36, 38, 46], we turned to the question: *which networks size would be needed to yield this level of stability in a one-dimensional continuous memory system*?

To address the question of network size, we extended our theory to include the size *N* of the excitatory population as an explicit parameter (see *System size scaling* in Materials and methods for details). Using numerical coefficients in Eq (4) extracted from the spiking simulation of a reference network (*U* = 1, *τ*_*x*_ = 150 and *N*_*E*_ = 800), we extrapolated the theory by changing the system size *N* and short-term plasticity parameters. We then constrained parameters of our theory by published data (Table 1). Short-term plasticity parameters were based on two groups of strongly facilitating synapses found in a study of mammalian (ferret) prefrontal cortex [62]. The same study reported a general probability *p* = 0.12 of pyramidal cells to be connected. However, for pairs of pyramidal cells that were connected by facilitating synapses, the study found a high probability of reciprocal connections (*p*_*rec*_ = 0.44): thus if neuron A was connected to neuron B (with probability *p*), neuron B was connected to neuron A with high probability (*p*_*rec*_), resulting in a non-random connectivity. To approximate this in the random connectivities supported by our theory, we evaluated a second, slightly elevated, level of random connectivity, that has the same mean connection probability as the non-random connectivity with these additional reciprocal connections: *p* + *p* ⋅ *p*_*rec*_ = 0.1728. For the standard deviation of leak reversal-potentials *σ*_*L*_, we used values measured in two studies [63, 64].

**Table 1:** Upper bounds on system-sizes for stable continuous attractor memory in prefrontal cortex.

STP parameters	Δ*φ*(1*s*)	*p*	*σ*_*L*_	Network size *N*
*U* = 0.17 *τ*_*u*_ = 563*ms* *τ*_*x*_ = 242*ms* [62, E1b]	1.0 deg [60, 61]	0.12 [62]	1.7mV [63, RS]	79 504
			2.4mV [64, fa-RS]	127 465
		0.1728 [62]	1.7mV	79 047
			2.4mV	127 205
	0.5 deg [60]	0.12	2.4mV	507 607
*U* = 0.35 *τ*_*u*_ = 482*ms* *τ*_*x*_ = 163*ms* [62, E1a]	1.0 deg	0.12	1.7mV	102 292
			2.4mV	163 896
		0.1728	1.7mV	101 836
			2.4mV	163 638
	0.5 deg	0.12	2.4mV	653 350

Theoretical predictions of Eq (11) optimized for the number of excitatory neurons *N* that are needed to achieve a given level of expected displacement |Δ*φ*|(1*s*) under given parameters of short-term plasticity and frozen noises. RS: regular spiking pyramidal cells, fa-RS: fast-adapting regular spiking pyramidal cells.

The resulting theory makes quantitative predictions for combinations of network size *N* and all other parameters that yield the desired levels of working memory stability (Table 1, see also S5 Fig). Network sizes were all smaller than 10^6^ neurons, with values depending most strongly on the value of the facilitation parameter *U* and the magnitude of the leak reversal-potential heterogeneities *σ*_*L*_. Since the expected field magnitude scales weakly ($1/\sqrt{p}$) with the recurrent connectivity *p*, increasing *p* lead only to comparatively small decreases in the predicted network sizes. Finally, we see that the increasing the reliability of networks comes at a high cost: decreasing the expected displacement to |Δ*φ*|(1*s*) = 0.5 deg [60] increases the required number of neurons by nearly a number of 4 for both facilitation settings we investigated. Nevertheless, these network sizes still lie within anatomically reasonable ranges [65].

In summary, we have provided a proof of principle, that the high-level description of our theory can be used to predict network sizes, by exposing features that can be constrained by experimental measurements. Given the simplifying assumptions of our models and the sources of variability that we could include at this stage, continuous attractor networks with realistic values for the strength of facilitation and depression of recurrent connections could achieve sufficient stability, even in the presence of biological variability.

## Discussion

We presented a theory of drift and diffusion in continuous working memory models, exemplified on a one-dimensional ring attractor model. Our framework generalizes earlier approaches calculating the effects of fast noise by projection onto the attractor manifold [37, 39, 40] by including the effects of short-term plasticity (see [45] for a similar analysis for facilitation only). Our approach further extends earlier work on drift in continuous attractors with short-term plasticity [38] to include diffusion and the dynamics of short-term depression. Our theory predicts that facilitation makes continuous attractors robust against the influences of both dynamic noise (introduced by spiking variability) and frozen noise (introduced by biological variability) whereas depression has the opposite effect. We use this theory to provide, together with simulations, a novel quantitative analysis of the interaction of facilitation and depression with dynamic and frozen noise. We have confirmed the quantitative predictions of our theory in simulations of a ring-attractor implemented in a network model of spiking integrate-and-fire neurons with synaptic facilitation and depression, and found theory and simulation to be in good quantitative agreement.

In Section *Short-term plasticity controls memory retention*, we demonstrated the effects of STP on the information retained in continuous working memory. Using our theoretical predictions of drift and diffusion we were able to derive the expected displacement |Δ*φ*| as a function of STP parameters and the frozen noise parameters, which provides a simple link between the resulting Langevin dynamics of bump centers and mutual information (MI) as a measure of working memory retention. Our results can be generalized in several directions. First, the choice of 1*s* of forward integrated time for |Δ*φ*| (Eq (11)) was arbitrary. While a choice of ∼ 2*s* lets the curves in Fig 5E collapse slightly better, we chose 1*s* to avoid further heuristics. Second, we expect values of MI to decrease as the length of the delay period is increased. Our choice of 6.5*s* is comparable to delay periods often considered in behavioral experiments (usually 3-6s) [61, 66, 67]. However, a more rigorous link between the MI measure and the underlying attractor dynamics would be desirable. Indeed, for noisy channels governed by Fokker-Planck equations, this might be feasible [68], but goes beyond the scope of this work.

In Section *Linking theory to experiments: Distractors and network size*, we demonstrated that the high-level description of the microscopic dynamics obtained by our theory allows its parameters to be constrained by experiments. Considering that our model is a simplified description of its biological counterparts (see next paragraph), these demonstrations are to be seen as a proof of principle as opposed to quantitative predictions. However, since distractor inputs can be implemented in silico as well as in behavioral experiments (see e.g. [59]), they could eventually provide a quantitative link between continuous attractor models and working memory systems, by matching the resulting distraction curves. Our theory goes beyond previous models in which these distraction curves had to be extracted through repeated microscopic simulations for single parameter settings [47]. We further used our theory to derive bounds on network parameters, in particular the size of networks, that lead to “tolerable” levels of drift and diffusion in the simplified model. For large magnitudes of frozen noise our theory tends to over-estimate the expected magnitude of drift-fields slightly (cf. Fig 4). Thus, we expect the predictions made here to be upper bounds on network parameters needed to achieve a certain expected displacement. Finally, while the predictions of our theory might deviate from biological networks, they could be applied to accurately characterize the stability of, and the effects of inputs to, bump attractor networks implemented in neuromorphic hardware for robotics applications [69].

Our results show, that strong facilitation (small values of *U*) does not only slow down directed drift [38], but also efficiently suppresses diffusion in spiking continuous attractor models. However, in delayed response tasks involving saccades, that presumably involve continuous attractors in the prefrontal cortex [11, 22], one does observe an increase of variability in time [66]: quickly accumulating systematic errors (alike drift) [61] as well as more slowly increasing variable errors (with variability growing linear in time, alike diffusion) have been reported [60]. Indeed, there are several other possible sources of variability in cortical working memory circuits, which we did not consider here. In particular, we expect that heterogeneous STP parameters [62], noisy synaptic transmission and STP [70] or noisy recurrent weights [38] (see *Random and heterogeneous connectivity* in Materials and methods), for example, will induce further drift and diffusion beyond the effects discussed in this paper. Additionally, variable errors might be introduced elsewhere in the pathway between visual input and motor output (but see [71]) or by input from other noisy local circuits during the delay period [72]. Note that we excluded AMPA currents from the recurrent excitatory interactions [11]. However, since STP acts by presynaptic scaling of neurotransmitter release, it will act symmetrically on both AMPA and NMDA receptors so that an analytical approach similar to the one presented here is expected to work.

Several additional dynamical mechanisms might also influence the stability of continuous attractor working memory circuits. For example, intrinsic neuronal currents that modulate the neuronal excitability [47] or firing-rate adaptation [73] affect bump stability. These and other effects could be accommodated in our theoretical approach by including their linearized dynamics in the calculation of the projection vector (cf. *Projection of dynamics onto the attractor manifold* in Materials and methods). Fast corrective inhibitory feedback has also been shown to stabilize spatial working memory systems in balanced networks [74]. On the timescale of hours to days, homeostatic processes counteract the drift introduced by frozen noise [36]. Finally, inhibitory connections that are distance-dependent [11] and show short-term plasticity [75] could also influence bump dynamics.

We have focused here on ring-attractor models that obtain their stable firing-rate profile due to perfectly symmetric connectivity. Our approach can also be employed to analyze ring-attractor networks with short-term plasticity in which weights show (deterministic or stochastic) deviations from symmetry (see *Frozen noise* in Materials and methods for stochastic deviations). Although not investigated here, continuous line-attractors arising through a different weight-symmetry should be amenable to similar analyses [39]. Finally, it should be noted that adequate structuring of the recurrent connectivity can also positively affect the stability of continuous attractors [14]. For example, translational asymmetries included in the structured heterogeneity can break the continuous attractor into several isolated fixed points, which can lead to decreased diffusion along the attractor manifold [58].

We provided evidence that short-term synaptic plasticity controls the sensitivity of attractor networks to both fast diffusive and frozen noise. Control of short-term plasticity via neuromodulation [76] would thus represent an efficient “crank” for adapting the time scales of computations in such networks. For example, while cortical areas might be specialized to operate in certain temporal domains [7, 77], we show that increasing the strength of facilitation in a task-dependent fashion could yield slower and more stable dynamics, without changing the network connectivity. On the other hand, modulating the time scales of STP could provide higher flexibility in resetting facilitation-stabilized working memory systems to prepare them for new inputs [47], although there might be evidence for residual effects of facilitation between trials [45, 78]. By changing the properties of presynaptic calcium entry [79], inhibitory modulation mediated via GABA_B_ and adenosine A_1_ receptors can lead to increased facilitatory components in rodent cerebellar [80] and avian auditory synapses [81]. Dopamine, serotonin and noradrenaline have all been shown to differentially modulate short-term depression (and facilitation when blocking GABA receptors) at sensorimotor synapses [82]. Interestingly, next to short-term facilitation on the timescale of seconds, other dynamic processes up-regulate recurrent excitatory synaptic connections in prefrontal cortex [62]: synaptic augmentation and post-tetanic potentiation operate on longer time scales (up to tens of seconds), and might be able to support working memory function [83]. While the long time scales of these processes might again render putative short-term memory networks inflexible, there is evidence that they might also be under tight neuromodulatory control [84]. Finally, any changes in recurrent STP properties of continuous attractors (without retuning networks as done here) will also lead to changes in the stable firing rate profiles, with further effects on their dynamical stability (see final section of the Discussion). This interplay of effects remains to be investigated in more detail.

### Comparison to earlier work

Similar to an earlier theoretical approach using a simplified rate model [38], we find that the slowing of drift by facilitation depends mainly on the facilitation parameter *U*, while the time constant *τ*_*u*_ has a less pronounced effect. While the approach of [38] relied on the projection of frozen noise onto the derivative of the first spatial Fourier mode of the bump shape along the ring, here we reproduce and extend this result (1) for arbitrary neuronal input-output relations and (2) a more detailed spatial projection that involves the full synaptic dynamics and the bump shape. While, our theory can also accommodate noisy recurrent connection weights as frozen noise, as used in in [38] (see *Frozen noise* in Materials and methods for derivations), the drifts generated by these heterogeneities were generally small compared to diffusion and the other sources of heterogeneity.

A second study investigated short-term facilitation and showed that it reduces drift and diffusion in a spiking network, for a fixed setting of *U* (although the model of short-term facilitation differs slightly from the one employed here) [47]. Contrary to what we find here, these authors find that an increase in *τ*_*u*_ leads to increased diffusion, while we find that an increase over the range they investigated (∼ 0.5*s* − 4*s*) would decrease the diffusion by a factor of nearly two. More precisely, for our shape of the bump state (which we keep fixed) we predict a reduction from ∼ 26 to ∼ 16 *deg*^2^/*s* for a similar setting of facilitation *U*. These differences might arise from an increasing width of the bump attractor profile for growing facilitation time constants in [47], which would then lead to increased diffusion in our model. Whether this effect persists under the two-equation model of saturating NMDA synapses used there remains to be investigated. Finally, increasing the time constant of recurrent NMDA conductances has been shown to also reduce diffusion [47], in agreement with our theory, according to which the normalization constant *S* increases with *τ*_*s*_ [39].

A study performed in parallel to ours [45] used a similar theoretical approach to calculate diffusion with short-term facilitation in a rate-based model with external additive noise, but did not compare the results for varying facilitation parameters. The authors report a short initial transient of stronger diffusion as synapses facilitate, followed by weaker diffusion that is dictated by the fully facilitated synapses. Our theory, by assuming all synaptic variables to be at steady-state, disregards the initial strong phase of diffusion. We also disregarded such initial transients when comparing to simulations (see *Numerical methods*).

In a study that investigated only a single parameter value for depression (*τ*_*x*_ = 160*ms*, no facilitation) in a network of spiking integrate-and-fire neurons similar to the one investigated here, the authors observed no apparent effect of short-term depression on the stability of the bump [44]. In contrast, we find that stronger short-term depression will indeed increase both diffusion and directed drift along the attractor. Our result agrees qualitatively with earlier studies in rate models, which showed that synaptic depression, similar to neuronal adaptation [10, 85], can induce movement of bump attractors [42, 43, 86, 87]. In particular, simple rate models exhibit a regime where the bump state moves with constant speed along the attractor manifold [42]. We did not find any such directed movement in our networks, which could be due to fast spiking noise which is able to cancel directed bump movement [85].

### Extensions and shortcomings

The coefficients of Eq (4) give clear predictions as to how drift and diffusion will depend on the shape of the bump state and the neural transfer function *F*. The relation is not trivial, since the pre-factors *C*_*i*_ and the normalization constant *S* also depend on the bump shape. For the diffusion strength Eq (5), we explored this relation numerically, by artificially varying the shape of the firing rate profile (while extrapolating other quantities). Although a more thorough analysis remains to be performed, a preliminary analysis shows (see S6 Fig) that diffusion increases both with bump width and top firing rate, consistent with earlier findings [11, 32].

Our theory can be used to predict the shape and effect of drift fields that are generated by localized external inputs due to distractor inputs; see Section *Linking theory to experiments: Distractors and network size*. Any localized external input (excitatory or inhibitory) will cause a deviation Δ*ϕ*_*i*_ from the steady-state firing rates, which, in turn, generates a drift field by Eq (7). This could predict the strength and location of external inputs that are needed to induce continuous shifts of the bump center at given speeds, for example when these attractor networks are designed to track external inputs (see e.g. [10, 88]). It should be noted that in our simple approximation of this distractor scheme, we assume the system to remain at approximately steady-state, i.e. that the bump shape is unaffected by the additional external input, except for a shift of the center position. For example, we expect additional feedback inhibition (through the increased firing of excitatory neurons caused by the distractor input) to decrease bump firing rates. A more in depth study and comparison to simulations will be left for further work.

Our networks of spiking integrate-and-fire neurons are tuned to display balanced inhibition and excitation in the inhibition dominated uniform state [53, 89], while the bump state relies on positive currents, mediated through strong recurrent excitatory connections (cf. [44] for an analysis). Similar to other spiking network models of this class, this mean–driven bump state shows relatively low variability of neuronal inter-spike-intervals of neurons in the bump center [90, 91] (see also next paragraph). Nevertheless, neurons at the flanks of the bump still display variable firing, with statistics close to that expected of spike trains with Poisson statistics (see S7 Fig), which may be because the flank’s position slightly jitters. Since the non-zero contributions to the diffusion strength are constrained to these flanks (cf. Fig 1D), the simple theoretical assumption of Poisson statistics of neuronal firing still matches the spiking network quite well. As discussed in *Short-term plasticity controls diffusion*, we find that our theory over-estimates the diffusion as bump movement slows down for small values of *U*—this may be due to a decrease in firing irregularity in stable bumps in particular in the flank neurons, at which the Poisson assumption becomes inaccurate.

More recent bump attractor approaches allow networks to perform working memory function with a high firing variability also during the delay period [3], in better agreement with experimental evidence [92]. These networks show bi-stability, where both stable states show balanced excitation and inhibition [90] and the higher self-sustained activity in the delay activity is evoked by an increase in fluctuations of the input currents (noise-driven) rather than an increase in the mean input [93]. This was also reported for a ring-attractor network (with distance-dependent connections between all populations), where facilitation and depression are crucial for irregularity of neuronal activity in the self-sustained state [46]. Application of our approach to these setups is left for future work.

## Materials and methods

### Analysis of drift and diffusion with STP

For the following, we define a concatenated 3 ⋅ *N* dimensional column vector of state variables **y** = (**s**^*T*^, **u**^*T*^, **x**^*T*^)^*T*^ of the system Eq (3). Given a (numerical) solution of the stable firing rate profile ${\vec{\phi }}_{0}$ we can calculate the stable fixed point of this system by setting the l.h.s. of Eq (3) to zero. This yields steady-state solutions for the synaptic activations, facilitation and depression variables **y**_0_ = (**s**_0_, **u**_0_, **x**_0_):

$$\begin{array}{cc} s_{0,i} & =\tau_{s}u_{0,i}x_{0,i}\phi_{0,i},\\ u_{0,i} & =U\frac{1+\tau_{u}\phi_{0,i}}{1+U\tau_{u}\phi_{0,i}},\\ x_{0,i} & =\frac{1+U\tau_{u}\phi_{0,i}}{1+U(\tau_{u}\phi_{0,i}+\tau_{u}\tau_{x}\phi_{0,i}^{2}+\tau_{x}\phi_{0,i})}. \end{array}$$ (12)

We then linearize the system Eq (3) at the fixed point **y**_0_, introducing a change of variables consisting of perturbations around the fixed point: **y** = **y**_0_ + *δ* **y** = **y**_0_ + (*δ* **s**^*T*^, *δ* **u**^*T*^, *δ* **x**^*T*^) and *ϕ*_*i*_ = *ϕ*_0,*i*_ + *δϕ*_*i*_. To reach a self-consistent linear system, we further assume a separation of time scales between the neuronal dynamics and the synaptic variables, in that the neuronal firing rate changes as an immediate function of the (slow) input. This allows replacing $\delta \phi_{i}=\frac{d\phi_{i}}{dJ_{i}}|J_{0,i}\underset{j}{\sum }\frac{dJ_{i}}{ds_{j}}\delta s_{j}=\phi_{0,i}^{\prime }\underset{j}{\sum }w_{ij}\delta s_{j}$, where we introduce the shorthand $\phi_{0,i}^{\prime }\equiv \frac{d\phi_{i}}{dJ_{i}}|J_{0,i}$. Finally, keeping only linear orders in all perturbations, we arrive at the linearized system equivalent of Eq (3):

$$\begin{array}{cc} \dot{\delta y} & =(\begin{array}{ccc} -\frac{1}{\tau_{s}}I+D(u_{0}\cdot x_{0}\cdot {\vec{\phi }}_{0}^{\prime })W & D({\vec{\phi }}_{0}\cdot x_{0}) & D({\vec{\phi }}_{0}\cdot u_{0})\\ UD(\left(1-u_{0}\right)\cdot {\vec{\phi }}_{0}^{\prime })W & -\frac{1}{\tau_{u}}I-UD({\vec{\phi }}_{0}) & 0\\ -D(u_{0}\cdot x_{0}\cdot {\vec{\phi }}_{0}^{\prime })W & -D(x_{0}\cdot {\vec{\phi }}_{0}) & -\frac{1}{\tau_{x}}I-D({\vec{\phi }}_{0}\cdot u_{0}) \end{array})\delta y\\ & \equiv K\delta y \end{array}$$ (13)

Here, dots between vectors indicate element-wise multiplication, the operator $D:R^{n}\to R^{n\times n}$ creates diagonal matrices from vectors, and *W* = (*w*_*ij*_) is the synaptic weight matrix of the network.

#### Projection of dynamics onto the attractor manifold

To project the dynamical system Eq (13) onto movement of the center position *φ* of the firing rate profile, we assume that *N* is large enough to treat the center position *φ* as a continuous variable. We also assume that the network implements a ring-attractor: the system dynamics are such that the firing rate profile ${\vec{\phi }}_{0}$ can be freely shifted to different positions along the ring, changing the center position *φ*, while retaining the same shape. All other possible directions of change in this system are assumed to be constrained by the system dynamics. In the system at hand, this implies that the matrix *K* of Eq (13), which captures the linearized dynamics around any of these fixed points, will have a *zero eigenvalue* corresponding to the eigenvector of a change of the dynamical variables under a change of position *φ*, while all other eigenvalues are negative [39].

Formally, the column eigenvector to the eigenvalue 0 is given by changes in the state variables as the bump center position *φ* is translated along the manifold:

$$\begin{array}{c} e_{r}=\frac{dy_{0}}{d\varphi }=({\frac{ds_{0}}{d\varphi }}^{T},{\frac{du_{0}}{d\varphi }}^{T},{\frac{dx_{0}}{d\varphi }}^{T}{)}^{T}. \end{array}$$ (14)

Let *e*_*l*_ be the associated row left-eigenvector (also to eigenvalue 0) of *K*, normalized such that:

$$\begin{array}{c} e_{l}\cdot e_{r}=1. \end{array}$$ (15)

In Section 1 of S1 Text, we show that the eigenvector *e*_*l*_ projects the system Eq (13) onto dynamics of of the center position:

$$\begin{array}{c} \dot{\varphi }=e_{l}\dot{\delta y}=e_{l}K\delta y=e_{l}\cdot 0\cdot \delta y. \end{array}$$ (16)

Under the linearized ring-attractor dynamics *K*, the center position is thus not subject to any dynamics, making it susceptible to any displacements by noise.

#### Calculation of the left eigenvector *e*_*l*_

If the matrix *K* is symmetric, the left and right eigenvectors *e*_*l*_ and *e*_*r*_ for the same eigenvalue 0 are the transpose of each other. Unfortunately, here this is not the case (see Eq (13)), and we need to compute the unknown vector *e*_*l*_, which will depend on the coefficients of the known vector *e*_*r*_. In particular, we look for a parametrized vector **y**′(**y**) = (**t**^*T*^(**y**), **v**^*T*^(**y**), **z**^*T*^(**y**))^*T*^ that for **y** = *e*_*r*_ fulfills the transposed eigenvalue equation of the left eigenvector:

$$\begin{array}{c} K^{T}y^{\prime }\left(e_{r}\right)=0. \end{array}$$ (17)

In Section 2 of S1 Text, we derive variables **y**′ that fulfill the transposed dynamics ${\dot{y}}^{\prime }=K^{T}y^{\prime }$ and for which it holds that ${\dot{y}}^{\prime }\left(e_{r}\right)=0$, thus fulfilling the condition Eq (17). In this case we know that (due to uniqueness of the 1-dimensional eigenspace associated to the 0 eigenvalue) the vector **y**′^*T*^ is proportional to *e*_*l*_:

$$\begin{array}{c} e_{l}=\frac{1}{S}y^{\prime }{\left(e_{r}\right)}^{T}=({\frac{dJ_{0}}{d\varphi }}^{T},(\alpha_{1}\frac{du_{0}}{d\varphi }+\alpha_{2}\frac{dx_{0}}{d\varphi }{)}^{T},(\beta_{1}\frac{du_{0}}{d\varphi }+\beta_{2}\frac{dx_{0}}{d\varphi }{)}^{T}), \end{array}$$ (18)

where *S* is a proportionality constant and $\frac{dJ_{0,i}}{d\varphi }=\sum_{j}w_{ij}\frac{ds_{0,j}}{d\varphi }$ is the change of the steady-state input arriving at neuron *i* under shifts of the center position *φ*.

Finally, the proportionality constant *S* can be calculated by using Eq (18) in Eq (15) (see Section 3 of S1 Text for details):

S=y′(er)T·er=U∑i(dJ0,idφ)2ϕi′[Uϕ0,i(τu(τxϕ0,i+1)+τx)+1]3[12τs[τuϕ0,i(Uτuϕ0,i+2)+1][Uϕ0,i(τu(τxϕ0,i+1)+τx)+1]−ϕ0,i[(U−1)τu2+Uτx2(τuϕ0,i+1)(τuϕ0,i(Uτuϕ0,i+2)+1)]−(U−1)Uτu2τxϕ0,i(τuϕ0,i+1)(Uτuϕ0,i+1)], (19)

where $\phi_{0,i}^{\prime }=\frac{d\phi_{i}}{dJ_{i}}|J_{0,i}$ is the linear change of the firing rate of neuron *i* at its steady-state input *J*_0,*i*_.

#### Diffusion

To be able to describe diffusion on the continuous attractor, we need to extend the model by a treatment of the noise induced into the system through the variable process of neuronal spike emission. Starting from Eq (3), we assume that neurons *i* fire according to independent Poisson processes *ξ*_*i*_(*t*) = ∑_*k*_ *δ*(*t* − *t*_*i*,*k*_), where *t*_*i*,*k*_ is a Poisson point process with time-dependent rate *ϕ*_*i*_. The variability of the point process *ξ*_*i*_(*t*) introduces noise in the synaptic variables. We assume that the shot-noise (jump-like) nature of this process is negligible, given that we average all individual contributions over the network (see below), allowing us to capture the neurally induced variability simply as white noise with variance proportional to the incoming firing rates [48, 53], $\xi_{i}\left(t\right)=\phi_{i}+\sqrt{\phi_{i}}\cdot \eta_{i}\left(t\right)$, where *η*_*i*_ are white Gaussian noise processes with mean 〈*η*_*i*_〉 = 0, and correlation function 〈*η*_*i*_(*t*)*η*_*j*_(*t*′)〉 = *δ*(*t* − *t*′)*δ*_*ij*_. This model of *ξ*_*i*_(*t*) preserves the mean and the auto-correlation function of the original Poisson processes. Here, we introduce diffusive noise for each synaptic variable separately, but later average their *linear* contributions over the large population, when projecting onto movement along the continuous manifold (see below, and also [39], Supplementary Material] for a discussion).

Substituting the noisy processes *ξ*_*i*_(*t*) for *ϕ*_*i*_(*t*) in Eq (3) results in the following system of 3⋅*N* coupled Ito-SDEs:

$$\begin{array}{ccc} {\dot{s}}_{i} & = & -\frac{s_{i}}{\tau_{s}}+u_{i}x_{i}(\phi_{i}+\eta_{i}\sqrt{\phi_{i}}),\\ {\dot{u}}_{i} & = & -\frac{u_{i}-U}{\tau_{u}}+U\left(1-u_{i}\right)(\phi_{i}+\eta_{i}\sqrt{\phi_{i}}),\\ {\dot{x}}_{i} & = & -\frac{x_{i}-1}{\tau_{x}}-u_{i}x_{i}(\phi_{i}+\eta_{i}\sqrt{\phi_{i}}). \end{array}$$ (20)

Note that the noise inputs *η*_*i*_ to the synaptic variables for neuron *i* are all identical, since they result from the same presynaptic spike train.

Linearizing this system around the noise-free steady-state Eq (12) and considering only the unperturbed noise (we neglect multiplicative noise terms by replacing the terms $\sqrt{\phi_{i}}\to \sqrt{\phi_{0,i}}$), we arrive at the linearized system equivalent of Eq (20):

$$\begin{array}{c} \dot{\delta y}=K\delta y+(\begin{array}{c} \vec{\eta }u_{0}x_{0}\sqrt{{\vec{\phi }}_{0}}\\ \vec{\eta }U\left(1-u_{0}\right)\sqrt{{\vec{\phi }}_{0}}\\ -\vec{\eta }u_{0}x_{0}\sqrt{{\vec{\phi }}_{0}} \end{array})\equiv K\delta y+L. \end{array}$$ (21)

Note that the same vector of white noises $\vec{\eta }\equiv (\eta_{1},\ldots ,\eta_{n}{)}^{T}$ appears three times.

Left-multiplying this system with the eigenvector *e*_*l*_ yields a stochastic differential equation for the center position (cf. Eq (16)):

$$\begin{array}{c} \dot{\varphi }=e_{l}\dot{\delta y}=0\cdot K+e_{l}L=\sum_{k}e_{l,k}L_{k} \end{array}$$ (22)

Through the normalization by *S* (Eq (18)), which sums over all neurons, the individual contributions *e*_*l*,*k*_ become small as the number of neurons *N* increases (this scaling is made explicit in *System size scaling*). Thus, for large networks we average the small contributions of many single noise sources, which validates the diffusion approximation above.

In Section 4 of S1 Text, we show that we can rewrite Eq (22) by introducing a single Gaussian white noise process with intensity *B* (Eq (5) of the main text), that matches the correlation function of the summed noises:

$$\begin{array}{c} \dot{\varphi }=\sqrt{B}\eta , \end{array}$$ (23)

where *η* is a white Gaussian noise process with 〈*η*〉 = 0 and 〈*η*(*t*)*η*(*t*′)〉 = *δ*(*t* − *t*′). Note, that the value of *B* is the same under changes of the center position *φ*: these correspond to index-shifting (mod *N*) all vectors in Eq (5), which leaves the sum invariant.

#### Drift

While the diffusion coefficient calculated above is invariant with respect to shifts of the bump center, the directed drift introduced by frozen variability depends on the momentary bump center position *φ*. In the following we compare the heterogeneous network with bump centered at *φ* to a homogeneous network (without frozen noise) with the bump also centered at *φ*. The unperturbed firing rate profile in the homogeneous network with bump at *φ* will be denoted by ${\vec{\phi }}_{0}\left(\varphi \right)$, which is the standard profile ${\vec{\phi }}_{0}$ but centered at *φ*. Since we choose the standard profile to be centered at −*π*, we have ${\vec{\phi }}_{0}={\vec{\phi }}_{0}\left(-\pi \right)$.

We want to derive a compact expression for the directed drift of the bump in the heterogeneous network with frozen noise. Given a bump center position *φ*, we first shift the origin of the coordinate system that describes the angular position on the ring of neurons such that the firing rate profile is centered at the standard position *φ*_0_ = −*π*. In a system with frozen variability, the actual firing rate profile of the bump is

$$\begin{array}{c} {\vec{\phi }}_{0}+\Delta \vec{\phi }\left(\varphi \right). \end{array}$$ (24)

where $\Delta \vec{\phi }\left(\varphi \right)$ summarize the linear firing rate perturbations caused by a small amount of heterogeneities. These firing rate perturbations stem from any deviation of the neural system from the “baseline” case and change with the center position *φ* of the bump. The resulting drift field derived from a linearization of the dynamics will thus depend on the center position. In subsection *Frozen noise* we calculate the perturbations induced by random network connectivity, as well as heterogeneous leak reversal-potentials in excitatory neurons of the spiking network.

The firing rate perturbations Eq (24) add an additional term in the linearized equations Eq (21):

$$\begin{array}{c} \delta \dot{y}=K\delta y+(\begin{array}{c} x_{0}u_{0}\Delta \vec{\phi }\left(\varphi \right)\\ U\left(1-u_{0}\right)\Delta \vec{\phi }\left(\varphi \right)\\ -x_{0}u_{0}\Delta \vec{\phi }\left(\varphi \right) \end{array})+L. \end{array}$$ (25)

As before, we left-multiply by the left eigenvector *e*_*l*_, thereby projecting the dynamics onto changes of the center position. This eliminates the linear response kernel *K* and yields a drift-term in the SDE Eq (23) (see Section 5 of S1 Text for details):

$$\begin{array}{c} \dot{\varphi }=\sum_{i}\frac{dJ_{0,i}}{d\varphi }\frac{1}{S}\frac{U(1+2\tau_{u}\phi_{0,i}+U\tau_{u}^{2}\phi_{0,i}^{2})}{(U\phi_{0,i}(\tau_{u}\tau_{x}\phi_{0,i}+\tau_{u}+\tau_{x})+1){}^{2}}\Delta \phi_{i}\left(\varphi \right)+\sqrt{B}\eta . \end{array}$$ (26)

Here, *ϕ*_0,*i*_ is the firing rate of the *i*th neuron in a homogeneous network with the bump centered at −*π* and Δ*ϕ*_*i*_(*φ*) is the firing rate change of this neuron caused by heterogeneities where the heterogeneities are calculated under the assumption that (before shift of the coordinate system) the bump is at *φ*.

In the above equation, we have assumed that the number of neurons *N* is large enough to treat the center position as a continuous variable *φ* ∈ [−*π*, *π*) with the associated drift-field *A*(*φ*) in Eq (7). In practice, we calculate this drift field according to the first term in Eq (26) for each realizable center position $\varphi_{k}=k\frac{2\pi }{N}-\pi$ (for 0 ≤ *k* < *N*), which yields a discretized field. It is important to note that this field will vary nearly continuously with changes in these discretized center positions. Intuitively, the sum weighs the vector $\Delta \vec{\phi }\left(\varphi_{k}\right)$ of firing-rate perturbations with a smooth function of the smoothly varying firing-rate profile ${\vec{\phi }}_{0}$ (the coefficients in the sum). Shifts in the center position *φ*_*k*_ yield (to first order) index-shifts in the vector of firing-rate perturbations (see *Frozen noise*), equivalent to index-shifts of the vector of firing rates ${\vec{\phi }}_{0}$. Thus, small changes in center positions will lead to small changes in the summands of Eq (26). While our results validate the approach, a more rigorous proof of these arguments will be left for future work.

### Spiking network model

Spiking simulations are based on a variation of a popular ring-attractor model of visuospatial working memory of [11] (and used with variations in [27, 29, 32, 36, 47]). The recurrent excitatory connections of the original network model have been simplified, to allow for faster simulation as well as analytical derivations of the recurrent synaptic activation. The implementation details are given below, however the major changes are: 1) all recurrent excitatory conductances are voltage independent; 2) a model of synaptic short-term plasticity via facilitation and depression [49, 94, 95] is used to dynamically regulate the weights of the incoming spike-trains 3) recurrent excitatory conductances are computed as linear filters of the weighted incoming spike trains instead of the second-order kinetics for NMDA saturation used in [11].

#### Neuron model

Neurons are modeled by leaky integrate-and-fire dynamics with conductance based synaptic transmission [11, 50]. The network consists of recurrently connected populations of *N*_*E*_ excitatory and *N*_*I*_ inhibitory neurons, both additionally receiving external spiking input with spike times generated by *N*_ext_ independent, homogeneous Poisson processes, with rates *ν*_*ext*_. We assume that external excitatory inputs are mediated by fast AMPA receptors, while, for simplicity, recurrent excitatory currents are mediated only by slower NMDA channels (as in [11]).

The dynamics of neurons in both excitatory and inhibitory populations are governed by the following system of differential equations indexed by *i* ∈ {0, …, *N*_*E*/*I*_ − 1}:

$$\begin{array}{ccc} C_{m}{\dot{V}}_{i}\left(t\right) & = & -I_{i}^{\text{L}}\left(t\right)-I_{i}^{\text{Ext}}\left(t\right)-I_{i}^{\text{I}}\left(t\right)-I_{i}^{\text{E}}\left(t\right),\\ I_{i}^{P} & = & g_{P}\;s_{i}^{P}\left(t\right)\;(V_{i}\left(t\right)-V_{P}), \end{array}$$ (27)

where *P* ∈ {L,Ext,I,E}, *V* denotes voltages (membrane potential) and *I* denotes currents. Here, *C*_m_ is the membrane capacitance and *V*_L_, *V*_E_, *V*_I_ are the reversal potentials for leak, excitatory currents, and inhibitory currents, respectively. The parameters *g*_*P*_ for *P* ∈ {L,Ext,I,E} are fixed scales for leak (L), external input (Ext) and recurrent excitatory (E) and inhibitory (I) synaptic conductances, which are dynamically gated by the unit-less gating variables $s_{i}^{P}\left(t\right)$. These gating variables are described in detail below, however we set the leak conductance gating variable to $s_{i}^{\text{L}}=1$. For excitatory neurons, we refer to the excitatory and inhibitory conductance scales by *g*_EE_ ≡ *g*_E_ and *g*_EI_ ≡ *g*_I_, respectively. Similarly, for inhibitory neurons, we refer to the excitatory and inhibitory conductance scales by *g*_IE_ ≡ *g*_E_ and *g*_II_ ≡ *g*_I_, respectively.

The model neuron dynamics (Eq 27) are integrated until their voltage reaches a threshold *V*_thr_. At any such time, the respective neuron emits a spike and its membrane potential is reset to the value *V*_res_. After each spike, voltages are clamped to *V*_res_ for a refractory period of *τ*_ref_. See the Tables in S1 and S2 Tables. for parameter values used in simulations.

#### Synaptic gating variables and short-term plasticity

The unit-less synaptic gating variables $s_{i}^{P}\left(t\right)$ for *P* ∈ {Ext,I} (external and inhibitory currents) are exponential traces of the spike trains of all presynaptic neurons *j* with firing times *t*_*j*_:

$$\begin{array}{c} {\dot{s}}_{i}^{P}\left(t\right)=-\frac{s_{i}^{P}\left(t\right)}{\tau_{P}}+\sum_{j\in \text{pre}(P)}w_{ij}^{P}\sum_{t_{j}}\delta (t-t_{j}), \end{array}$$ (28)

where pre(P) indicates all neurons presynaptic to the neuron *i* for the the connection type *P*. The factors $w_{ij}^{P}$ are unit-less synaptic efficacies for the connection from neuron *j* to neuron *i*. For the excitatory gating variables of inhibitory neurons $s_{i}^{\text{IE}}$ (IE denotes connections from E to I neurons) we also use the linear model of Eq (28) with time constant *τ*_*IE*_ = *τ*_*E*_.

For excitatory to excitatory conductances, we use a well established model of synaptic short-term plasticity (STP) [49, 94, 95] which provides dynamic scaling of synaptic efficacies depending on presynaptic firing. This yields two additional dynamical variables, the facilitating synaptic efficacy *u*_*j*_(*t*), as well as the fraction of available synaptic resources *x*_*j*_(*t*) of the outgoing connections of a presynaptic neuron *j*, which are implemented according to the following differential equation:

$$\begin{array}{ccc} {\dot{u}}_{j} & = & -\frac{1}{\tau_{u}}(u_{j}-U)+U(1-u_{j}^{-})\sum_{t_{j}}\delta \left(t-t_{j}\right),\\ {\dot{x}}_{j} & = & -\frac{1}{\tau_{x}}(x_{j}-1)-x_{j}^{-}u_{j}^{-}\sum_{t_{j}}\delta \left(t-t_{j}\right). \end{array}$$ (29)

Here, the indices $u_{j}^{-}$ and $x_{j}^{-}$ indicate that for the incremental update of the variables upon spike arrival, we use the values of the respective variables immediately before the spike arrival [95]. Note that the variable *U* appears in the equation for *u*(*t*) both as the steady-state value in the absence of spikes and as a scale for the update per spike.

The dynamics of recurrent excitatory-to-excitatory transmission with STP are then given by gating variables that linearly filter the incoming spikes scaled by facilitation and depression:

$$\begin{array}{ccc} s_{i}^{\text{EE}}\left(t\right) & = & \sum_{j\in \text{pre}(\text{EE})}w_{ij}^{\text{EE}}s_{j} \end{array}$$ (30)

$$\begin{array}{ccc} {\dot{s}}_{j} & = & -\frac{s_{j}}{\tau_{s}}+\sum_{t_{j}}\delta (t-t_{j})u_{j}^{-}\left(t\right)x_{j}^{-}\left(t\right). \end{array}$$ (31)

Here, pre(EE) indicates all excitatory neurons that make synaptic connections to the neuron *i*. See ‘S2 Table’ for synaptic parameters used in simulations. Note that a synapse *j* that has been inactive for a long time is described by variables $x_{j}^{-}=1$ and $u_{j}^{-}=U$ and *s*_*j*_ = 0 so that the initial strength of the synaptic connection is $Uw_{ij}^{EE}$ [49].

The system of Eqs (29)–(31) is a spiking variant of the rate-based dynamics of Eq (3), with $s_{i}^{\text{EE}}$ a variable related to the input *J*_*i*_ (cf. Eq (2)). In Subsection *Firing rate approximation* we will make this link explicit.

#### Network connectivity

All connections except for the recurrent excitatory connections are all-to-all and uniform, with unit-less connection strengths set to $w_{ij}^{\text{I}}=w_{ij}^{\text{ext}}=1$ and for inhibitory neurons additionally $w_{ij}^{\text{E}}=1$. The recurrent excitatory connections are distance-dependent and symmetric. Each neuron of the excitatory population with index *i* ∈ {0, …, *N*_*E*_ − 1} is assigned an angular position $\theta_{i}=i\cdot \frac{2\pi }{N_{E}}\in \left[0,2\pi \right)$. Recurrent excitatory connection weights $w_{ij}^{\text{EE}}$ from neuron *j* to neuron *i* are then given by the Gaussian function *w*^EE^(*θ*) as (see the Table in S2 Table for parameters used in simulations):

$$\begin{array}{cc} w_{ij}^{\text{EE}} & =w^{\text{EE}}\left(\theta_{i}-\theta_{j}\right)\\ & =w_{0}+(w_{+}-w_{0})\text{exp}(-[\text{min}(|\theta_{i}-\theta_{j}|,2\pi -|\theta_{i}-\theta_{j}|){]}^{2}\frac{1}{2\sigma_{w}^{2}}). \end{array}$$ (32)

Additionally, for each neuron we keep the integral over all recurrent connection weights normalized, resulting in the normalization condition $\frac{1}{2\pi }\int_{-\pi }^{\pi }d\varphi w^{\text{EE}}\left(\varphi \right)=1.$ This normalization ensures that varying the maximum weight *w*_+_ will not change the total recurrent excitatory input if all excitatory neurons fire at the same rate. Here, we choose *w*_+_ as a free parameter constraining the baseline connection weight to:

$$\begin{array}{c} w_{0}=\frac{w_{+}\sigma_{w}\text{erf}(\frac{\pi }{\sqrt{2}\sigma_{w}})-\sqrt{2\pi }}{\sigma_{w}\text{erf}(\frac{\pi }{\sqrt{2}\sigma_{w}})-\sqrt{2\pi }}. \end{array}$$

#### Firing rate approximation

We first replace the synaptic activation variables *s*^*P*^(*V*, *t*) for *P* ∈ {I, ext} by their expectation values under input with Poisson statistics. We assume that the inhibitory population fires at rates *ν*_*I*_. For the linear synapses this yields

$$\begin{array}{c} \langle s^{\text{ext}}\rangle =\tau_{\text{ext}}N_{\text{ext}}\nu_{\text{ext}}, \end{array}$$ (33)

$$\begin{array}{c} \langle s^{\text{I}}\rangle =\tau_{\text{I}}N_{I}\nu_{\text{I}}. \end{array}$$ (34)

For the recurrent excitatory-to-excitatory synapses with short-term plasticity, we set the differential Eq (29) to zero, and also average them over the Poisson statistics. Akin to the “mean-field” model of [49], we average the steady-state values of facilitation and depression separately over the Poisson statistics. This implicitly assumes that facilitation and depression are statistically independent, with respect to the distributions of spike times—while this is not strictly true, the approximations work well, as has been previously reported [49]. This allows a fairly straightforward evaluation of the mean steady-state value of the combined facilitation and depression variables 〈*u*_*j*_ *x*_*j*_〉, under the assumption that the neuron *j* fires at a mean rate *ν*_*j*_ with Poisson statistics, and yields rate approximations of the steady-state values similar to Eq (12):

$$\begin{array}{c} \left\langle u_{j}x_{j}\right\rangle =\left\langle u_{j}\right\rangle \left\langle x_{j}\right\rangle =\frac{U(\nu_{j}\tau_{u}+1)}{U\nu_{j}\left(\tau_{u}+\tau_{x}+\nu_{j}\tau_{u}\tau_{x}\right)+1}. \end{array}$$ (35)

We now assume that the excitatory population of *N*_*E*_ neurons fires at the steady-state rates *ϕ*_*j*_ (0 ≤ *j* < *N*). To calculate the synaptic activation of excitatory-to-excitatory connections $\langle s_{i}^{\text{EE}}\rangle$, we set Eq (30) to zero, and average over Poisson statistics (again neglecting correlations), which yields〈*s*_*j*_〉 = *τ*_*E*_〈*u*_*j*_ *x*_*j*_〉*ϕ*_*j*_ and $\langle s_{i}^{\text{EE}}\rangle =\sum_{j}w_{ij}^{\text{EE}}\tau_{E}\langle u_{j}x_{j}\rangle \phi_{j}$. Let the the normalized steady-state input *J*_*i*_ be:

$$\begin{array}{c} J_{i}\equiv \frac{1}{N_{\text{E}}}\left\langle s_{i}^{\text{EE}}\right\rangle =\frac{1}{N_{\text{E}}}\sum_{j}w_{ij}^{\text{EE}}\left\langle s_{j}\right\rangle . \end{array}$$ (36)

The steady-state input Eq (36) links the general framework of Eq (2) to the spiking network. The additional factor 1/*N*_*E*_ is introduced to make the scaling of the excitatory-to-excitatory conductance with the size of the excitatory population *N*_E_ explicit, which will be used in *System size scaling*. To see this, we assume that the excitatory conductance scale of excitatory neurons *g*_EE_ is scaled such that the total conductance is invariant under changes of *N*_E_ [96]: $g_{\text{EE}}={\tilde{g}}_{\text{EE}}/N_{E}$, for some fixed value ${\tilde{g}}_{\text{EE}}$. This yields the total excitatory-to-excitatory conductance $g_{\text{EE}}s_{i}^{\text{EE}}={\tilde{g}}_{\text{EE}}J_{i}$ with *J*_*i*_ as introduced above, where the scaling with *N*_E_ is now shifted to the input variable *J*_*i*_.

For the synaptic activation of excitatory to inhibitory connections, we get the mean activations:

$$\begin{array}{c} \langle s^{\text{IE}}\rangle =\tau_{\text{E}}\sum_{j}\phi_{j}. \end{array}$$ (37)

We then follow [50] to reduce the differential equations of Eq (27) to a dimensionless form. The main difference consists in the absence of the voltage dependent NMDA conductance, which is achieved by setting the two associated parameters *β* → 0, *γ* → 0 in [50], to arrive at:

$$\begin{array}{ccc} \tau_{i}{\dot{V}}_{i} & = & -(V_{i}-V_{L})+\mu_{i}+\sigma_{i}\sqrt{\tau_{i}}\eta_{i}\left(t\right) \end{array}$$

$$\begin{array}{ccc} S_{i} & = & 1+T_{I}\nu_{I}+T_{\text{ext}}\nu_{\text{ext}}+T_{E}J_{i} \end{array}$$ (38)

$$\begin{array}{ccc} \mu_{i}S_{i} & = & (V_{I}-V_{L})T_{I}\nu_{I}+(V_{E}-V_{L})T_{\text{ext}}\nu_{\text{ext}}+(V_{E}-V_{L})T_{E}J_{i} \end{array}$$ (39)

$$\begin{array}{ccc} \sigma_{i} & = & \frac{g_{\text{ext}}}{C_{m}}(\langle V\rangle -V_{E})\tau_{\text{ext}}\sqrt{\tau_{i}N_{\text{ext}}\nu_{\text{ext}}}. \end{array}$$ (40)

$$\begin{array}{ccc} \tau_{i} & = & \frac{C_{m}}{g_{L}S_{i}} \end{array}$$

$$\begin{array}{ccc} \left\langle \eta_{i}\left(t\right)\right\rangle & = & 0 \end{array}$$

$$\begin{array}{ccc} \left\langle \eta_{i}\left(t\right)\eta_{i}\left(t^{\prime }\right)\right\rangle & = & \frac{1}{\tau_{\text{ext}}}\text{exp}\left(-\frac{\left|t-t^{\prime }\right|}{\tau_{\text{ext}}}\right) \end{array}$$ (41)

where $T_{\text{ext}}=N_{\text{ext}}\tau_{\text{ext}}\frac{g_{\text{ext}}}{g_{L}},\;T_{I}=N_{I}\tau_{I}\frac{g_{I}}{g_{L}}$ are effective timescales of external and inhibitory inputs, and $T_{E}=N_{E}\frac{g_{E}}{g_{L}}$ is a dimensionless scale for the excitatory conductance. Here, *μ*_*i*_ is the bias of the membrane potential due to synaptic inputs, and *σ*_*i*_ measures the scale of fluctuations in the membrane potential due to random spike arrival approximated by the Gaussian process *η*_*i*_.

The mean firing rates *F* and mean voltages 〈*V*_*i*_〉 of populations of neurons governed by this type of differential equation can then be approximated by:

$$\begin{array}{ccc} F[\mu_{i},\sigma_{i},\tau_{i}] & = & (\tau_{\text{ref}}+\sqrt{\pi }\tau_{i}\int_{\beta (\mu_{i},\sigma_{i})}^{\alpha (\mu_{i},\sigma_{i})}du\;\text{exp}\left(u^{2}\right)[1+\text{erf}(u)]{)}^{-1} \end{array}$$ (42)

$$\begin{array}{ccc} \alpha (\mu_{i},\sigma_{i},\tau_{i}) & = & \frac{V_{\text{reset}}-V_{L}-\mu_{i}}{\sigma_{i}}(1+\frac{\tau_{\text{ext}}}{2\tau_{i}})+1.03\sqrt{\frac{\tau_{\text{ext}}}{\tau_{i}}}-\frac{\tau_{\text{ext}}}{\tau_{i}}, \end{array}$$ (43)

$$\begin{array}{ccc} \beta (\mu_{i},\sigma_{i}) & = & \frac{V_{\text{reset}}-V_{L}-\mu_{i}}{\sigma_{i}}, \end{array}$$ (44)

$$\begin{array}{ccc} \left\langle V_{i}\right\rangle & = & \mu_{i}+V_{L}-(V_{\text{thr}}-V_{\text{reset}})\phi_{i}\tau_{i}. \end{array}$$ (45)

#### Derivatives of the rate prediction

Here we calculate derivatives of the input-output relation (Eq (42)) that will be used below in *Frozen noise*.

The expressions for drift and diffusion (see *Analysis of drift and diffusion with STP*) contain the derivative $\phi_{i}^{\prime }=\frac{dF}{dJ}|J_{i}$ of the input-output relation *F* (Eq (42)) with respect to the recurrent excitatory input *J*_*i*_. Note, that *F* depends on *J*_*i*_ through all three arguments *μ*_*i*_, *σ*_*i*_ and *τ*_*i*_. First, we define *X*(*u*) ≡ exp(*u*^2^)[1 + erf(*u*)], and the shorthand *F*_*i*_ = *F*[*μ*_*i*_, *σ*_*i*_, *τ*_*i*_]. The derivative can then be readily evaluated as (to shorten the notation in the following, we skip noting the evaluation points for derivatives in the following):

$$\begin{array}{c} \frac{dF}{dJ_{i}}=-F_{i}\frac{T_{E}}{S_{i}}(F_{i}\tau_{\text{ref}}-1)+\sqrt{\pi }F_{i}^{2}\tau_{i}[X\left(\beta \right)\frac{d\beta }{dJ_{i}}-X\left(\alpha \right)\frac{d\alpha }{dJ_{i}}], \end{array}$$ (46)

$$\begin{array}{cc} \frac{d\alpha /\beta }{dJ_{i}}= & \left(\frac{\partial \alpha /\beta }{\partial \mu_{i}}+\frac{\partial \alpha /\beta }{\partial \sigma_{i}}\frac{\partial \sigma_{i}}{\partial \langle V_{i}\rangle })\frac{T_{E}}{S_{i}}(-\mu_{i}+(V_{E}-V_{L})\right)\\ & -(\frac{\partial \alpha /\beta }{\partial \sigma_{i}}[\frac{\partial \sigma_{i}}{\partial \tau_{i}}-\frac{\partial \sigma_{i}}{\partial \langle V_{i}\rangle }(V_{\text{thr}}-V_{\text{reset}})\phi_{i}]+\frac{\partial \alpha /\beta }{\partial \tau_{i}})\frac{T_{E}}{S_{i}}\tau_{i}. \end{array}$$

where *α*/*β* stands as a placeholder for either function, and the expressions for *α* and *β* are given in Eqs (43) and (44).

A second expression involving the derivative of Eq (42) is $\frac{d\phi_{0,i}}{d\Delta_{i}^{\text{L}}}$ which appears in the theory when estimating firing rate perturbations caused by frozen heterogeneities in the leak potentials of excitatory neurons (see Eq (54)). The resulting derivatives are almost similar, which can be seen by the fact that replacing $V_{\text{L}}\to V_{\text{L}}+\Delta_{i}^{\text{L}}$ in Eq (27) only leads to an additional term $\Delta_{i}^{\text{L}}$ in Eq (39). Thus, for neuron *i* the derivative can be evaluated to

$$\begin{array}{c} \frac{dF}{d\Delta_{i}^{L}}=\sqrt{\pi }F_{i}^{2}\tau_{i}[X\left(\beta \right)\frac{\partial \beta }{\partial \Delta_{i}^{\text{L}}}-X\left(\alpha \right)\frac{\partial \alpha }{\partial \Delta_{i}^{\text{L}}}], \end{array}$$ (47)

$$\begin{array}{c} \frac{d\alpha /\beta }{d\Delta_{i}^{\text{L}}}=(\frac{\partial \alpha /\beta }{\partial \mu_{i}}+\frac{\partial \alpha /\beta }{\partial \sigma_{i}}\frac{\partial \sigma_{i}}{\partial \langle V_{i}\rangle })\frac{1}{S_{i}}. \end{array}$$

In practice, given a vector *ϕ*_*i*,0_ of firing rates in the attractor state, as well as the mean firing rate of inhibitory neurons *ν*_*I*_, we evaluate the right hand side of Eqs (46) and (47) by replacing *F*_*i*_ → *ϕ*_*i*,0_. This allows efficiently calculating the derivatives without having to perform any numerical integration. The two terms will be exactly equal if *ϕ*_0,*i*_ is a self-consistent solution of Eq (42) for firing rates of the excitatory neurons across the network. We used numerical estimates of *ϕ*_*i*,0_ and *ν*_*I*_ that were measured from simulations and were very close to firing-rate predictions for all networks we investigated.

#### Optimization of network parameters

We used an optimization procedure [97] to retune network parameters to produce approximately similar bump shapes as the parameters of short-term plasticity are varied. Briefly, we replace the network activity *ϕ*_*j*_ in the total input *J*_*i*_ of Eq (36) by a parametrization

$$\begin{array}{c} g\left(\theta_{j}\right)=g_{0}+g_{1}\text{exp}(-[\frac{\left|\theta_{j}\right|}{g_{\sigma }}{]}^{g_{r}}). \end{array}$$ (48)

Approximating sums $\frac{1}{N_{E}}\sum_{j=0}^{N_{E}-1}$ with integrals $\frac{1}{2\pi }\int_{-\pi }^{\pi }d\varphi$ we arrive at

$$\begin{array}{c} J_{i}\left(g\right)\approx \frac{1}{2\pi }\int_{-\pi }^{\pi }d\varphi \;w^{\text{EE}}(\theta_{i}-\varphi )\left\langle s_{j}\right\rangle \left(g(\varphi )\right), \end{array}$$

where *J*_*i*_(*g*) indicates that the total input depends on the parameters *g*_0_, *g*_1_, *g*_*σ*_, *g*_*r*_ of the parametrization *g*.

We then substitute this relation in Eq (42) to arrive at a self-consistency relation between the parametrized network activity *g*(*θ*_*i*_) at the position of neuron *i* and the firing-rate *F* predicted by the theory:

$$\begin{array}{c} g\left(\theta_{i}\right)=F[\mu_{i}\left(g\right),\sigma_{i}\left(g\right),\tau_{i}\left(g\right),\left\langle V_{i}\right\rangle \left(g\right)]. \end{array}$$ (49)

The argument *g* indicates the dependence of quantities upon the parameters of the bump parametrization Eq (48). The explicit dependence of the voltage 〈*V*_*i*_〉 on *g* is obtained by substituting *ϕ*_*i*_ → *g*(*θ*_*i*_) in Eq (45).

We then optimized networks to fulfill Eq (49). First, we imposed the following targets for the parameters of *g*: *g*_0_ = 0.1Hz, *g*_1_ = 40Hz, *ν*_*E*,basal_ = 0.5Hz, *ν*_*I*,basal_ = 3Hz. For all networks we chose *w*_+_ = 4.0, *g*_*r*_ = 2.5. The following parameters were then optimized: *ν*_*I*_, *g*_*σ*_, *g*_EE_ (excitatory conductance *g*_*E*_ on excitatory neurons); *g*_IE_ (excitatory conductance *g*_*E*_ on inhibitory neurons); *g*_EI_ (inhibitory conductance *g*_*I*_ on excitatory neurons); *g*_II_ (inhibitory conductance *g*_*I*_ on inhibitory neurons). The basal firing rates (firing rates in the uniform state of the network, prior to being cued) yielded two equations from Eq (49) by setting *w*_+_ = 1. This left 4 free parameters, which were constrained by evaluating Eq (49) at 4 points as described in [97]. The basal firing rates were chosen to be fairly low to make the uniform state more stable (as in [44]). This procedure does not yield a fixed value for *g*_*σ*_, since *g*_*σ*_ is optimized for and is not set as a target value. We thus iterated the following until a solution was found with *g*_*σ*_ ≈ 0.5: a) change the width of the recurrent weights *w*_*σ*_; b) optimize network parameters as described here; c) optimize the expected bump shape for the new network parameters to predict *g*_*σ*_. The resulting parameter values are given in Table in S2 Table.

### Frozen noise

#### Random and heterogeneous connectivity

Introducing random connectivity, we replace the recurrent weights in Eq (36) by:

$$\begin{array}{ccc} w_{ij}^{\text{EE}} & \to & [w_{ij}^{\text{EE}}+\Delta_{ij}^{w}]\frac{p_{ij}}{p}. \end{array}$$ (50)

Here, *p*_*ij*_ ∈ {0, 1} are Bernoulli variables, with *P*(*p*_*ij*_ = 1) = *p*, where the connectivity parameter *p* ∈ (0, 1] controls the overall sparsity of recurrent excitatory connections. For *p* = 1 the entire network is all-to-all connected. Additionally, we provide derivations for additive synaptic heterogeneities $\Delta_{ij}^{w}=\eta_{ij}\sigma_{w}$ (as in [38]), where {*η*_*ij*_|1 ≤ *i*, *j* ≤ *N*_*E*_} are independent, normally distributed random variables with zero mean and unit variance. We did not investigate this type of heterogeneity in the main text, since increasing *σ*_*w*_ lead to a loss of the attractor state before creating large enough directed drifts to be comparable to the other sources of frozen noise considered here—most of the small effects were “hidden” behind diffusive displacement [85]. Nevertheless, we included this case in the analysis here for completeness.

Let the center position of the bump be $\varphi_{k}=k\frac{2\pi }{N}-\pi$ (for 0 ≤ *k* < *N*). Subject to the perturbed weights, the recurrent steady-state excitatory input *J*_*i*_(*φ*_*k*_) Eq (36) to any excitatory neuron can be written as the unperturbed input *J*_0,*i*_(*φ*_*k*_) plus an additional input $J_{i}^{\text{struct}}\left(\varphi_{k}\right)$ arising from the perturbed connectivity. Note that the synaptic steady-state activations *s*_0,*j*_(*φ*_*k*_) change with varying bump centers—in the following, we denote $s_{0,j}^{k}\equiv s_{0,j}\left(\varphi_{k}\right)$:

$$\begin{array}{cc} J_{i}\left(\varphi_{k}\right) & =\frac{1}{N_{E}}\sum_{j}[w_{ij}^{\text{EE}}+\Delta_{ij}^{w}]\frac{p_{ij}}{p}s_{0,j}^{k}\\ & =\frac{1}{N_{E}}\frac{1}{p}[\sum_{j}[w_{ij}^{\text{EE}}+\Delta_{ij}^{w}]s_{0,j}^{k}-\sum_{j}[w_{ij}^{\text{EE}}+\Delta_{ij}^{w}](1-p_{ij})s_{0,j}^{k}]\\ & =\underset{J_{0,i}\left(\varphi_{k}\right)}{\underset{︸}{\frac{1}{N_{E}}\sum_{j}w_{ij}^{\text{EE}}s_{0,j}^{k}}}+\underset{J_{i}^{\text{struct}}\left(\varphi_{k}\right)}{\underset{︸}{\frac{1}{N_{E}}\frac{1}{p}[\sum_{j}[w_{ij}^{\text{EE}}+\Delta_{ij}^{w}]p_{ij}s_{0,j}^{k}-p\sum_{j}w_{ij}^{\text{EE}}s_{0,j}^{k}]}}. \end{array}$$

Note that *J*_0,*i*_(*φ*_*k*_) is an index-shifted version of the steady-state input: *J*_0,*i*_(*φ*_*k*_) = *J*_0,*i*−*k*_. However, such a relation does not hold for $J_{i}^{\text{struct}\;}\left(\varphi_{k}\right)$, since the random numbers *p*_*ij*_ will change the resulting value for varying center positions.

We calculate the firing rate perturbations *δϕ*_*i*_(*φ*_*k*_) resulting from the additional input by a linear expansion around the steady-state firing rates *ϕ*_0,*i*_(*φ*_*k*_) → *ϕ*_0,*i*_(*φ*_*k*_) + *δϕ*_*i*_(*φ*_*k*_). These evaluate to:

$$\begin{array}{ll} \delta \phi_{i}\left(\varphi_{k}\right) & =\frac{dF}{dJ}{|}_{J0,i}\left(\varphi_{k}\right)\cdot J_{i}^{\text{struct}\;\;}\left(\varphi_{k}\right)\\ & =\phi_{i,0}^{\prime }\left(\varphi_{k}\right)\cdot J_{i}^{\text{struct}}\left(\varphi_{k}\right). \end{array}$$ (51)

See *Derivatives of the rate prediction* for the derivation of the function $\frac{dF}{dJ}\left(J_{0,i}\right)$ for the spiking network used in the main text.

In the sum of Eq (7), we keep the firing rate profile ${\vec{\phi }}_{0}$ centered at *φ*_0_ while calculating the drift for varying center positions. To accommodate the shifted indices resulting from moving center positions, we re-index the summands to yields the perturbations *ϕ*_0,*i*_ → *ϕ*_0,*i*_ + Δ*ϕ*_*i*_(*φ*_*k*_) used there:

$$\begin{array}{c} \Delta \phi_{i}\left(\varphi_{k}\right)=\phi_{i,0}^{\prime }\cdot J_{i+k}^{\text{struct}}\left(\varphi_{k}\right). \end{array}$$ (52)

#### Heterogeneous leak reversal potentials

We further investigated random distributions of the leak reversal potential *V*_L_. These are implemented by the substitution

$$\begin{array}{ccc} V_{\text{L}} & \to & V_{\text{L}}+\Delta_{i}^{\text{L}}, \end{array}$$ (53)

where the $\Delta_{i}^{\text{L}}$ are independent normally distributed variables with zero mean, i.e. $\langle \Delta_{i}^{\text{L}}\rangle =0mV,\langle \Delta_{i}^{\text{L}}\Delta_{j}^{\text{L}}\rangle =\sigma_{\text{L}}^{2}\delta_{ij}$. The parameter *σ*_L_ controls the standard deviation of these random variables, and thus the noise level of the leak heterogeneities.

Let $\varphi_{k}=k\frac{2\pi }{N}-\pi$ for 0 ≤ *k* < *N* be the center position of the bump. First, note that the heterogeneities $\Delta_{i}^{L}$ do not depend on the center position *φ*_*k*_, since they are single neuron properties. As in the last section, we calculate the firing rate perturbations *δϕ*_*i*_(*φ*_*k*_) resulting from the additional input by a linear expansion around the steady-state firing rates *ϕ*_0,*i*_(*φ*_*k*_) → *ϕ*_0,*i*_(*φ*_*k*_) + *δϕ*_*i*_(*φ*_*k*_):

$$\begin{array}{cc} \delta \phi_{i}\left(\varphi_{k}\right) & =\frac{dF_{i}}{d\Delta_{i}^{\text{L}}}\left(J_{i}\left(\varphi_{k}\right)\right)\cdot \Delta_{i}^{\text{L}}\\ & \equiv \frac{d\phi_{0,i}}{d\Delta_{i}^{\text{L}}}\left(\varphi_{k}\right)\cdot \Delta_{i}^{\text{L}}. \end{array}$$ (54)

Here, $\frac{dF_{i}}{d\Delta_{i}^{\text{L}}}\left(J_{i}\left(\varphi_{k}\right)\right)$ is the derivative of the input-output relation of neuron *i* in a bump centered at *φ*_*k*_, with respect to the leak perturbation. We introduced $\frac{d\phi_{0,i}}{d\Delta^{L}}\left(\varphi_{k}\right)$ as a shorthand notation for this derivative, since it is evaluated at the steady-state input *J*_*i*,0_(*φ*_*k*_). For the spiking network of the main text, this is derived in *Derivatives of the rate prediction*.

In the sum of Eq (7), we keep the firing rate profile ${\vec{\phi }}_{0}$ centered at *φ*_0_ while calculating the drift for varying center positions. As in the last section, we re-index the sum to yield the perturbations *ϕ*_0,*i*_ → *ϕ*_0,*i*_ + Δ*ϕ*_*i*_(*φ*_*k*_) used there:

$$\begin{array}{c} \Delta \phi_{i}\left(\varphi_{k}\right)=\frac{d\phi_{0,i}}{d\Delta_{i}^{\text{L}}}\cdot \Delta_{i+k}^{\text{L}}. \end{array}$$ (55)

#### Squared field magnitude

Using the equation of the drift field in Eq (7), and the firing rate perturbations Eqs (51)–(54), it is straight forward to see that for any center position *φ* the expected drift field averaged over the noise parameters is 0, since all single firing rate perturbations vanish in expectation. In the following we calculate the variance of the drift field averaged over noise realizations, which turns out to be additive with respect to the two noise sources.

We begin by calculating the correlations between frozen noises caused by random connectivity and leak heterogeneities. For the Bernoulli distributed variables *p*_*ij*_ it holds that 〈*p*_*ij*_〉 = *p*, 〈*p*_*ij*_ *p*_*lk*_〉 = *δ*_*il*_*δ*_*jk*_*p* + (1 − *δ*_*il*_*δ*_*jk*_)*p*^2^. For the other independent random variables it holds that $\langle \Delta_{i}^{L}\rangle =0mV,\langle (\Delta_{i}^{L}{)}^{2}\rangle =\sigma_{L}^{2},\langle \Delta_{ij}^{w}\rangle =0,\langle (\Delta_{ij}^{w}{)}^{2}\rangle =\sigma_{w}^{2}$. Again, the weight heterogeneities $\Delta_{ij}^{w}$ are only included for completeness—all analyses of the main text assume that *σ*_*w*_ = 0.

For the correlations between the perturbations we then know that (for brevity, we omit the dependence on the center position *φ*):

$$\begin{array}{ccc} \left\langle J_{i}^{\text{struct}}\Delta_{i}^{L}\right\rangle & = & 0\\ \left\langle J_{i}^{\text{struct}}J_{l}^{\text{struct}}\right\rangle & = & \frac{1}{N_{E}^{2}}(\sum_{j,k}s_{0,j}w_{ij}^{\text{EE}}s_{0,k}w_{lk}^{\text{EE}}\langle (\frac{p_{ij}}{p}-1)(\frac{p_{lk}}{p}-1)\rangle )\\ & & +\frac{1}{N_{E}^{2}}(\frac{1}{p^{2}}\sum_{j,k}s_{0,j}s_{0,k}\langle \Delta_{ij}^{w}\Delta_{lk}^{w}\rangle \langle p_{ij}p_{lk}\rangle )\\ & = & \frac{1}{N_{E}^{2}}(\sum_{j}s_{0,j}^{2}(w_{ij}^{\text{EE}}{)}^{2}(\frac{1}{p}-1)+\frac{1}{p}\sum_{j}s_{0,j}^{2}\sigma_{w}^{2})\delta_{il}. \end{array}$$

Starting from Eq (7), we use as a firing rate perturbation the sum of firing rate perturbations from both Eqs (51) and (54). With the pre-factor $C_{i}=\frac{dJ_{0,i}}{d\varphi }\frac{1+\tau_{u}\phi_{0,i}(U\tau_{u}\phi_{0,i}+2)}{(U\phi_{0,i}(\tau_{u}\tau_{x}\phi_{0,i}+\tau_{u}+\tau_{x})+1){}^{2}}$, the expected squared field averaged over ensemble of frozen noises is then:

$$\begin{array}{ll} {\langle A{\left(\varphi \right)}^{2}\rangle }_{\text{frozen}} & =\frac{1}{S^{2}}\sum_{i,j}C_{i}(\varphi )C_{j}(\varphi ){\langle \left(\phi_{0,i^{\prime }}(\varphi )J_{i}^{\text{struct}}(\varphi )+\frac{d\phi_{0,i}}{d\Delta_{L}}(\varphi )\Delta_{i}^{L}\right)\cdot \left(\phi_{0,j}^{\prime }(\varphi )J_{j}^{\text{struct}}(\varphi )+\frac{d\phi_{j}}{d\Delta_{L}}(\varphi )\Delta_{j}^{L}\right)\rangle }_{\text{frozen}}\\ & =\frac{1}{S^{2}}\sum_{i}C_{i}^{2}(\varphi )\cdot [\frac{{\left(\phi_{0,i^{\prime }}(\varphi )\right)}^{2}}{N_{E}^{2}}\left(\left(\frac{1}{p}-1\right)\sum_{j}{\left(s_{0,j}(\varphi )\right)}^{2}{\left(w_{ij}^{\text{EE}}\right)}^{2}+\frac{1}{p}\sum_{j}s_{0,j}^{2}(\varphi )\sigma_{w}^{2}\right)+{\left(\frac{d\phi_{0,i}}{d\Delta_{L}}(\varphi )\right)}^{2}\sigma_{L}^{2}]. \end{array}$$ (56)

One can see directly that the two last terms are invariant under shifts of the bump center *φ*, since these introduce symmetric shifts of the indexes *i*. Similarly, it is easy to see that the first term is also invariant. Let *φ*′ be shifted to the right by one index from *φ*. It then holds that:

$$\begin{array}{cc} & \underset{i}{\sum }C_{i}^{2}\left(\varphi^{\prime }\right)\left(\frac{{\left(\phi_{0,i}^{\prime }\left(\varphi^{\prime }\right)\right)}^{2}}{N_{E}^{2}}\left(\frac{1}{p}-1\right)\underset{j}{\sum }{\left(s_{0,j}\left(\varphi^{\prime }\right)\right)}^{2}{\left(w_{ij}^{\text{EE}}\right)}^{2}\right)\\ = & \underset{i}{\sum }C_{i-1}^{2}\left(\frac{{\left(\phi_{0,i-1}^{\prime }\left(\varphi \right)\right)}^{2}}{N_{E}^{2}}\left(\frac{1}{p}-1\right)\underset{j}{\sum }{\left(s_{0,j-1}\left(\varphi \right)\right)}^{2}{\left(w_{ij}^{\text{EE}}\right)}^{2}\right)\\ = & \underset{i}{\sum }C_{i-1}^{2}\left(\frac{{\left(\phi_{0,i-1}^{\prime }\left(\varphi \right)\right)}^{2}}{N_{E}^{2}}\left(\frac{1}{p}-1\right)\underset{j}{\sum }{\left(s_{0,j}\left(\varphi \right)\right)}^{2}{\left(w_{i-1,j}^{\text{EE}}\right)}^{2}\right). \end{array}$$

The final equation holds since, in ring-attractor networks, $w_{ij}^{\text{EE}}$ consists of index-shifted rows of the same vector (see e.g. *Network connectivity* for the spiking network weights).

In summary, 〈*A*(*φ*)^2^〉_frozen_ will evaluate to the same quantity 〈*A*^2^〉_frozen_ for all center positions *φ*. In the main text, we use this fact to estimate 〈*A*^2^〉_frozen_ from simulations, by additionally averaging over the all center positions and interchanging the ensemble and positional averages:

$$\begin{array}{c} {\langle A^{2}\rangle }_{\text{frozen}}=\frac{1}{N_{E}}\underset{k}{\sum }{\langle A{\left(\varphi_{k}\right)}^{2}\rangle }_{\text{frozen}}={\langle \frac{1}{N_{E}}\underset{k}{\sum }A{\left(\varphi_{k}\right)}^{2}\rangle }_{\text{frozen}}. \end{array}$$

Thus, we can compare the value of 〈*A*^2^〉_frozen_ to the mean squared drift field over all center positions, averaged over instantiations of noises.

#### System size scaling

Generally, sums over the discretized intervals [−*π*, *π*) as they appear in Eqs (5) and (7) will scale with the number *N* chosen for the discretization of the positions on the continuous ring $\varphi \left(i\right)=\frac{i}{N}2\pi -\pi$. Consider two discretizations of the ring, partitioned into *N*_1_ and *N*_2_ uniformly spaced bins of width $\frac{2\pi }{N_{1}}$ and $\frac{2\pi }{N_{2}}$. We can then approximate integrals over any continuous (Riemann integrable) function *f* on the ring by the two Riemann sums:

$$\begin{array}{c} \frac{2\pi }{N_{1}}\sum_{i=0}^{N_{1}-1}f\left(\varphi_{1,i}\right)\approx \int_{-\pi }^{\pi }f\left(\varphi \right)d\varphi \approx \frac{2\pi }{N_{2}}\sum_{i=0}^{N_{2}-1}f\left(\varphi_{2,i}\right), \end{array}$$ (57)

where, $i\frac{2\pi }{N_{1}}\leq \varphi_{1,i}<(i+1)\frac{2\pi }{N_{1}}$ (for *N*_2_ and *φ*_2,*i*_ analogously) are points in the bins [98].

Numerical quantities for the results of the main text have been calculated for *N*_*E*_ = 800. In the following we denote all of these quantities with an asterisk (*). To generalize these results to arbitrary system size *N*, we replace sums over *N* bins by scaled sums over *N*_*E*_ bins using the relation Eq (57):

$$\begin{array}{c} \sum_{i=0}^{N-1}\to \frac{N}{N_{E}}\sum_{i=0}^{N_{E}-1}. \end{array}$$

First, we find that the normalization constant scales as $S=\frac{N}{N_{E}}S^{*}$, and thus (dots indicate the summands, which are omitted for clarity) for the diffusion strength B (cf. Eq (5)):

$$\begin{array}{cc} B & =\frac{1}{S^{2}}\sum_{i=0}^{N-1}\cdots =\frac{N}{N_{E}}\frac{1}{S^{2}}\sum_{i=0}^{N_{E}-1}\cdots \\ & =\frac{N_{E}}{N}B^{*}. \end{array}$$ (58)

For the drift magnitude we turn to the expected squared drift magnitude calculated earlier (cf. Eq (56)), for which we find that (setting *σ*_*w*_ → 0 for simplicity, as throughout the main text):

$$\begin{array}{cc} {\left\langle A^{2}\right\rangle }_{\text{frozen}} & =(\frac{N_{E}}{N}{)}^{2}\frac{1}{(S*{)}^{2}}\frac{N}{N_{E}}\sum_{i=0}^{N_{E}-1}C_{i}^{2}(\frac{(\phi_{i}^{\prime }{)}^{2}}{N^{2}}(\frac{1}{p}-1)\frac{N}{N_{E}}\sum_{j=0}^{N_{E}-1}s_{j}^{2}w_{ij}^{2}+(\frac{d\phi_{i}}{dE_{L}}{)}^{2}\sigma_{L}^{2})\\ & =\frac{1}{(S*{)}^{2}}\sum_{i=0}^{N_{E}-1}C_{i}^{2}(\frac{1}{N^{2}}(\phi_{i}^{\prime }{)}^{2}(\frac{1}{p}-1)\sum_{j=0}^{N_{E}-1}s_{j}^{2}w_{ij}^{2}+\frac{N_{E}}{N}(\frac{d\phi_{i}}{dE_{L}}{)}^{2}\sigma_{L}^{2}). \end{array}$$ (59)

Note, that we could not resolve this scaling in dependence of ${\langle A^{2}\rangle }_{\text{frozen}}^{*}$, since the two sources of frozen noise (connectivity and leak heterogeneity) show different scaling with *N*.

### Numerical methods

#### Spiking simulations

All network simulations and models were implemented in the NEST simulator [99]. Neuronal dynamics are integrated by the Runge-Kutta-Fehlberg method as implemented in the GSL library [100] (gsl_odeiv_step_rkf45)—this forward integration scheme is used in the NEST simulator for all conductance-based models (at the time of writing). The short-term plasticity model is integrated exactly, based on inter-spike intervals. Code for network simulations is available at https://github.com/EPFL-LCN/pub-seeholzer2018.

#### Simulation protocol

In all experiments (except those involving bi-stability, see below) spiking networks were simulated for a transient initial period of *t*_initial_ = 500*ms*. To center the network in an attractor state at a given angle −*π* ≤ *φ* < *π*, we gave an initial cue signal by stimulating neurons (0.2 ⋅ *N*_*E*_ neurons for networks with facilitation parameter *U* > 0.1 and 0.18 ⋅ *N*_*E*_ neurons for *U* ≤ 0.1) centered at *φ* by strong excitatory input mediated by additional Poisson firing onto AMPA receptors (0.5*s* at 3kHz followed by 0.5*s* at 1.5kHz) with connections scaled down by a factor of *g*_signal_ = 0.5. The external input ceased at *t* = *t*_off_ = 1.5*s*. For simulations to estimate the diffusion we simulated until *t*_max_ = 15*s*, yielding 13.5*s* of delay activity after the cue offset. For simulations to estimate drift we set *t*_max_ = 8*s*, yielding 6.5*s* of delay activity after the cue offset.

For simulations exploring the bi-stability between the uniform state and a bump state (Fig 1B1), we added an additional input prior to the spontaneous state. We stimulated simultaneously 20 excitatory neurons around 4 equally spaced cue points each (80 neurons in total, 500ms, 1.5kHz, AMPA connections scaled by a factor *g*_signal_ = 2). This was applied to settle networks into the uniform state more stably—without this perturbation, networks sometimes approached the bump state after being uniformly initialized. In both figures, we show population activity only after this initial stimulus was applied.

#### Estimation of centers and mean bump shapes

To estimate centers of bump states, simulations were run until *t* = *t*_max_ and spikes were recorded from the excitatory population and converted to firing rates by convolving them with an exponential kernel (*τ* = 100*ms*) [101] and then sampled at resolution 1ms. This results in vectors of firing rates *ν*_*j*_(*t*), 0 ≤ *j* ≤ *N*_*E*_ − 1 for every time *t*. We calculated the population center *φ*(*t*) for time *t* by measuring the phase of the first spatial Fourier coefficient of the firing rates. This is given by $\varphi \left(t\right)=\arg(\sum_{j}\text{exp}\left(i\frac{2\pi }{N_{E}}j\right)\nu_{j}\left(t\right))-\pi .$ For all analyses below, we identify *t* = 0 to be the time *t* = *t*_off_ of the initial cue.

To measure the mean bump shapes, we first rectified the vectors *ν*_*j*_(*t*) for every *t* by rotating the vector until *φ*(*t*) = 0. We then sampled the rectified firing rates starting from 1*s* after cue offset at intervals of 20ms, which were used to calculate the mean firing rates. S1 Fig shows mean rates for each simulation averaged over the ∼ 1000 repetitions performed in the diffusion estimation (below).

#### Exclusion of bump trajectories

Sometimes bump trajectories would leave the attractor state and return to the uniform state. We identified these trajectories in all experiments by identifying maximal firing rates across the population that dropped below 10Hz during the delay period. The such identified repetitions were excluded from the analyses, which occurred mostly in networks with no facilitation for *τ*_*x*_ = 150*ms*, *τ*_*u*_ = 650*ms*: at *U* = 1, we excluded 222/1000 repetitions from the diffusion estimation, while for all other *U* ≤ 0.8 at most 15/1000 were excluded. Increasing the depression time constant also lead to less stable attractor states: for *τ*_*x*_ = 200*ms*, *τ*_*u*_ = 650*ms* and *U* = 0.8, we had to exclude 250/1000 repetitions. During the simulations for drift estimation, we observed that frozen noise also leads to less stable bumps under weak facilitation for random and sparse connectivity (*p* ≪ 1) and high leak variability (*σ*_*L*_ ≫ 0).

#### Diffusion estimation

Diffusion was estimated for each combination of network parameters by simulating 1000 repetitions (10 initial cue positions, 100 repetitions each) of 13.5*s* of delay activity. Center positions *φ*_*k*_(*t*) were estimated for each repetition *k* as described above. We then calculated for each repetition the offset relative to the position at 500*ms* by Δ*φ*_*k*_(*t*) = *φ*_*k*_(*t* − 500*ms*) − *φ*_*k*_(500*ms*), effectively discarding the first 500*ms* after cue-offset. The time-dependent variance of *K* repetitions (excluding those repetitions in which the bump state was lost, see above) was then calculated as $V\left(t\right)=\frac{1}{K}\sum_{k}\Delta \varphi_{k}^{2}\left(t\right)$. The diffusion strength can then be estimated from the slope of a linear least-squares regression (using the Scipy method *scipy.stats.linregress* [102]) to the variance as a function of time: *V*(*t*) ≈ *D*_0_ + *D* ⋅ *t*, where the intercept *D*_0_ is included to account for initial transients. We estimated confidence intervals by bootstrapping [103]: sampling K elements out of the K repetitions with replacement (5000 samples) and estimating the confidence level of 0.95 by the bias corrected and accelerated bootstrap implemented in *scikits-bootstrap* [104]. As a control, we calculated confidence intervals for *D* additionally by Jackknifing: after building a distribution of estimates of D on *K* one-left-out samples of all repetitions, the standard error of the mean can be calculated and is multiplied by 1.96 to obtain the 95% confidence interval [105]—confidence intervals obtained by this method were almost indistinguishable from confidence intervals obtained by bootstrapping.

#### Drift estimation

Drift was estimated numerically for each combination of network and frozen noise parameters by simulating 400 repetitions (20 initial cue positions, 20 repetitions each) of 6.5*s* of delay activity. Centers positions *φ*_*k*_(*t*) were estimated for all *K* repetitions (excluding those repetitions in which the bump state was lost, see above) as explained above. We then computed displacements in time by computing a set of discrete differences

$$\begin{array}{c} \Delta \varphi_{k}=\{(\varphi_{k}[t_{0}+\left(j+1\right)dt]-\varphi [t_{0}+j\cdot dt])/dt\;|\;\forall j\in N_{0}:t_{0}+\left(j+1\right)dt\leq t_{\text{max}}\}, \end{array}$$

where we chose *dt* = 1.5*s* and *t*_0_ ∈ {500*ms*, 700*ms*, 900*ms*, …, 1900*ms*}. All differences are calculated with periodic boundary conditions on the circle [−*π*, *π*), i.e. the maximal difference was *π*/*dt*. We then calculated a binned mean (100 bins on the ring, unless mentioned otherwise) of differences calculated for all *K* trajectories, to approximate the drift-fields as a function of positions on the ring.

#### Mutual information measure

We are estimating the mutual information between a set of initial positions *x* ∈ [0, 2*π*) and associated final positions *y*(*x*) ∈ [0, 2*π*) of the trajectories of a continuous attractor network over a fixed delay period of *T*. For our results, we take *T* = 6.5*s*. We constructed binned and normalized histograms (with bin size *n* = 100, but see below) as approximate probability distributions of initial positions $p_{i}=p\left([i-1]\frac{2\pi }{n}\leq x<i\frac{2\pi }{n}\right)$ and all final positions $q_{i}=p\left([i-1]\frac{2\pi }{n}\leq y<i\frac{2\pi }{n}\right)$ (with bins indexed by 1 ≤ *i* ≤ *n*), as well as the bivariate probability distribution $r_{ij}=p\left([i-1]\frac{2\pi }{n}\leq x<i\frac{2\pi }{n},[j-1]\frac{2\pi }{n}\leq y\left(x\right)<j\frac{2\pi }{n}\right)$.

Using these, we can calculate the mutual information as [56, 57] $\text{MI}=\sum_{i=1}^{n}\sum_{j=1}^{n}r_{ij}\log_{2}(\frac{r_{ij}}{p_{i}q_{j}})$. Note, that the sum effectively counts only nonzero entries of *r*_*ij*_ (trajectories that started in bin *i* and ended in bin *j*): these imply that *p*_*i*_ ≠ 0 (a trajectory started in bin *i*) and *q*_*j*_ ≠ 0 (a trajectory ended in bin *j*), which makes the sum well defined. Although the value of MI depends on the number of bins *n*, in Figs 5 and 6 we normalize MI to that of the reference network (*U* = 1, no frozen noise, see *Short-term plasticity controls memory retention*), which leaves the resulting plot nearly invariant under a change of bin numbers.

#### Numerical integration of Langevin equations

Numerically integration of the homogeneous Langevin equations (Eq (4)) describing drift and diffusion of bump positions *φ* ∈ [−*π*, *π*) (with circular boundary conditions) has been implemented as a *C* extension in Cython [106] to the Python language [107]. Since the drift fields *A*(*φ*) are estimated on a discretization of the interval [−*π*, *π*) into *N* bins, we first interpolate drift fields *A* given as *N* discretized values to obtain continuous fields—interpolations are obtained using cubic splines on periodic boundary conditions using the class *gsl_interp_cspline_periodic* of the Gnu Scientific Library [100].

For forward integration of the Langevin equation Eq (4) from time *t* = 0, we start from an initial position *φ*_0_ = *φ*(*t* = 0). Given a time resolution *dt* (unless otherwise stated we use *dt* = 0.1*s*) and a maximal time *t*_max_ we repeat the following operations until we reach *t* = *t*_max_:

$$\begin{array}{c} t\to t+dt,\\ \varphi \to \varphi +dt\cdot A\left(\varphi \right)+\sqrt{dtB}\cdot r,\\ \varphi \to ((\varphi +\pi )\;\mathrm{mod}\;2\pi )-\pi . \end{array}$$

Here, for each iteration *r* is a random number drawn from a normal distribution with zero mean and unit variance (〈*r*〉 = 0 and 〈*r*^2^〉 = 1). The last step is performed to implement the circular boundary conditions on [−*π*, *π*).

Code implementing this numerical integration scheme is available at https://github.com/EPFL-LCN/pub-seeholzer2018-langevin.

#### Distractor analysis

For the distractor analysis in Fig 7, we let 40 neurons centered at the distractor position $\varphi_{D}=\frac{360^{{}^{\circ}}}{N}j-180^{{}^{\circ}}$ fire at rates increased by 20Hz, yielding a vector of firing rate perturbations Δ*ϕ*_0,*i*_ = 20Hz if |*i* − *j*| ≤ 20 and Δ*ϕ*_0,*i*_ = 0Hz otherwise. The vectors Δ*ϕ*_0,*i*_ for each distractor position *φ*_*D*_ are then used in Eq (7) to calculate the corresponding drift fields. To calculate the final position *φ*_1_ after 250ms of presenting the distractor, we generate 1000 trajectories starting from *φ*_0_ = 0 by integrating the Langevin equation Eq (4) for 250ms (*dt* = 0.01), the final positions of which are used to measure mean and standard deviation of *φ*_1_. For the broader bump in Fig 7D, we stretched (and interpolated) the firing rates *ϕ*_0_ as well as the associated vectors *J*_0_ and $\phi_{0}^{\prime }$ along the x-axis to obtain vectors for bumps of the desired width, and then re-calculated the values of $\frac{dJ_{0}}{d\varphi }$.

## Supporting information

S1 FigSpiking networks produce similar stable firing rate profiles across parameters.For each choice of short-term plasticity parameters *U*, *τ*_*u*_, and *τ*_*x*_, we tuned the recurrent conductances (*g*_EE_, *g*_EI_, *g*_IE_, *g*_II_) and the width *σ*_*w*_ of the distance-dependent weights (cf. Eq (32)) such that the “bump” shape of the stable firing rate profile is close to a generalized Gaussian $\nu (\theta )=g_{0}+g_{1}\text{exp}(-[\frac{\left|\theta \right|}{g_{\sigma }}{]}^{g_{r}})$ with parameters *g*_0_ = 0.1Hz, *g*_1_ = 40.0Hz, *g*_*σ*_ = 0.5, *g*_*r*_ = 2.5. See *Optimization of network parameters* in Materials and methods for details, S2 Table. for parameter values after tuning, and S1 Table. for parameters that stay constant. **A** After tuning, the resulting firing rate profiles for different parameter values of *U* and *τ*_*u*_ are very similar. Averaged mean firing rates in bump state, measured from ∼ 1000 spiking simulations. **A1-A3** Remaining slight parameter-dependent changes of bump shapes, measured by fitting the generalized Gaussian *ν*(*θ*) to the measured firing rate profiles displayed in A. **A1** Top firing rate *g*_1_. **A2** Half-width parameter *g*_*σ*_. **A3** Sharpness parameter *g*_*r*_. **B and B1-B3** Same as in A and A1-A3, for additional variation of the depression time scale *τ*_*x*_.(TIF)

S2 FigTheoretical prediction of diffusion strength as a function of STP parameters.All color values display diffusion magnitude estimated from B in Eq (4) with bump shape estimated from the reference network (*U* = 1, *τ*_*x*_ = 150ms, compare Fig 3B and 3C, dashed lines). Units of color values are $\frac{{\text{idx}}^{2}}{s}$ with values of level lines as indicated. **A** Diffusion as function of facilitation *U* and depression time constant *τ*_*x*_. Facilitation time constant was *τ*_*u*_ = 650*ms*. **B** Diffusion as function of facilitation *U* and facilitation time constant *τ*_*u*_. Depression time constant was *τ*_*x*_ = 150*ms*. **C** Diffusion as function of depression time constant *τ*_*x*_ and facilitation time constant *τ*_*u*_. Facilitation U was *U* = 0.5.(TIF)

S3 Fig Comparison of theoretically predicted fields to simulations. **A** Averaged root mean square error (RMSE) between predicted fields (Eq (7)) and fields extracted from simulations (mean over 18-20 networks, error bars show 95% confidence of the mean). Both frozen noise parameters (*σ*_*L*_ and 1 − *p*) are plotted on the same x-axis. **B** Normalized RMSE: each RMSE is normalized by the range (max − min) of the joint data of simulated and predicted fields it is calculated on. Colors as in A. **C** Average RMSE (same data as in A) plotted as a function of the mean expected field magnitude (estimated separately for each network, then averaged). Colors as in A. **D** Worst (top) and best (bottom) match between predicted field (blue line) and field extracted from simulations (black line) of the group with the largest mean RMSE in panels A, C (*U* = 1, 1 − *p* = 0.75). Shaded areas show 1 standard deviation of points included in the binned mean estimate (100 bins) of the extracted field.
(TIF)

S4 Fig Mutual information normalized to compare slopes. Same data as in Fig 5B, but MI is normalized to the average MI of each spiking network without heterogeneities (leftmost dot for each green, orange, and blue group of curves/dots), making explicitly visible the change in slope of the drop-off as heterogeneity parameters are increased. Dashed lines connect the means, for visual guidance.
(TIF)

S5 Fig Theoretical predictions of working memory stability. All panels show theoretically predicted expected displacement over 1 second (Eq (11)) for networks with random and sparse connections (*p* = 0.12) and leak reversal potential heterogeneity (*σ*_*L*_ = 1.7*mV*). White lines show displacement contour lines for 1, 2 and 5deg. **A** Displacement as a function of the facilitation time constant *τ*_*u*_ and facilitation *U* for *τ*_*x*_ = 150*ms* and *N* = 5000.**B** Displacement as a function of system size and facilitation *U* for *τ*_*x*_ = 150*ms* and *τ*_*u*_ = 650*ms*. **C-D** Displacement as a function of depression time constant *τ*_*x*_ and facilitation *U* for *N* = 5000 (C) and *N* = 20000 (D). In both panels *τ*_*u*_ = 650*ms*.
(TIF)

S6 FigDependence of diffusion strength B on shape parameters.Diffusion was calculated from Eq (5) with bump solutions $\phi_{0}=g_{1}\text{exp}\left(-{\left|\frac{x}{g_{\sigma }}\right|}^{g_{r}}\right)$. The values of $\frac{dJ_{0}}{d\varphi }$ and $\phi_{0}^{\prime }$ were calculated by fitting and extrapolating (linearly, for *ϕ*_0_ > 40.31Hz) curves $\phi_{0}\to \phi_{0}^{\prime }$ and *ϕ*_0_ → *J*_0_ that were obtained from the numerical values extracted for *g*_1_ = 40.31Hz, *g*_*σ*_ = 0.51 by theory (see *Firing rate approximation* in Materials and methods). Thus, any nonlinearity or saturation of the inputs and input-output relation for *ϕ*_0_ > 40.31Hz was not included. This approximate analysis shows that the major dependence of the diffusion expected in the system is on the bump width *g*_*σ*_, although a minor dependence on *g*_1_ is seen.(TIF)

S7 Fig Short-term plasticity does not affect spiking statistics. Mean firing rate, coefficient of variation of the inter-spike interval distribution (CV), and local CV (*CV*_2_ [92]) for two attractor networks with different STP parameters. All measures were computed on spike-trains measured over a period of 4*s*, recorded 500*ms* after offset of the external input which was centered at angle 0. Across STP parameters, networks display similarly reduced CVs for increased mean firing rates, leading to large CVs for neurons located in the flanks of the firing rate profile and low CVs for neurons located near the center. **A** Networks with large diffusion coefficient (*U* = 0.8, *τ*_*u*_ = 650*ms*, *τ*_*x*_ = 200*ms*) that underwent non-stationary diffusion during the recording of spikes: the measured mean firing rates (gray line) differ visibly from the firing rates estimated after centering the firing rate distribution at each point in time. Due to this non-stationarity, CVs at intermediate firing rates appear elevated, while the local CV (*CV*_2_) shows values close to stationary networks (see B). **B** The same network as in *A*, with strong facilitation (*U* = 0.1). Reduced diffusion leads to a nearly stationary firing rate profile, and coincident CV and *CV*_2_ measures.
(TIF)

S8 FigDiverging normalization constant *S* for increasing depression time constants *τ*_*x*_ leads to diverging diffusion.All plots show quantities related to Eqs (5) and (7) for varying depression time constants *τ*_*x*_ and facilitation strength *U*. The coefficients $\phi_{0,i},\frac{dJ_{0,i}}{d\varphi },\phi_{0,i}^{\prime }$ appearing therein are estimated from the spiking network used in the main text with *U* = 1, *τ*_*x*_ = 150*ms*, *τ*_*u*_ = 650*ms*. **A** The normalization constant *S* (“Normalizer”) of Eqs (5) and (7) shows zero crossings as *τ*_*x*_ is increased beyond facilitation-dependent critical values. **B** Diffusion strength *B* of Eq (5) without the normalization constant (equal to *B* ⋅ *S*^2^). **C** The full diffusion strength *B* of Eq (5) shows diverging values at the same critical points of *tau*_*x*_. Color legend on the right hand side shows values of *U*.(TIF)

S1 Text Detailed mathematical derivations. (PDF)

S1 Table Parameters for spiking simulations. Parameter values are modified from [11] and [50]. For recurrent conductances see the table in S2 Table.
(PDF)

S2 Table Conductance and connectivity parameters for spiking simulations. For all networks we set *w*_+_ = 4.0. Recurrent conductance parameters are given for combinations of short-term plasticity parameters according to the following notation. *g*_EE_: excitatory conductance *g*_*E*_ on excitatory neurons; *g*_IE_: excitatory conductance *g*_*E*_ on inhibitory neurons; *g*_EI_: inhibitory conductance *g*_*I*_ on excitatory neurons; *g*_II_: inhibitory conductance *g*_*I*_ on inhibitory neurons.
(PDF)
