# Supplementary figures and images for: Stability of working memory in continuous attractor networks under the control of short-term plasticity
Source: PLoS Comput Biol. 2019 Apr 19;15(4):e1006928. doi: 10.1371/journal.pcbi.1006928 (PMC6493776; doi:10.1371/journal.pcbi.1006928)

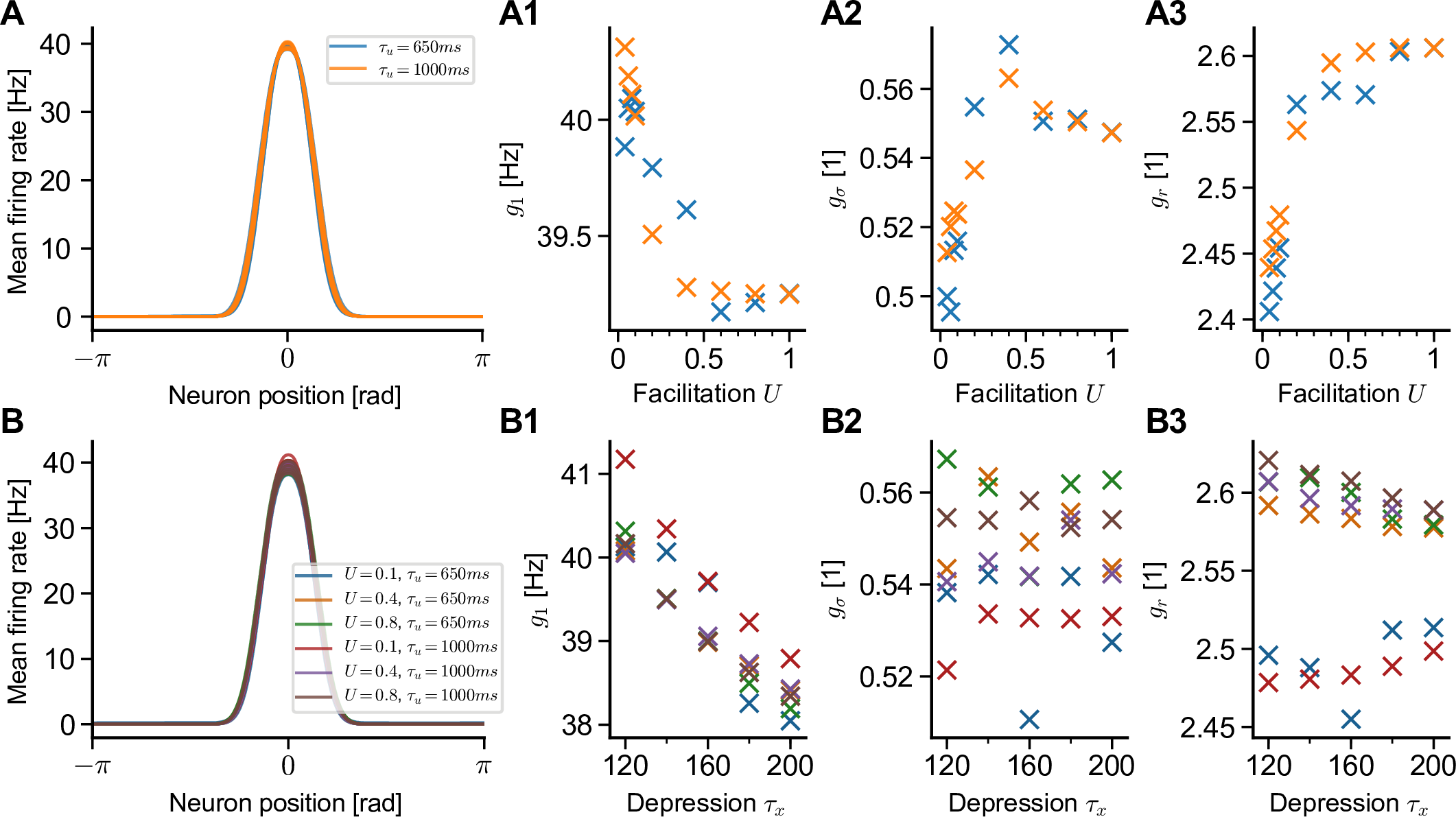

Supplement: S1 Fig — For each choice of short-term plasticity parameters U, τu, and τx, we tuned the recurrent conductances (gEE, gEI, gIE, gII) and the width σw of the distance-dependent weights (cf. Eq (32)) such that the “bump” shape of the stable firing rate profile is close to a generalized Gaussian ν(θ)=g0+g1exp(−[|θ|gσ]gr) with parameters g0 = 0.1Hz, g1 = 40.0Hz, gσ = 0.5, gr = 2.5. See Optimization of network parameters in Materials and methods for details, S2 Table. for parameter values after tuning, and S1 Table. for parameters that stay constant. A After tuning, the resulting firing rate profiles for different parameter values of U and τu are very similar. Averaged mean firing rates in bump state, measured from ∼ 1000 spiking simulations. A1-A3 Remaining slight parameter-dependent changes of bump shapes, measured by fitting the generalized Gaussian ν(θ) to the measured firing rate profiles displayed in A. A1 Top firing rate g1. A2 Half-width parameter gσ. A3 Sharpness parameter gr. B and B1-B3 Same as in A and A1-A3, for additional variation of the depression time scale τx. (TIF) [file pcbi.1006928.s001.tif]

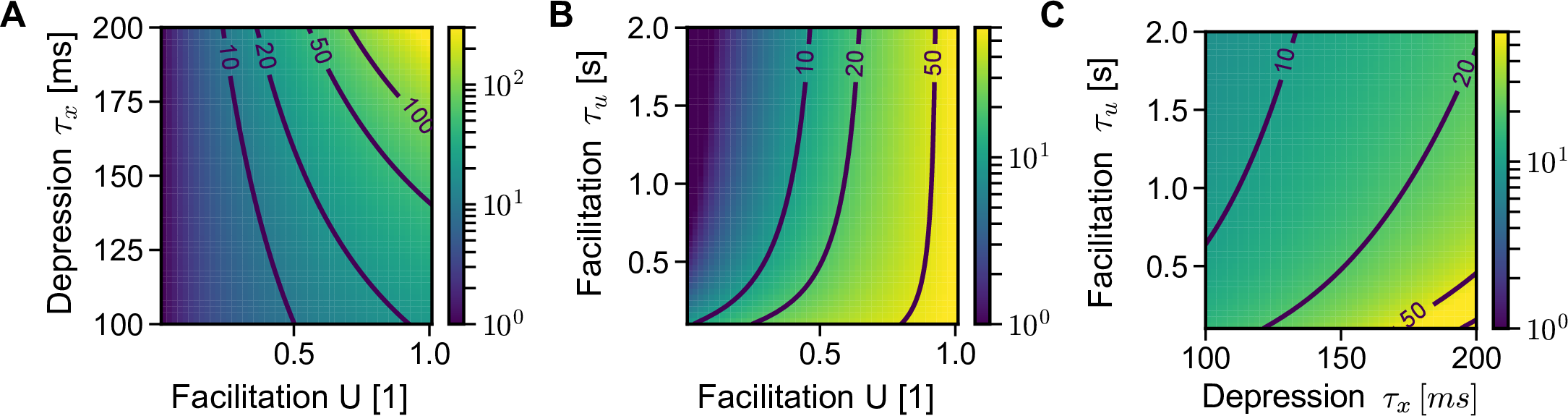

Supplement: S2 Fig — All color values display diffusion magnitude estimated from B in Eq (4) with bump shape estimated from the reference network (U = 1, τx = 150ms, compare Fig 3B and 3C, dashed lines). Units of color values are idx2s with values of level lines as indicated. A Diffusion as function of facilitation U and depression time constant τx. Facilitation time constant was τu = 650ms. B Diffusion as function of facilitation U and facilitation time constant τu. Depression time constant was τx = 150ms. C Diffusion as function of depression time constant τx and facilitation time constant τu. Facilitation U was U = 0.5. (TIF) [file pcbi.1006928.s002.tif]

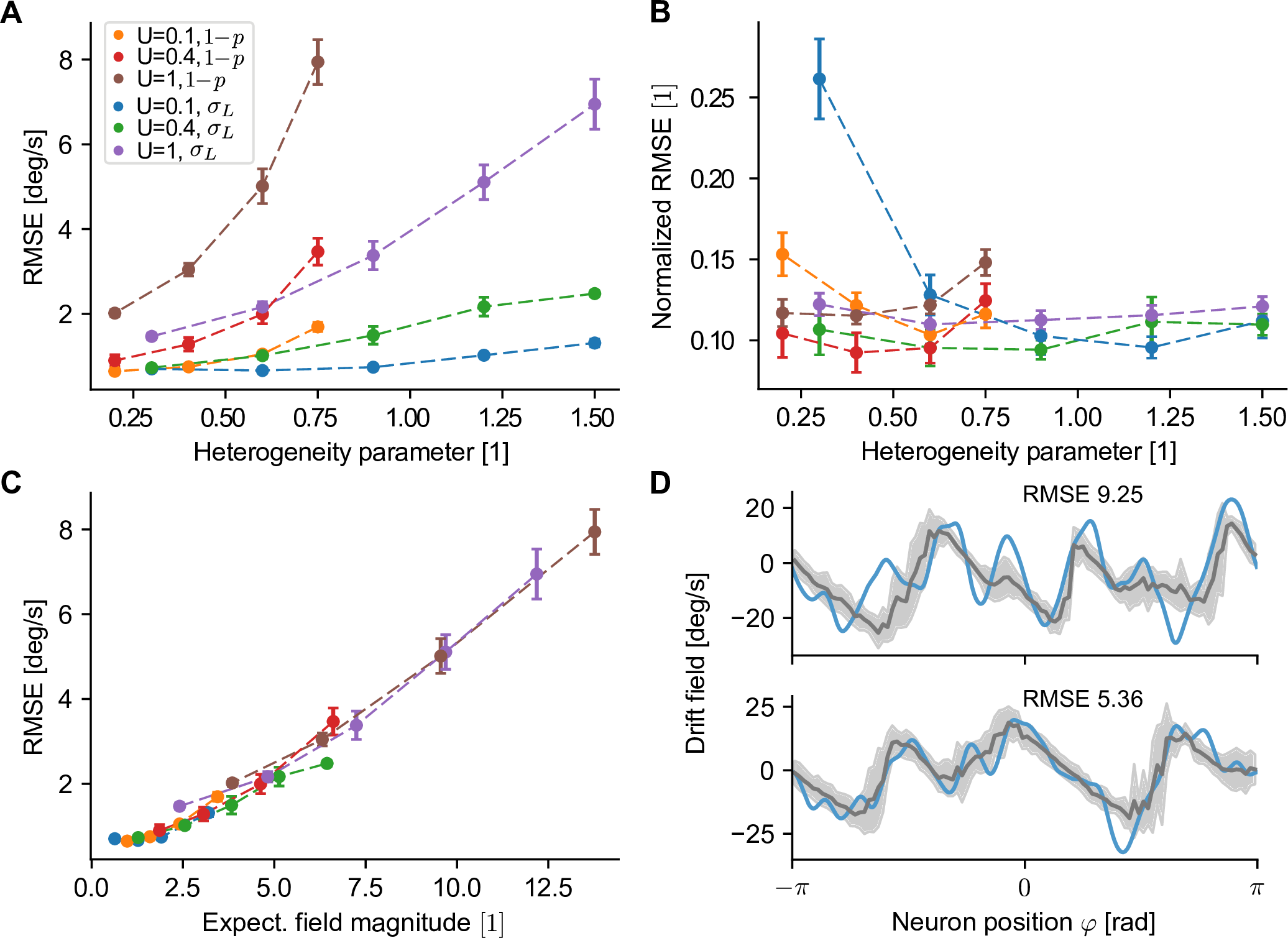

Supplement: S3 Fig — A Averaged root mean square error (RMSE) between predicted fields (Eq (7)) and fields extracted from simulations (mean over 18-20 networks, error bars show 95% confidence of the mean). Both frozen noise parameters (σL and 1 − p) are plotted on the same x-axis. B Normalized RMSE: each RMSE is normalized by the range (max − min) of the joint data of simulated and predicted fields it is calculated on. Colors as in A. C Average RMSE (same data as in A) plotted as a function of the mean expected field magnitude (estimated separately for each network, then averaged). Colors as in A. D Worst (top) and best (bottom) match between predicted field (blue line) and field extracted from simulations (black line) of the group with the largest mean RMSE in panels A, C (U = 1, 1 − p = 0.75). Shaded areas show 1 standard deviation of points included in the binned mean estimate (100 bins) of the extracted field. (TIF) [file pcbi.1006928.s003.tif]

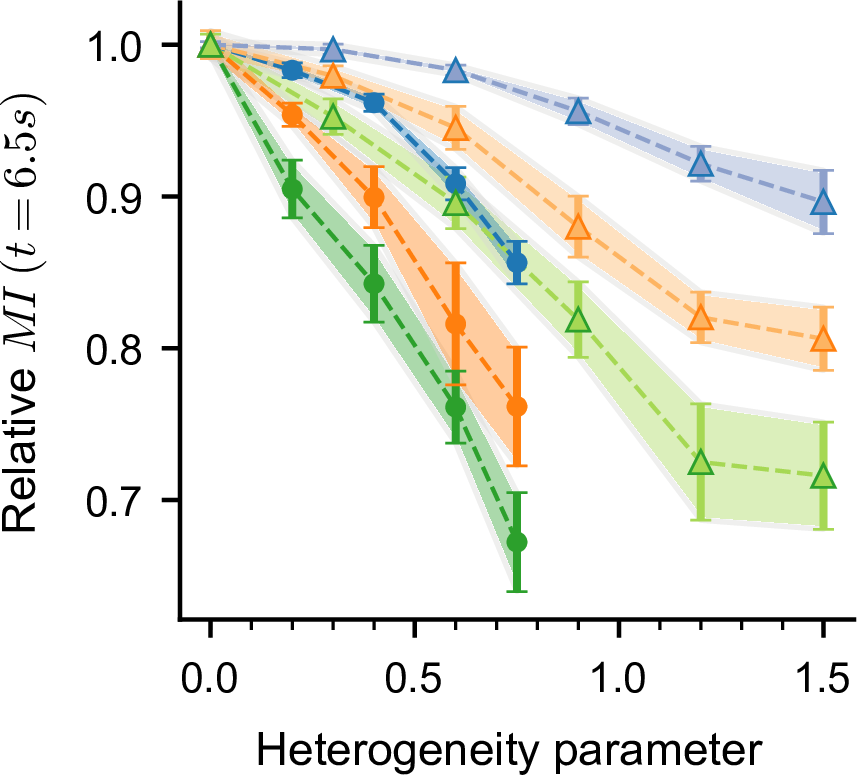

Supplement: S4 Fig — Same data as in Fig 5B, but MI is normalized to the average MI of each spiking network without heterogeneities (leftmost dot for each green, orange, and blue group of curves/dots), making explicitly visible the change in slope of the drop-off as heterogeneity parameters are increased. Dashed lines connect the means, for visual guidance. (TIF) [file pcbi.1006928.s004.tif]

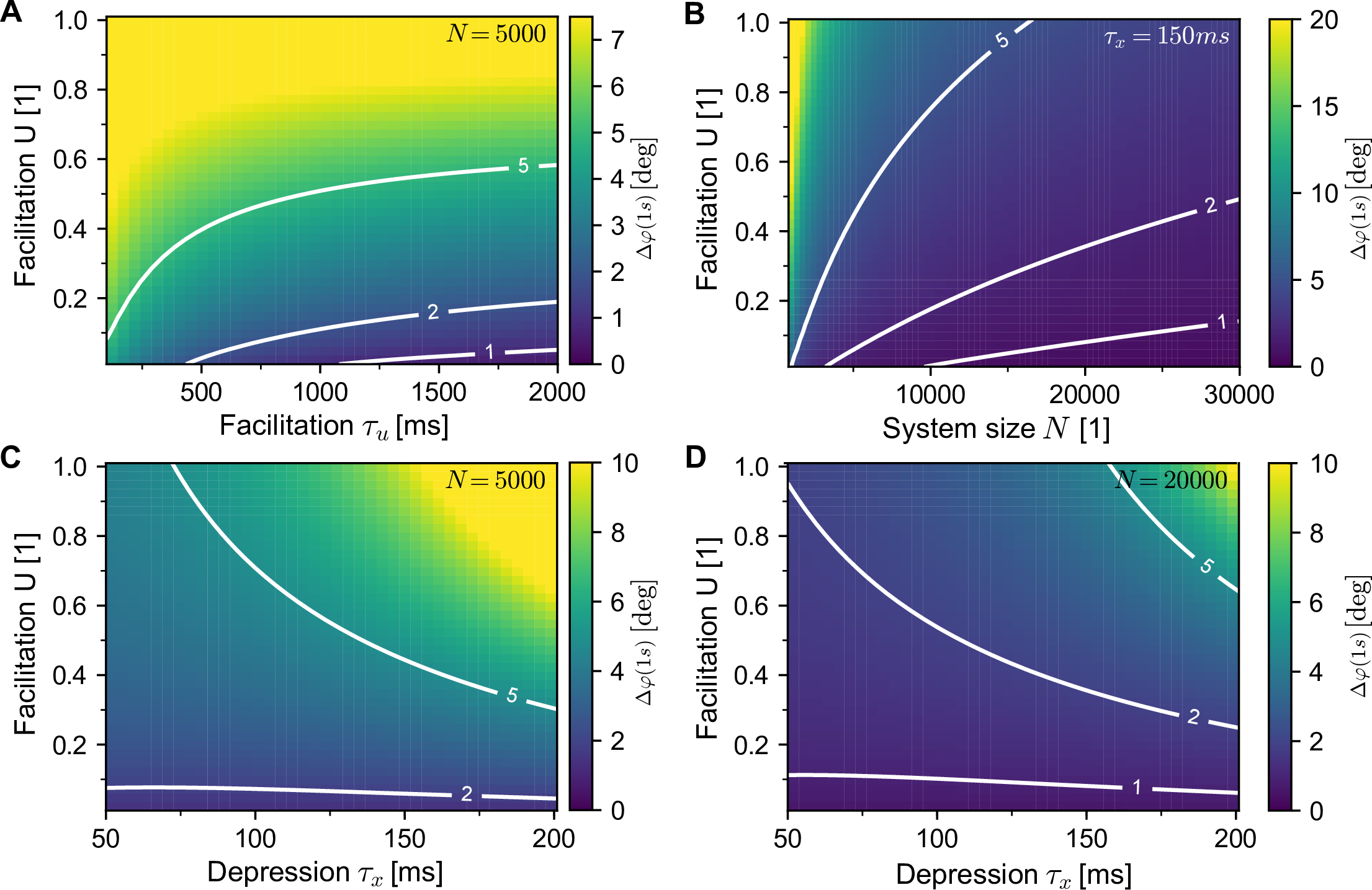

Supplement: S5 Fig — All panels show theoretically predicted expected displacement over 1 second (Eq (11)) for networks with random and sparse connections (p = 0.12) and leak reversal potential heterogeneity (σL = 1.7mV). White lines show displacement contour lines for 1, 2 and 5deg. A Displacement as a function of the facilitation time constant τu and facilitation U for τx = 150ms and N = 5000.B Displacement as a function of system size and facilitation U for τx = 150ms and τu = 650ms. C-D Displacement as a function of depression time constant τx and facilitation U for N = 5000 (C) and N = 20000 (D). In both panels τu = 650ms. (TIF) [file pcbi.1006928.s005.tif]

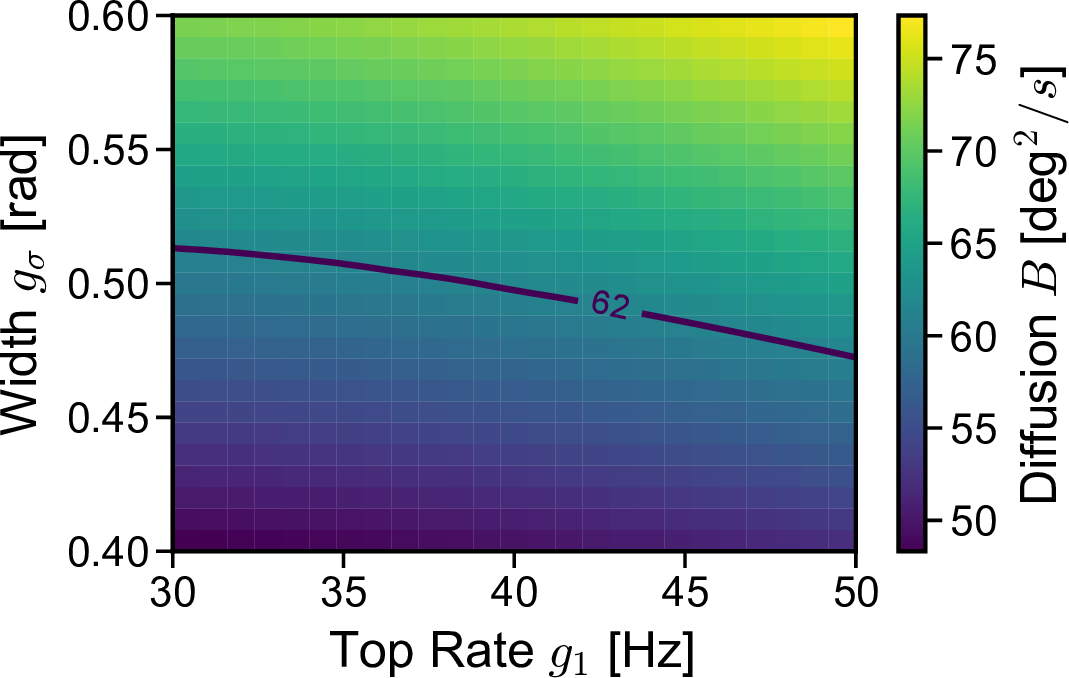

Supplement: S6 Fig — Diffusion was calculated from Eq (5) with bump solutions ϕ0=g1exp(−|xgσ|gr). The values of dJ0dφ and ϕ0′ were calculated by fitting and extrapolating (linearly, for ϕ0 > 40.31Hz) curves ϕ0→ϕ0′ and ϕ0 → J0 that were obtained from the numerical values extracted for g1 = 40.31Hz, gσ = 0.51 by theory (see Firing rate approximation in Materials and methods). Thus, any nonlinearity or saturation of the inputs and input-output relation for ϕ0 > 40.31Hz was not included. This approximate analysis shows that the major dependence of the diffusion expected in the system is on the bump width gσ, although a minor dependence on g1 is seen. (TIF) [file pcbi.1006928.s006.tif]

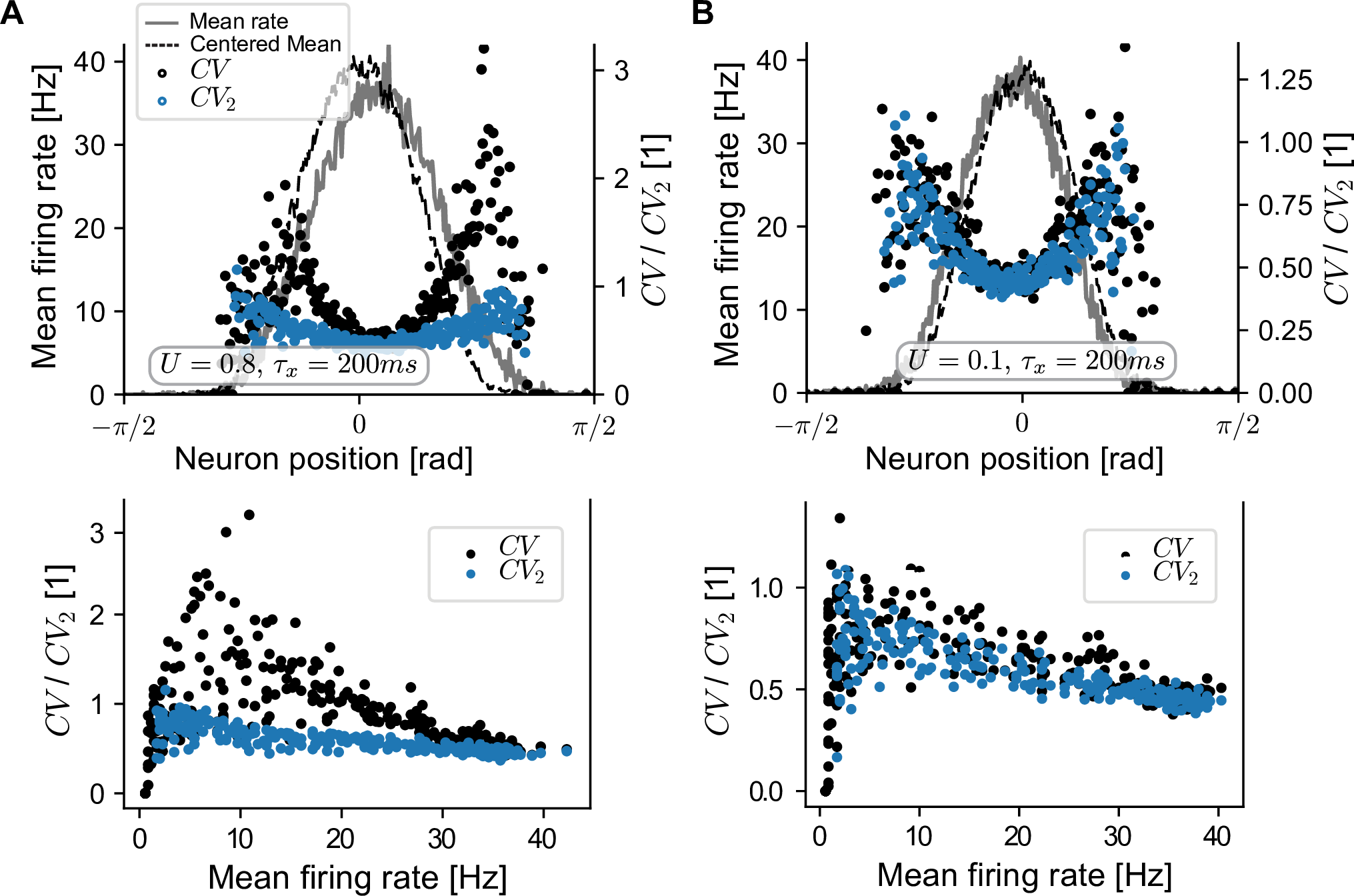

Supplement: S7 Fig — Mean firing rate, coefficient of variation of the inter-spike interval distribution (CV), and local CV (CV2 [92]) for two attractor networks with different STP parameters. All measures were computed on spike-trains measured over a period of 4s, recorded 500ms after offset of the external input which was centered at angle 0. Across STP parameters, networks display similarly reduced CVs for increased mean firing rates, leading to large CVs for neurons located in the flanks of the firing rate profile and low CVs for neurons located near the center. A Networks with large diffusion coefficient (U = 0.8, τu = 650ms, τx = 200ms) that underwent non-stationary diffusion during the recording of spikes: the measured mean firing rates (gray line) differ visibly from the firing rates estimated after centering the firing rate distribution at each point in time. Due to this non-stationarity, CVs at intermediate firing rates appear elevated, while the local CV (CV2) shows values close to stationary networks (see B). B The same network as in A, with strong facilitation (U = 0.1). Reduced diffusion leads to a nearly stationary firing rate profile, and coincident CV and CV2 measures. (TIF) [file pcbi.1006928.s007.tif]

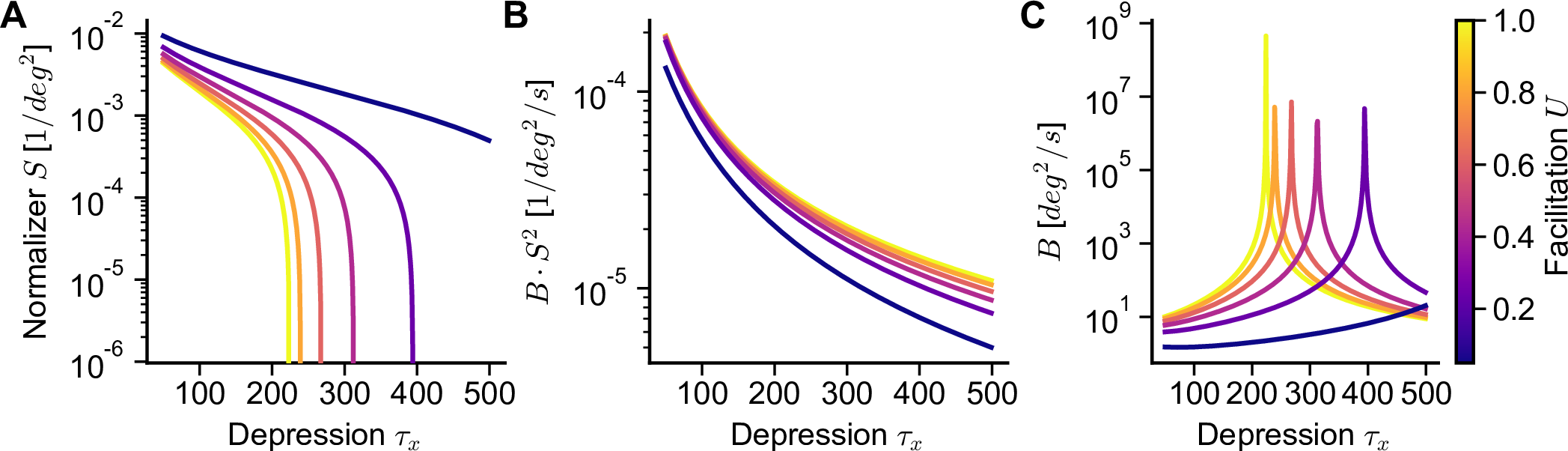

Supplement: S8 Fig — All plots show quantities related to Eqs (5) and (7) for varying depression time constants τx and facilitation strength U. The coefficients ϕ0,i,dJ0,idφ,ϕ0,i′ appearing therein are estimated from the spiking network used in the main text with U = 1, τx = 150ms, τu = 650ms. A The normalization constant S (“Normalizer”) of Eqs (5) and (7) shows zero crossings as τx is increased beyond facilitation-dependent critical values. B Diffusion strength B of Eq (5) without the normalization constant (equal to B ⋅ S2). C The full diffusion strength B of Eq (5) shows diverging values at the same critical points of taux. Color legend on the right hand side shows values of U. (TIF) [file pcbi.1006928.s008.tif]
