# Supplementary material for: Stability of working memory in continuous attractor networks under the control of short-term plasticity
Source: PLoS Comput Biol. 2019 Apr 19;15(4):e1006928. doi: 10.1371/journal.pcbi.1006928 (PMC6493776; doi:10.1371/journal.pcbi.1006928)
Supplement: S1 Text — (PDF) [file pcbi.1006928.s009.pdf]

S1 Text: Stability of working memory in continuous attractor networks under the control of short-term plasticity

Alexander Seeholzer[1], Moritz Deger[1,2], Wulfram Gerstner[1*]

1 School of Computer and Communication Sciences and School of Life Sciences, Brain Mind Institute, École Polytechnique Fédérale de Lausanne, Lausanne, Switzerland
2 Institute for Zoology, Faculty of Mathematics and Natural Sciences, University of Cologne, Cologne, Germany

* wulfram.gerstner@epfl.ch

1 Left eigenvector projects onto center positions

Here, we provide additional details to the derivation of the relation Eq (16). Let the full diagonalization[1] of the matrix K be $\bar{K} = T^{-1}KT$. Without loss of generality, let e_r be the first column of the transformation matrix T, and let e_l be the first row vector of T^{-1}, with $e_l \cdot e_r = 1$. Now, the projection of the perturbation $\delta\mathbf{y}$ onto contributions along e_r isolates displacements of the center position: the perturbation $\delta\mathbf{y} = e_r\delta\varphi$ corresponds to a shift $\varphi + \delta\varphi$ in the center position (by definition, see Eq (14)). We left multiply this relation with e_l to see that $e_l\delta\mathbf{y} = e_l e_r \delta\varphi = \delta\varphi$. Finally, we take the time derivative, which yields:

$$\dot{\delta\varphi} = e_l\dot{\delta\mathbf{y}}. \tag{S1}$$

Thus, to find an equation for the temporal dynamics of changes in the center positions, we project the system Eq (13) onto contributions along the e_r eigenspace by left multiplying by e_l. In the full matrix equations, this corresponds to rewriting the system Eq (13) as

$$T^{-1}\dot{\delta\mathbf{y}} = \bar{K}T^{-1}\delta\mathbf{y}.$$

Restricting this onto only the first dimension (the e_r eigenspace) and using Eq (S1), we find that the linear dynamics vanish (since the first entry in $\bar{K}$ is zero) and we are left with:

$$\dot{\varphi} = \dot{\delta\varphi} = e_l\dot{\delta\mathbf{y}} = 0 \cdot e_l\delta\mathbf{y}, \tag{S2}$$

where we have assumed that $\varphi(t) = \varphi(t=0) + \delta\varphi(t)$.

Thus, left-multiplying the linearized equations Eq (13) by e_l yields a differential equation for the changes $\delta\varphi$ of the center position.

2 Derivation of the left eigenvector

We will find a parametrized vector $\mathbf{y}'(\mathbf{y}) = \left(\mathbf{t}^T(\mathbf{y}), \mathbf{v}^T(\mathbf{y}), \mathbf{z}^T(\mathbf{y})\right)^T$ that for $\mathbf{y} = e_r$ fulfills the transposed eigenvalue equation of the left eigenvector:

$$K^T\mathbf{y}'(e_r) = 0. \tag{S3}$$

[1] Or Jordan normal form, if K is not fully diagonalizable. Since we know there exists a zero eigenvalue with one dimensional Eigenspace, the corresponding normal form will have a diagonal 0 entry with e_r being the corresponding eigenvector.

The system of equations resulting from $\dot{\mathbf{y}}' = K^T \mathbf{y}'$ reads (cf. Eq (13)):

$$\begin{aligned} \dot{t}_i &= -\frac{t_i}{\tau_s} + \sum_j w_{ji} u_{0,j} x_{0,j} \phi_j^{'} t_j + U \sum_j w_{ji} (1-u_{0,j}) \phi_j^{'} v_j - \sum_j w_{ji} u_{0,j} x_{0,j} \phi_j^{'} z_j && \text{(S4)} \\ \dot{v}_i &= -\frac{v_i}{\tau_u} + \phi_{0,i} x_{0,i} (t_i - z_i) - U \phi_{0,i} v_i && \text{(S5)} \\ \dot{z}_i &= -\frac{z_i}{\tau_x} + u_{0,i} \phi_{0,i} (t_i - z_i) && \text{(S6)} \end{aligned}$$

Let us first consider the system without facilitation or depression. The linearized dynamics of $\delta\mathbf{s}$ are given by the upper left $N \times N$ block K_s of Eq (13), with $U \to 1$, $u_0 \to 1, x_0 \to 1$ (which recovers the case of [1]):

$$\begin{aligned} \delta \dot{s}_i &= -\frac{1}{\tau_s} \delta s_i + \phi_{0,i}' \sum_j w_{ij} \delta s_j && \text{(S7)} \\ &\equiv K_s \delta \mathbf{s} && \text{(S8)} \end{aligned}$$

We assume that $w_{ji} = w_{ij}$, which in general holds for the symmetric synaptic connectivity of common models of continuous attractor networks (see below a concrete spiking model). The transposed block K_s^T then describes the linear dynamics of perturbations to the input variables $J_i = \sum_j w_{ij} s_j$ [1]. To see this, we differentiate $\delta J_i = \sum_j w_{ij} \delta s_j$ with respect to time, use Eq (S7), and use the symmetric connectivity, to arrive at:

$$\begin{aligned} \delta \dot{J}_i &= \sum_j w_{ij} \delta \dot{s}_j = \sum_j w_{ij} \left(-\frac{1}{\tau_s} \delta s_j + \phi_{0,j}' \delta J_j \right) \\ &= \left(-\frac{1}{\tau_s} + \sum_j w_{ji} \phi_{0,j}' \right) \delta J_j \\ &= \left(K_s^T \delta \mathbf{J} \right)_i . && \text{(S9)} \end{aligned}$$

If $\delta \dot{s}_i = 0$ for all i, which is the case if $\delta s_i = e_r$ restricted to the first N entries, we know that $\delta \dot{J}_i = \sum_j w_{ij} \delta \dot{s}_j = 0$. Thus, in this restricted case, the left eigenvector proportional to $t_i = \delta J_i$ since it fulfills Eq (S3) restricted to the first $N \times N$ block.

We now consider again the full system (with facilitation and depression), where we start with the same Ansatz for parametrization of the variables t_i [2]:

$$t_i \equiv \sum_j w_{ij} \delta s_j, \qquad \text{(S10)}$$

and continue to find variables v_i and z_i that satisfy the full equations Eq (S3).

First, we differentiate Eq (S10) with respect to time and use the linear response Eq (13) to obtain

$$\begin{aligned} \dot{t}_i &= \sum_j w_{ij} \delta \dot{s}_j = \sum_j w_{ij} \left(-\frac{\delta s_j}{\tau_s} + \phi_j^{'} u_{0,j} x_{0,j} \sum_k w_{jk} \delta s_k + \phi_{0,j} x_{0,j} \delta u_j + \phi_{0,j} u_{0,j} \delta x_j \right) \\ &= -\frac{t_i}{\tau_s} + \sum_j w_{ij} u_{0,j} x_{0,j} \phi_j^{'} t_j + \sum_j w_{ij} \phi_{0,j} x_{0,j} \delta u_j + \sum_j w_{ij} \phi_{0,j} u_{0,j} \delta x_j . && \text{(S11)} \end{aligned}$$

We then equate Eqs. (S4) and (S11), which yields the following identity:

$$\sum_j w_{ij} \phi_{0,j} \left(x_{0,j} \delta u_j + u_{0,j} \delta x_j \right) = \sum_j w_{ji} \phi_j^{'} \left(U (1-u_{0,j}) v_j - u_{0,j} x_{0,j} z_j \right) . \qquad \text{(S12)}$$

[2] This was motivated by numerical evaluations of the left eigenvector e_l of the full system, which showed that here also $t_i = \delta J_i$.

To relate the remaining variables δu_i and δx_i to the new variables v_i and z_i, we make a linear Ansatz:

$$v_i = \alpha_1 \delta u_i + \alpha_2 \delta x_i, \tag{S13}$$

$$z_i = \beta_1 \delta u_i + \beta_2 \delta x_i. \tag{S14}$$

By differentiating these equations with respect to time and equating the result to Eqs. (S5) and (S6), respectively, we find the following equations

$$-\frac{v_i}{\tau_u} + \phi_{0,i} x_{0,i}(t_i - z_i) - U\phi_{0,i} v_i = \alpha_1 \delta \dot{u}_i + \alpha_2 \delta \dot{x}_i \tag{S15}$$

$$-\frac{z_i}{\tau_x} + u_{0,i}\phi_{0,i}(t_i - z_i) = \beta_1 \delta \dot{u}_i + \beta_2 \delta \dot{x}_i \tag{S16}$$

The linear response for $\dot{u}_i$ and $\dot{x}_i$ can be obtained from Eq (13) (substituting $t_i = \sum_j w_{ij} \delta s_j$):

$$\delta \dot{u}_i = -\delta u_i \left(\frac{1}{\tau_u} - U\phi_{0,i} \right) + U(1 - u_{0,i}) \phi_i' t_i,$$

$$\delta \dot{x}_i = -\delta x_i \left(\frac{1}{\tau_x} - \phi_{0,i} \right) - x_{0,i}\phi_{0,i} \delta u_i - u_0 x_0 \phi_i' t_i.$$

By inserting these two equations and Eqs. (S13) and (S14) into Eqs. (S15) and (S16), we obtain two closed equations in $\delta u_i, \delta x_i, t_i$. By equating coefficients for $\delta u_i, \delta x_i, t_i$[3], we obtain solutions for the coefficients $\alpha_1, \alpha_2, \beta_1, \beta_2$ that fulfill these equations:

$$\alpha_1 = \frac{x_{0,i}\phi_{0,i} \left[U\tau_u \tau_x \phi_{0,i} + u_{0,i} (\tau_x - \tau_u) \right]}{U(1 - u_{0,i}) \phi_i' \left[\tau_u \tau_x \phi_{0,i} \left(U - u_{0,i}^2 \right) + u_{0,i} (\tau_x - \tau_u) \right]}$$

$$\alpha_2 = -\frac{\tau_u \tau_x u_{0,i} \phi_{0,i}^2}{\phi_i' \left[\tau_u \tau_x \phi_{0,i} \left(u_{0,i}^2 - U \right) + u_{0,i} (\tau_u - \tau_x) \right]}$$

$$\beta_1 = -\frac{\tau_u \tau_x u_{0,i} \phi_{0,i}^2}{\phi_i' \left(\tau_u \tau_x \phi_{0,i} \left(u_{0,i}^2 - U \right) + u_{0,i} (\tau_u - \tau_x) \right)}$$

$$\beta_2 = \frac{u_{0,i}\phi_{0,i} \left[\tau_u \left(\tau_x \phi_{0,i} (U - u_{0,i}) - 1 \right) + \tau_x \right]}{x_{0,i} \phi_i' \left[\tau_u \tau_x \phi_{0,i} \left(u_{0,i}^2 - U \right) + u_{0,i} (\tau_u - \tau_x) \right]}. \tag{S17}$$

A little bit of further algebra shows that these coefficients together with Eqs. (S13) and (S14) also satisfy Eq (S12), as for every j it holds that $\phi_j' \left(U(1 - u_{0,j}) v_j - u_{0,j} x_{0,j} z_j \right) = \phi_{0,j} \left(x_{0,j} \delta u_j + u_{0,j} \delta x_j \right)$.

Thus, we have found a linear parametrization

$$\mathbf{y}'(\mathbf{y})^T = \left((W\delta \mathbf{s})^T, (\alpha_1 \delta \mathbf{u} + \beta_1 \delta \mathbf{x})^T, (\alpha_2 \delta \mathbf{u} + \beta_2 \delta \mathbf{x})^T \right), \tag{S18}$$

which fulfills $\dot{\mathbf{y}}' = K^T \mathbf{y}'$. In addition, we know that if $\mathbf{y} = e_r$, then the original system dynamics vanish since $\dot{\mathbf{y}} = K\mathbf{y} = 0$. Thus, since the parametrization is linear in the original variables, it also holds that $\dot{\mathbf{y}}' = 0$, and the parametrization satisfies Eq (S3). This makes $\mathbf{y}'(e_r)^T$ *proportional* to the (unique) left eigenvector e_l of the 0-eigenvalue.

Finally, we can evaluate the vector $\mathbf{y}'(e_r)$ by using Eq (14) in Eq (S18):

$$\mathbf{y}'(e_r)^T = \left(\frac{d\mathbf{J}_0}{d\varphi}^T, \left(\alpha_1 \frac{d\mathbf{u}_0}{d\varphi} + \alpha_2 \frac{d\mathbf{x}_0}{d\varphi} \right)^T, \left(\beta_1 \frac{d\mathbf{u}_0}{d\varphi} + \beta_2 \frac{d\mathbf{x}_0}{d\varphi} \right)^T \right). \tag{S19}$$

Note, that in the first component we used that $\frac{dJ_{0,i}}{d\varphi} = \sum_j w_{ij} \frac{ds_{0,j}}{d\varphi}$.

[3] Comparing coefficients of any two of the three variables yields 4 equations, which give the same solution and satisfy the equations posed by the remaining variable.

3 Normalization constant S

In the last section we have found a vector $\mathbf{y}'^T$ proportional to the left eigenvector e_l. Since it holds that $e_l e_r = 1$, it remains to calculate the normalization constant S such this normalization condition is fulfilled by

$$e_l = \frac{1}{S}\mathbf{y}'(e_r)^T. \tag{S20}$$

First, we calculate the components of the vector e_r, Eq (14), using the steady-state values of Eq (12):

$$\frac{ds_{0,i}}{d\varphi} = \tau_s\left(u_{0,i}x_{0,i}\phi'_{0,i}\frac{dJ_{0,i}}{d\varphi} + x_{0,i}\phi_{0,i}\frac{du_{0,i}}{d\varphi} + u_{0,i}\phi_{0,i}\frac{dx_{0,i}}{d\varphi}\right), \tag{S21}$$

$$\frac{du_{0,i}}{d\varphi} = u'_{0,i}\phi'_{0,i}\frac{dJ_{0,i}}{d\varphi} = \frac{(1-U)U\tau_u}{(U\phi_{0,i}\tau_u+1)^2}\phi'_{0,i}\frac{dJ_{0,i}}{d\varphi}, \tag{S22}$$

$$\frac{dx_{0,i}}{d\varphi} = x'_{0,i}\phi'_{0,i}\frac{dJ_{0,i}}{d\varphi} = -\frac{U\tau_x\left(\phi_{0,i}\tau_u\left(U\phi_{0,i}\tau_u+2\right)+1\right)}{\left(U\phi_{0,i}\left(\tau_u+\phi_{0,i}\tau_u\tau_x+\tau_x\right)+1\right)^2}\phi'_{0,i}\frac{dJ_{0,i}}{d\varphi}, \tag{S23}$$

where we introduced the shorthand notations $u'_{0,i} = \frac{du_{0,i}}{d\phi_{0,i}}$, $x'_{0,i} = \frac{dx_{0,i}}{d\phi_{0,i}}$ and $\phi'_{0,i} = \left.\frac{d\phi_i}{dJ_i}\right|_{J_{0,i}}$. We have also used the steady-state values of Eq (12) to calculate the values of $u'_{0,i}$ and $x'_{0,i}$ by differentiating with respect to $\phi_{0,i}$.

Now, using Eq. (S20) in $e_l \cdot e_r = 1$, and plugging in Eqs. (14) and (S19), we find that:

$$\begin{aligned}
S =& \mathbf{y}'(e_r)^T \cdot e_r \\
=& \tau_s \sum_i \frac{dJ_{0,i}}{d\varphi}\left(u_{0,i}x_{0,i}\phi'_{0,i}\frac{dJ_{0,i}}{d\varphi} + x_{0,i}\phi_{0,i}\frac{du_{0,i}}{d\varphi} + u_{0,i}\phi_{0,i}\frac{dx_{0,i}}{d\varphi}\right) \qquad \text{(S24)} \\
&+ \sum_i \frac{du_{0,i}}{d\varphi}\left(\alpha_1\frac{du_{0,i}}{d\varphi} + \alpha_2\frac{dx_{0,i}}{d\varphi}\right) + \sum_i \frac{dx_{0,i}}{d\varphi}\left(\beta_1\frac{du_{0,i}}{d\varphi} + \beta_2\frac{dx_{0,i}}{d\varphi}\right) \\
=& \tau_s \sum_i \left(\frac{dJ_{0,i}}{d\varphi}\right)^2 \phi'_{0,i}\left(u_{0,i}x_{0,i} + x_{0,i}\phi_{0,i}u'_{0,i} + u_{0,i}\phi_{0,i}x'_{0,i}\right) \\
&+ \sum_i \left(\frac{dJ_{0,i}}{d\varphi}\right)^2 \phi'^2_{0,i}\left[\alpha_1 u'^2_{0,i} + \beta_2 x'^2_{0,i} + 2\alpha_2 u'_{0,i}x'_{0,i}\right] \\
=& U\sum_i \frac{\left(\frac{dJ_{0,i}}{d\varphi}\right)^2\phi'_i}{\left[U\phi_{0,i}\left(\tau_u\left(\tau_x\phi_{0,i}+1\right)+\tau_x\right)+1\right]^3} \\
&\Bigg[\tau_s\left[\tau_u\phi_{0,i}\left(U\tau_u\phi_{0,i}+2\right)+1\right]\left[U\phi_{0,i}\left(\tau_u\left(\tau_x\phi_{0,i}+1\right)+\tau_x\right)+1\right] \\
&-\phi_{0,i}\left[(U-1)\tau_u^2 + U\tau_x^2\left(\tau_u\phi_{0,i}+1\right)\left(\tau_u\phi_{0,i}\left(U\tau_u\phi_{0,i}+2\right)+1\right)\right] \qquad \text{(S25)} \\
&-\frac{(U-1)U\tau_u^2\tau_x\phi_{0,i}\left(\tau_u\phi_{0,i}+1\right)}{\left(U\tau_u\phi_{0,i}+1\right)}\Bigg], \qquad \text{(S26)}
\end{aligned}$$

which defines the normalization constant S. In the last equation we used the steady-state values of Eq (12) to calculate the values of $u'_{0,i}$ and $x'_{0,i}$ by differentiating with respect to $\phi_{0,i}$. Additionally, the coefficients of Eq (S17) were used.

4 Diffusion strength B

To calculate the correlation function of Eq (22), we first note that only terms $\langle \eta_i(t)\eta_i(t+\tau)\rangle = \delta(\tau)$ remain in expectation. Thus, we arrive at:

$$\begin{aligned}
\langle \dot{\varphi}(t)\dot{\varphi}(t+\tau)\rangle =& \langle e_l L(t) e_l L(t+\tau)\rangle \\
=& \sum_{i=1}^{n} \left(e_{l,i}^2 u_{0,i}^2 x_{0,i}^2 + e_{l,n+i}^2 U^2 (1-u_{0,i})^2 + e_{l,2n+i}^2 u_{0,i}^2 x_{0,i}^2\right) \phi_{0,i}\delta(\tau) \\
&+ 2\sum_{i=1}^{n} \left[\left(e_{l,i}e_{l,n+i} - e_{l,n+i}e_{l,2n+i}\right) U(1-u_{0,i}) - e_{l,i}e_{l,2n+i}u_{0,i}x_{0,i}\right] u_{0,i}x_{0,i}\phi_{0,i}\delta(\tau) \\
=& \frac{1}{S^2}\sum_i \left(\frac{dJ_{0,i}}{d\varphi}\right)^2 \phi_{0,i} \Big[u_{0,i}^2 x_{0,i}^2 + U^2(1-u_{0,i})^2 \phi_{0,i}'^2 \left(\alpha_1 u_{0,i}' + \alpha_2 x_{0,i}'\right)^2 \\
&\quad + u_{0,i}^2 x_{0,i}^2 \phi_{0,i}'^2 \left(\beta_1 u_{0,i}' + \beta_2 x_{0,i}'\right)^2\Big] \delta(\tau) \\
&+ \frac{2}{S^2}\sum_i \left(\frac{dJ_{0,i}}{d\varphi}\right)^2 \phi_{0,i} u_{0,i} x_{0,i} U(1-u_{0,i}) \\
&\quad \Big[\phi_{0,i}'\left(\alpha_1 u_{0,i}' + \alpha_2 x_{0,i}'\right) - \phi_{0,i}'^2 \left(\alpha_1 u_{0,i}' + \alpha_2 x_{0,i}'\right)\left(\beta_1 u_{0,i}' + \beta_2 x_{0,i}'\right)\Big] \delta(\tau) \\
&- \frac{2}{S^2}\sum_i \left(\frac{dJ_{0,i}}{d\varphi}\right)^2 \phi_{0,i} u_{0,i}^2 x_{0,i}^2 \phi_{0,i}' \left(\beta_1 u_{0,i}' + \beta_2 x_{0,i}'\right)\delta(\tau) \\
=& \frac{U^2}{S^2}\sum_i \frac{\left(\frac{dJ_{0,i}}{d\varphi}\right)^2 \phi_{0,i}\left(1 + 2\tau_u\phi_{0,i} + U\tau_u^2\phi_{0,i}^2\right)^2}{\left(U\phi_{0,i}\left(\tau_u\left(\tau_x\phi_{0,i}+1\right)+\tau_x\right)+1\right)^4}\delta(\tau) \\
\equiv& B\delta(\tau).
\end{aligned} \tag{S27}$$

In the last equation we again have used the steady-state values of Eq (12) to calculate the values of $u_{0,i}'$ and $x_{0,i}'$ by differentiating with respect to $\phi_{0,i}$. Additionally, the coefficients of Eq (S17) were used.

5 Drift term

Left-multiplying Eq. (25) by the left eigenvector e_l yields:

$$\begin{aligned}
\dot{\varphi} =& e_l \begin{pmatrix} \mathbf{x}_0\mathbf{u}_0\Delta\vec{\phi}(\varphi) \\ U(1-\mathbf{u}_0)\Delta\vec{\phi}(\varphi) \\ -\mathbf{x}_0\mathbf{u}_0\Delta\vec{\phi}(\varphi) \end{pmatrix} + \sqrt{B}\eta \\
=& \frac{1}{S}\sum_i \Bigg[\frac{dJ_{0,i}}{d\varphi}x_{0,i}u_{0,i} + \left(\alpha_1\frac{du_i}{d\varphi} + \alpha_2\frac{dx_i}{d\varphi}\right) U\left(1-u_{0,i}\right) \\
&\quad - \left(\beta_1\frac{du_i}{d\varphi} + \beta_2\frac{dx_i}{d\varphi}\right) x_{0,i}u_{0,i}\Bigg]\Delta\phi_i(\varphi) + \sqrt{B}\eta \\
=& \frac{U}{S}\sum_i \frac{dJ_{0,i}}{d\varphi}\frac{1+\tau_u\phi_{0,i}\left(U\tau_u\phi_{0,i}+2\right)}{\left(U\phi_{0,i}\left(\tau_u\tau_x\phi_{0,i}+\tau_u+\tau_x\right)+1\right)^2}\Delta\phi_i(\varphi) + \sqrt{B}\eta,
\end{aligned}$$

where we have used Eqs. (S22) and (S23), as well as Eq (S17) in the last equality.

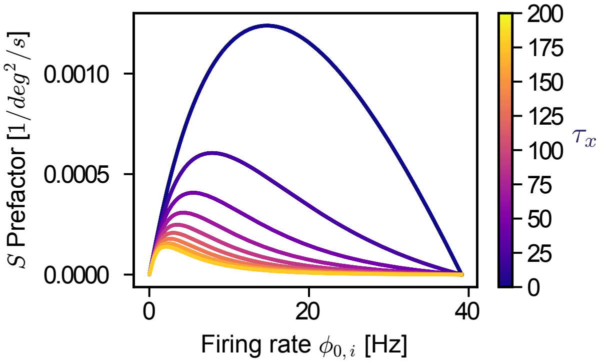

Fig S1-1. Pre-factor of normalizer for vanishing facilitation. Pre-factor $\frac{dJ_{0,i}}{d\varphi}^2 \phi'_{0,i} \frac{1}{(1+\phi_{0,i}\tau_x)^3}$ for varying firing rates $\phi_{0,i}$ and depression time constant τ_x. Color legend on the right hand side shows values of τ_x in units of ms.

6 Critical depression time constant

To analyze the vanishing normalization constant for growing depression time constant τ_x (see Supplementary Figure S8 Fig), we set $U = 1$ in the normalization constant S (Eq (19) of the main text), which yields:

$$S = \sum_i \frac{dJ_{0,i}}{d\varphi}^2 \phi'_{0,i} \frac{(\phi_{0,i}\tau_s\tau_x - \phi_{0,i}\tau_x^2 + \tau_s)}{(1+\phi_{0,i}\tau_x)^3}. \tag{S28}$$

Inspecting Eq (S28), we find that S will be crossing to zero if $\phi_{0,i}\tau_s\tau_x - \phi_{0,i}\tau_x^2 + \tau_s \leq 0$ for many summands. Solving this at equality for τ_x yields a single positive solution

$$\tau_{x,i} = \frac{1}{2}\left(\tau_s + \sqrt{\frac{\tau_s(\phi_{0,i}\tau_s + 4)}{\phi_{0,i}}}\right), \tag{S29}$$

which approaches τ_s for large firing rates $\phi_{0,i} \to \infty$. Thus, summands of Eq (S28) with larger firing rates $\phi_{0,i}$ will be the first to turn negative if $\tau_x > \tau_s$. As τ_x grows further, also summands with smaller firing rates will become negative according to Eq (S29), eventually turning the total sum to zero. On the other hand, the range of firing rates that contribute at all to Eq (S28) is limited to the flanks of the firing rate profile: the pre-factors $\frac{dJ_{0,i}}{d\varphi}^2 \phi'_{0,i}$ in Eq (S28) will vanish for $\phi_{0,i} \to 0$ and $\phi_{0,i} \to \max_i \phi_{0,i}$, since in both cases $\frac{dJ_{0,i}}{d\varphi} = 0$. This interplay of single summands turning negative and their contributing only in bump-shape dependent ranges is hard to generally analyze further. However, the relation found in Eq (S29) is valid for any bump system with depression, and will eventually lead to a vanishing normalization factor S.

We will now resort to a numerical analysis for the spiking system used in the main text. Plotting the pre-factor $\frac{dJ_{0,i}}{d\varphi}^2 \phi'_{0,i} \frac{1}{(1+\phi_{0,i}\tau_x)^3}$ against the firing rate $\phi_{0,i}$ for varying τ_x (see Fig (S1-1)), we see that as τ_x increases beyond $50ms$, the range of firing rates with positive contributions to the sum quickly decays to between 0 and 15Hz, with maxima between 2Hz and 5.5Hz. Evaluating Eq (S29) for $\tau_s = 100ms$ at these firing rates, the corresponding values of $\tau_{x,i}$ in Eq (S29) are $279.1ms$ (for 2Hz) and $193.8ms$ (for 5.5Hz). This only yields an estimate of the range in which summands will switch to negative values, and not the value of the total sum in Eq (S28). A numerical solution of Eq (S28) yields the depression time constant at which Eq (S28) becomes zero: $\tau_x, c = 223.9$ms, which nevertheless lies in the thus estimated range.

References

1. Burak Y, Fiete IR. Fundamental Limits on Persistent Activity in Networks of Noisy Neurons. Proceedings of the National Academy of Sciences. 2012;109(43):17645–17650.
