# Supplementary material for: Stability of working memory in continuous attractor networks under the control of short-term plasticity
Source: PLoS Comput Biol. 2019 Apr 19;15(4):e1006928. doi: 10.1371/journal.pcbi.1006928 (PMC6493776; doi:10.1371/journal.pcbi.1006928)
Supplement: S1 Table — Parameter values are modified from [11] and [50]. For recurrent conductances see the table in S2 Table. (PDF) [file pcbi.1006928.s010.pdf]

S1 Table. Parameters for spiking simulations

Parameter name	Parameter Symbol	Units	Excitatory neurons	Inhibitory neurons
Neuron number	$N_\bullet$	1	$N_E = 800$	$N_I = 200$
Poisson neuron number	N_{ext}	1	1000	1000
Frequency of Poisson neurons	ν_{ext}	Hz	2.6	2.6
Membrane capacitance	C_m	pF	500	200
Exc. reversal potential	V_E	mV	0	0
Inh. reversal potential	V_I	mV	-70	-70
Leak reversal potential	V_L	mV	-70	-70
After spike reset potential	V_{res}	mV	-60	-60
Spiking threshold	V_{thr}	mV	-50	-50
External conductance	g_{ext}	nS	2.08	1.62
Leak conductance	g_{L}	nS	25	20
External synaptic timescale	τ_{ext}	ms	2	2
Recurr. inh. timescale	τ_I	ms	10	10
Recurr. exc. timescale	τ_s	ms	100	100
Membrane time constant $\frac{C_m}{g_L}$	τ_m	ms	20	10
Refractory period	τ_{ref}	ms	2	1

Parameter values are modified from [1] and [2]. For recurrent conductances see the table in **S2 Table**.

References

1. Compte A, Brunel N, Goldman-Rakic PS, Wang XJ. Synaptic Mechanisms and Network Dynamics Underlying Spatial Working Memory in a Cortical Network Model. Cerebral Cortex. 2000;10:910–923.

2. Brunel N, Wang X. Effects of Neuromodulation in a Cortical Network Model of Object Working Memory Dominated by Recurrent Inhibition. Journal of Computational Neuroscience. 2001;11:63–85.
