# Supplementary material for: Stability of working memory in continuous attractor networks under the control of short-term plasticity
Source: PLoS Comput Biol. 2019 Apr 19;15(4):e1006928. doi: 10.1371/journal.pcbi.1006928 (PMC6493776; doi:10.1371/journal.pcbi.1006928)
Supplement: S2 Table — For all networks we set w+ = 4.0. Recurrent conductance parameters are given for combinations of short-term plasticity parameters according to the following notation. gEE: excitatory conductance gE on excitatory neurons; gIE: excitatory conductance gE on inhibitory neurons; gEI: inhibitory conductance gI on excitatory neurons; gII: inhibitory conductance gI on inhibitory neurons. (PDF) [file pcbi.1006928.s011.pdf]

S2 Table. Conductance and connectivity parameters for spiking simulations.

STP U [1]	STP τ_u [ms]	STP τ_x [ms]	g_{EE} [nS]	g_{IE}[nS]	g_{EI}[nS]	g_{II}[nS]	σ_w[rad]
1	650	150	0.03489	0.004975	2.639	1.637	0.38
0.8	650	150	0.03421	0.005167	2.537	1.641	0.38
0.6	650	150	0.0328	0.005485	2.418	1.647	0.4
0.4	650	150	0.0315	0.00529	2.295	1.643	0.4
0.2	650	150	0.03356	0.005349	2.18	1.644	0.35
0.1	650	150	0.03393	0.005906	2.107	1.655	0.4
0.08	650	150	0.03441	0.005746	2.092	1.652	0.4
0.06	650	150	0.03646	0.006018	2.079	1.657	0.4
0.04	650	150	0.03771	0.005764	2.062	1.652	0.42
0.1	650	140	0.03243	0.004679	2.103	1.632	0.33
0.1	650	120	0.02972	0.004423	2.097	1.627	0.3
0.1	650	160	0.03606	0.006294	2.112	1.662	0.41
0.1	650	180	0.04054	0.005043	2.123	1.639	0.32
0.1	650	200	0.04547	0.005479	2.134	1.647	0.32
0.4	650	140	0.03094	0.005342	2.291	1.644	0.37
0.4	650	160	0.03557	0.005666	2.33	1.65	0.37
0.4	650	120	0.02882	0.005507	2.274	1.647	0.34
0.4	650	180	0.03841	0.005654	2.352	1.65	0.39
0.4	650	200	0.04315	0.005914	2.391	1.655	0.39
0.8	650	120	0.02738	0.004926	2.44	1.636	0.38
0.8	650	140	0.03171	0.005033	2.502	1.638	0.38
0.8	650	160	0.03682	0.005294	2.574	1.643	0.38
0.8	650	180	0.03829	0.005065	2.591	1.639	0.415
0.8	650	200	0.0419	0.005046	2.64	1.639	0.425
0.8	1000	150	0.03433	0.005176	2.548	1.641	0.38
0.6	1000	150	0.03371	0.005401	2.445	1.645	0.38
0.4	1000	150	0.03273	0.005514	2.324	1.647	0.38
0.2	1000	150	0.03346	0.006019	2.195	1.657	0.37
0.1	1000	150	0.03295	0.006211	2.113	1.66	0.41
0.08	1000	150	0.03292	0.006113	2.097	1.659	0.42
0.06	1000	150	0.03353	0.006124	2.08	1.659	0.43
0.04	1000	150	0.03517	0.006177	2.064	1.66	0.44

For all networks $w_+ = 4.0$. Recurrent conductance parameters are given for combinations of short-term plasticity parameters according to the following notation: g_{EE}: excitatory conductance g_E on excitatory neurons; g_{IE}: excitatory conductance g_E on inhibitory neurons; g_{EI}: inhibitory conductance g_I on excitatory neurons; g_{II}: inhibitory conductance g_I on inhibitory neurons.
